# Towards helical-chirality-controlled molecular motors

**DOI:** 10.1039/d6sc00373g

**Published:** 2026-04-29

**Authors:** Yohan Gisbert, Eric Sidler, Ben L. Feringa

**Affiliations:** a Stratingh Institute for Chemistry, University of Groningen Nijenborgh 3 9747 AG Groningen The Netherlands yohan.gisbert@univ-rennes.fr b.l.feringa@rug.nl

## Abstract

Photochemically driven overcrowded-alkene-based molecular motors have become ubiquitous in molecular nanotechnology due to their reliable unidirectional rotation, modular design, and responsiveness to light. For all the designs of such motors reported to this day, the directional bias of the motion is a consequence of the stereochemistry of the molecule, defined by one or multiple (pro-)chiral stereogenic centers. Such designs nevertheless suffer from incompatibility with various potential experimental conditions, involving for instance the use of strong bases or redox-stimuli. To expand the application scope of molecular motors, we envisioned to replace this point-chirality by a stable helical element, directly integrated within the structure of the molecular motor and use it as the sole inherent chiral information used to drive the unidirectional rotation of the overcrowded-alkene-based motor. Herein we report the design, synthesis, and characterization of three overcrowded-alkenes featuring a helical half, accompanied by a detailed study of their rotational properties, showing the importance of a subtle interplay of distinct isomerization processes.

## Abstract 2

Helical chirality is integrated into overcrowded-alkene motors as the sole chiral element, and the subtle interplay of the isomerization processes that governs the resulting rotational behavior is studied for every new motor design.

## Graphical abstract

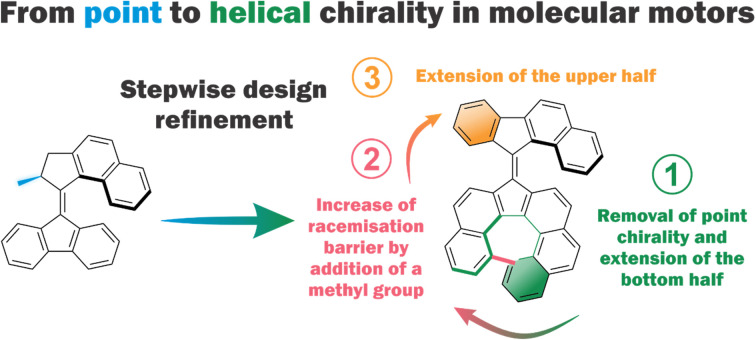

## Introduction

Molecular machines, and specifically molecular motors,^1^ are omnipresent in Nature.^2^ The remarkable sophistication of biological machinery has driven chemists over the past three decades to pioneer the creation of artificial molecular machines, aiming to replicate, and even surpass, the precision and efficiency found in Nature.^3,4^ Since the creation of the first light-driven artificial molecular motor by our group more than 25 years ago,^5^ many new designs, such as hemithioindigos,^6^ imine-,^7^ and azobenzene^8^-based motors were successfully developed and studied. However, overcrowded-alkene-based molecular motors remain the most widely used systems for practical applications as they are studied in-depth and possess numerous attractive properties ranging from great stability to very high directionality efficiency.^9,10^

Taking profit of the dissipative nature of their light-fueled rotation mechanism allowing to access states out of thermodynamic equilibrium,^11^ light-driven molecular motors are now used as an efficient way to produce controlled motion at the nanoscale. By integrating molecular motors in more complex systems, ranging from polymers^12^ to liquid crystals,^13,14^ their movement was efficiently transmitted across length-scales to result in motion that is observable at the microscopic or macroscopic level.

**Scheme 1:**
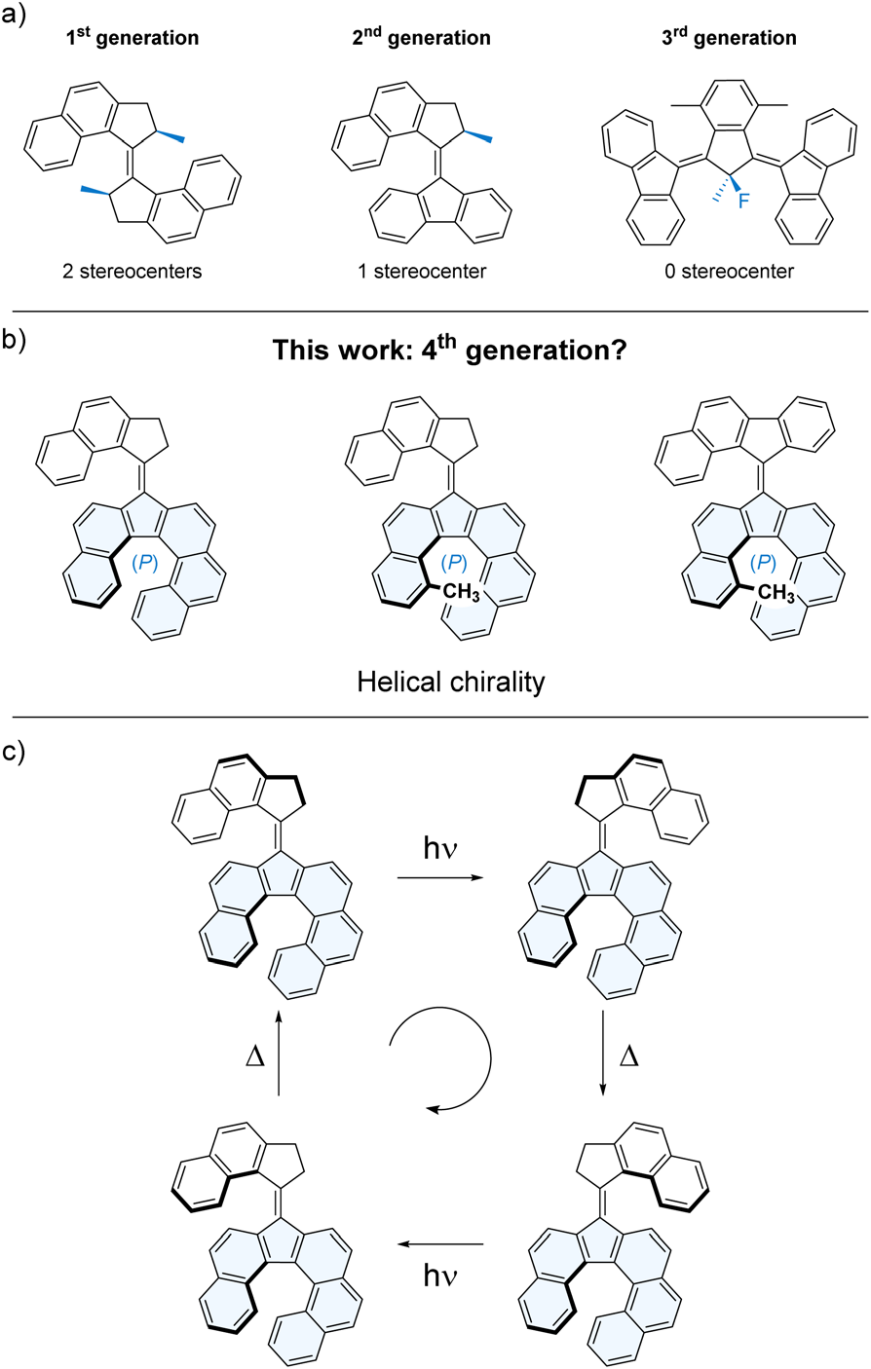
(a) Reported light-driven overcrowded-alkene-based molecular motors, featuring point chiral or prochiral centers (in blue). (b) Proposed molecular motor structures relying on a helicene (in blue) as the sole chiral element. (c) Proposed rotation cycle for a helicity-driven overcrowded-alkene-based molecular motor.

To employ molecular motors in varying environments, a diverse toolbox of motors with different properties and functionalities is crucial to take advantage of their full potential. Therefore, it is important to develop well-designed derivatives of molecular motors, as fine variations of the design usually result in drastic changes of the dynamic properties. Various more or less subtle modifications of the scaffold of overcrowded-alkene-based motors were reported over the years. These include, modifying the size or nature of the rings on either half of the overcrowded alkene,^15^ changing the hindrance within the fjord region,^16–18^ or tuning the electronic properties of the system in order to modulate selectivity, efficiency and speed of the motors.^19^ The most fundamental changes of the structure and properties of the overcrowded alkene-based molecular motors were obtained by modifying the number of stereogenic centers used to drive the unidirectional rotation of the motors (Scheme 1a). The first series of motors to be developed (so-called 1st generation motors) featured two stereogenic centers.^5^ Subsequently, our group developed 2nd generation molecular motors featuring only one stereogenic center,^20–22^ finally followed by 3rd generation molecular motors,^23^ which did not contain any well-defined stereocenter but were able to perform a conrotatory directional motion of two rotors, owing to its pseudo-chirality.

Our recent work on increasing the degree of functionality of molecular motors led us to develop a new family of second-generation motors comprising a helical lower half which was shown to undergo dynamic helical inversions during the rotation cycle of the motor through a chirality transfer mechanism enabled by a coupled (or geared) motion phenomenon.^24^ Based on this work, we envisioned that increasing steric hindrance within the helicene lower half would result in a stable helically chiral element within the motor scaffold (Scheme 1b), which might allow to drive the unidirectional rotation of the motor without the need for a (pro-)chiral center in the upper half (Scheme 1c).

Aside from the fundamental interest of being able to drive the unidirectional rotation of a motor in an unprecedented fashion, using exclusively helical chirality could open the way for a range of new applications. For instance, it was shown that the stereocenters from our previous designs were not compatible with redox chemistries due to the presence of acidic alpha protons that resulted in epimerization and subsequent loss of directionality,^25^ which might be circumvented by using helicity as the sole chiral element. Moreover, integrating a helicene directly within the scaffold of our molecular motors would allow to combine the properties of these dynamic overcrowded alkene-based motors with the very attractive properties of helicenes, such as very high dissymmetry factors resulting in intense electronic circular dichroism (ECD) and circularly polarized luminescence (CPL), while enabling stimuli-responsive tuning.^26^ Helicenes are chiral elements of choice for applications in optoelectronics^27^ and spintronics.^28^ Having a motor with large dissymmetry factors would further allow to drive the motor function efficiently using circularly polarized light and potentially enables different responsiveness depending on the chirality of the polarized light stimulus.

Herein we present our efforts in designing such helical-chirality-controlled molecular motors, with the synthesis of three helically chiral derivatives, the study of their properties and our attempts at using them as light-driven molecular motors. Furthermore, our in-depth computational analysis using density functional theory (DFT) can serve as a valuable guideline for future design strategies.

## Results and discussion

### First strategy: π-extension of the lower half

**Scheme 2:**
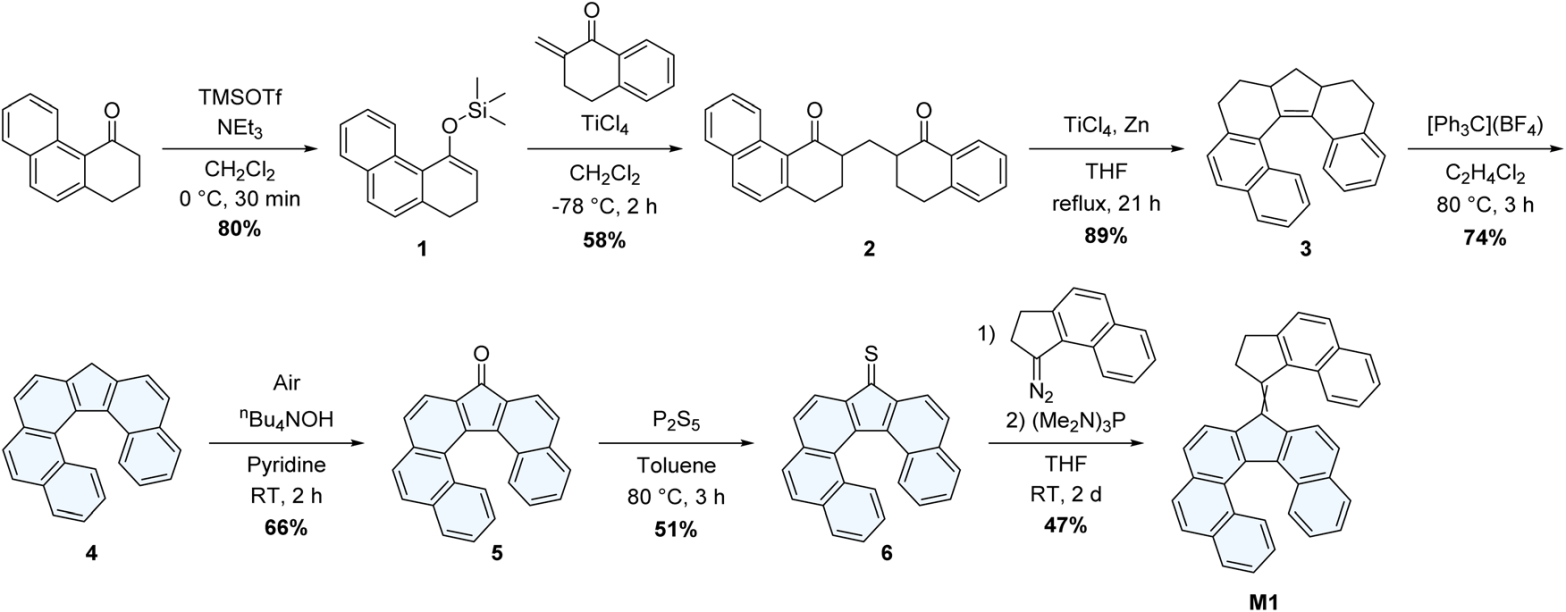
Synthesis of the proposed molecular motor M1 featuring a helicene lower half as the sole chiral element.

The most straightforward strategy for designing helically chiral derivatives of molecular motors is to prepare a direct analogue of the prototypical 2nd generation model, in which the stereogenic center is removed, and a helical fragment is introduced by extending the aromatic system of the lower half (Scheme 1c). In order to demonstrate the unidirectional rotation of the motor experimentally, the lower half of the motor must be dissymmetric,^1,21^ thus yielding *E* and *Z* isomers of the overcrowded alkene, allowing for a differentiation of productive thermal helix inversion (THI) steps from non-productive thermal *E*/*Z* (TEZ) isomerizations. We thus decided to use a dissymmetric helically extended fluorene analogue as the lower half, yielding our initial molecular design M1 (Scheme 2).

The synthesis of the lower half of M1 is based on our previous work on helicene-functionalized molecular motors,^24^ which was inspired by seminal work on the preparation of symmetrical analogues of extended dibenzo[*c*,*g*]fluorenes.^29^M1 was prepared in seven steps (Scheme 2), starting from 2,3-dihydrophenanthren-4(1*H*)-one.^30^ The extended α-tetralone was first converted to the corresponding silyl enol ether 1, which was then used to perform a Mukaiyama–Michael addition on β-methylene-α-tetralone,^31^ affording the 1,5-diketone 2. The resulting diketone was then submitted to intramolecular McMurry cross-coupling conditions, to afford the reduced π-extended fluorene analogue 3, which was subsequently oxidized with tritylium tetrafluoroborate to afford fluoreno[6]helicene 4, followed by an oxidation to the corresponding π-extended fluoreno[6]helicenone derivative 5. The latter was reacted with P_2_S_5_, yielding the thioketone 6. M1 was finally assembled *via* a Barton–Kellogg coupling of thioketone 6 and the diazo-functionalized upper half obtained by *in situ* oxidation of the already reported (2,3-dihydro-1*H*-cyclopenta[*a*]naphthalen-1-ylidene)hydrazone^32^ to yield M1 in 47% yield as a mixture of the eight possible isomers (Fig. S1) that were virtually inseparable using standard column chromatography.

The presence of a complex mixture of four non-equivalent isomers was proven by ^1^H NMR spectroscopy (Fig. S40–S44), where two major and two minor isomers were observed that can be ascribed to the stable and metastable states of M1, respectively. The presence of several isomers makes the interpretation of the ^1^H NMR signals very challenging and does not allow clean integration of individual states and therefore studying its rotational cycle in a well-defined manner. We attempted to separate the isomers using HPLC, but purification using either a chiral or achiral stationary phase was impractical due to significant overlap of peaks given the presence of a total of up to 8 isomers in the mixture, when taking all enantiomers into account (Fig. S1).

We thus decided to perform the chiral separation of the π-extended fluorenone derivative 5 first, which would allow to synthesize motor M1 in an enantiopure fashion. During the optimization of the analytical conditions to separate (*M*)-5 and (*P*)-5 on a chiral stationary phase, we observed the formation of a plateau in between both peaks at 40 °C column-oven temperature, which indicates a low enantiomerization barrier that can be determined through dynamic chromatography. Performing the separation at an oven temperature of 70 °C allowed to determine an enantiomerization barrier of Δ*G*^‡^_e-__5__-exp_ = 103 kJ mol^−1^ at 70 °C for 5 through analysis of the elution profile according to literature protocols^33^ (see SI page S31).

DFT calculations at the r^2^SCAN-3c ^34^/CPCM(CH_2_Cl_2_)^35^ level of theory were performed with the ORCA 5.0.3 software,^36^ confirming that the helicene inversion barrier of fluorenone 5 is indeed rather low with a calculated Δ*G*^‡^_e-__5__-calc_ of 106.8 kJ mol^−1^ and a half-life of 6.6 days at 25 °C, in well-agreement with the value obtained by dynamic chromatography. Calculations at the same level of theory were also performed on M1 to assess its configurational stability (Fig. S19). Indeed, these calculations showed a lower-half helicene inversion (LHI) barrier of Δ*G*^‡^_LHI-*Z*-__M1_ = 109.5 kJ mol^−1^ for the *Z*-metastable state, indicating a small influence of the upper half on the configurational stability of the lower half. At the same time, calculations suggested a thermal helix inversion barrier of Δ*G*^‡^_THI-*Z*-__M1_ = 107.3 kJ mol^−1^ when going from the *Z*-metastable to the *Z*-stable state, which is a crucial step in the productive unidirectional rotation cycle. This THI barrier is only 2.2 kJ mol^−1^ lower than the barrier of the undesired LHI, which therefore leads to an overall loss of chiral information and thus directionality simultaneous to the productive rotational cycle. Moreover, the energy difference between stable and so-called “metastable” states is relatively small: only 5.9 kJ mol^−1^ for *E*-metastable/*E*-stable and no difference for *Z*-stable/*Z*-“metastable”. As both *Z* states thus just equilibrate to equimolar concentrations upon relaxation, half of the rotation cycle should not display any directionality, resulting in a diminished overall directional efficiency over the rotation cycle if both, *Z*-stable and *Z*-“metastable”, are displaying similar absorption spectra, extinction coefficients and photoisomerization quantum yields.

These findings suggest the design of M1 to be suboptimal for driving the unidirectional rotation, due to a low LHI barrier. Moreover, a lack of steric hindrance within the fjord region induces a small energy difference between stable and metastable states, resulting in a decreased directionality.^37^ We thus sought to refine our design with the help of predictive DFT calculations.

### Varying the energy landscape *via* upper-half modifications

**Fig. 1:**
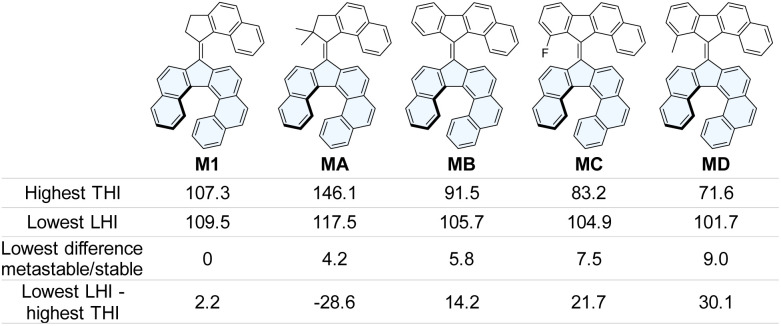
Structures of the helicene-containing overcrowded-alkenes investigated by DFT calculations, and associated highest THI barrier, lowest helicene inversion barrier and lowest difference between stable and metastable states. The Gibbs free energy difference values were calculated at the r^2^SCAN-3c/CPCM(CH_2_Cl_2_) level of theory. Energies are given in kJ mol^−1^ at 25 °C. An efficient motor should display a highest THI barrier significantly smaller than the lowest helicene inversion barrier, and a considerable energy difference between stable and metastable states.

While the increase of the LHI barrier is achieved by increasing the steric hindrance within the helicene moiety, optimizing the steric hindrance within the fjord region while preserving an achiral upper half requires more thorough design engineering. Putative structures MA, MB, MC and MD (Fig. 1), introducing new upper-halves, were thus studied computationally, with the aim of increasing the difference between the energies of the stable and metastable states, while decreasing the THI barrier. Knowing the theoretical value of this THI barrier would then allow to tune the steric hindrance within the lower half to obtain a LHI barrier significantly higher than the THI.

**Fig. 2:**
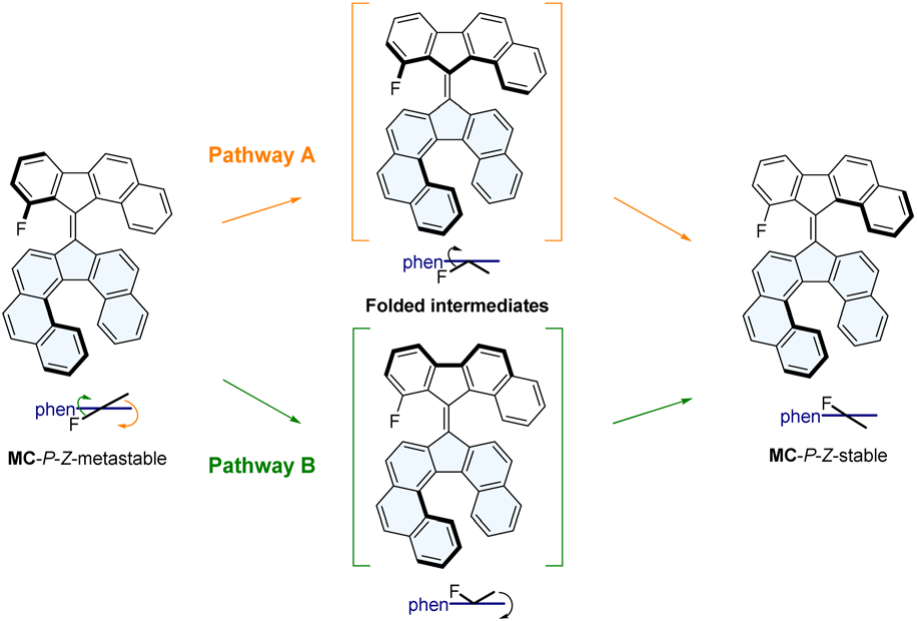
Calculated mechanism of the THI of the *Z* isomer of MC, involving two possible pathways. Pathway A (in orange) first involves the helix inversion of the naphthyl moiety of the upper half, followed by the helix inversion of the fluorinated moiety. Pathway B (in green) first involves the helix inversion of the fluorinated moiety of the upper half, followed by the helix inversion of the naphthyl moiety.

For M1, MA and MB, the calculated rotation mechanism is very similar to the one of regular second-generation motors, featuring four distinct states, two stable ones and two “metastable” ones (here sometimes only slightly higher in energy) which are populated upon photoirradiation (Fig. 1, S20 and S21). Nevertheless, MA displayed a THI barrier higher than the LHI barrier, which would favor the latter over directional rotation. In contrast, MB displays an energy difference between the highest THI and LHI barrier of 14.2 kJ mol^−1^, which is a significant improvement in comparison to prototype M1. For the more hindered MC and MD, the rotation cycles become much more complicated with additional unstable folded intermediates being transiently populated during the THI. These intermediates are a consequence of the increased steric crowding in the fjord region of the overcrowded-alkene, resulting in the stepwise rotation of both sides of the upper-half during the THI (Fig. 2 and S22).

**Scheme 3:**
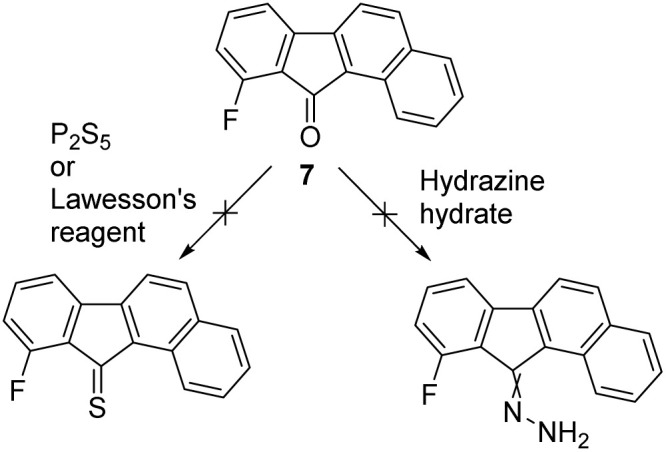
Attempts to convert fluorinated benzofluorenone precursor 7 into the corresponding thioketone or hydrazone as precursors for Barton–Kellogg couplings.

From these calculations, it seems that MC and MD are both good candidates, with MC being the most convenient to study experimentally due to the presence of a fluorine atom which could be used as a probe to study the rotation *via*^19^F NMR.^24,38^ The corresponding fluorinated benzofluorenone precursor 7 was not reported in the literature but could be prepared in a single high-yielding step by a palladium-catalyzed domino *ortho*-arylation/aldehyde addition sequence developed by Lautens *et al.*^39^ Nevertheless, despite our efforts we did not succeed in preparing the corresponding hydrazone or thioketone derivatives that could be used as precursors for the Barton–Kellogg coupling to form the overcrowded-alkene MC, as all of our attempts resulted in the decomposition of the substrate (Scheme 3). Therefore, we decided to focus on the analogue MB featuring no fluorine atom and less steric hindrance next to the ketone. Non-fluorinated analogue MB, is predicted to have a lower energy difference between its stable and metastable states, but an improvement compared to the previously synthesized M1.

### Increasing the gap between both helix inversion barriers

**Scheme 4:**
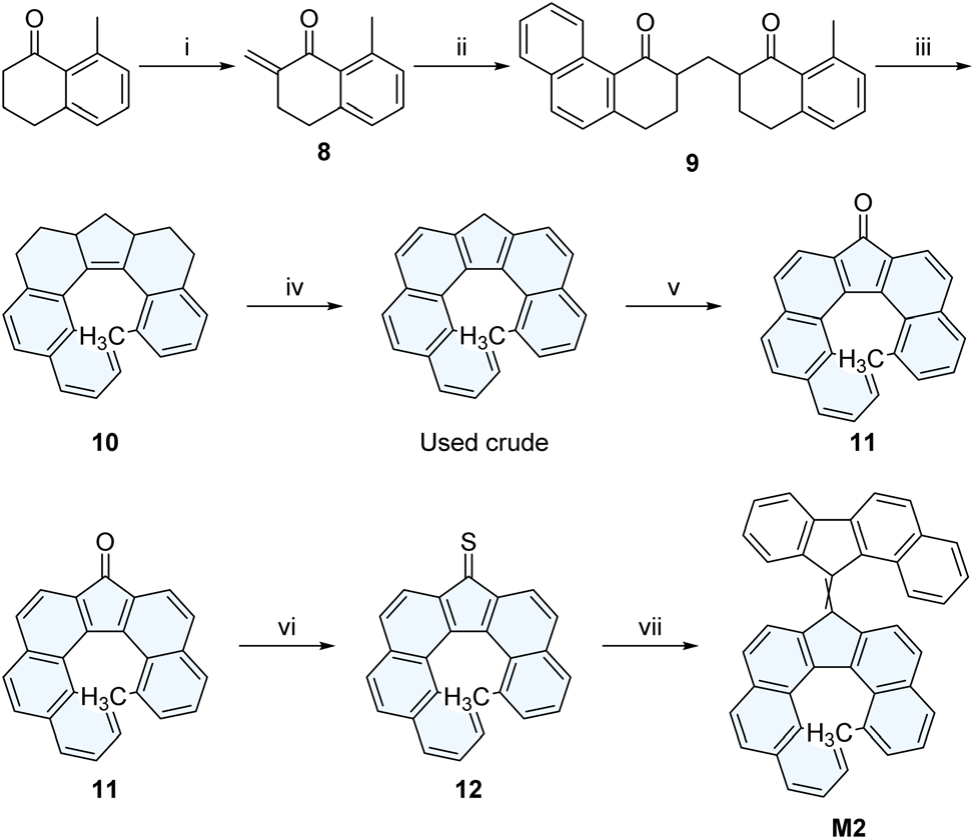
Synthesis of the methylated lower half 11 and of M2. Experimental conditions: (i) paraformaldehyde, *N*-methylanilinium trifluoroacetate, THF, reflux, 4.5 h, 75%; (ii) 1, TiCl_4_, CH_2_Cl_2_, −78 °C, 2 h, 49%; (iii) TiCl_4_, Zn, THF, reflux, 20 h, 57% (iv) [PH_3_C](BF_4_), C_2_H_4_Cl_2_, 80 °C, 2 h, used crude; (v) air, ^*n*^Bu_4_NOH, pyridine, RT, 2 h, 49% (2 steps) (vi) P_2_S_5_, toluene, 80 °C, 3 h, 81% (partial purification); (vii) 1.11-diazo-11*H*-benzo[*a*]fluorene, THF, RT, 24 h, 2. (Me_2_N)_3_P, THF, RT, 24 h, 51%.

To prevent the racemization of the lower helicene half (*via* LHI), we additionally designed a new helically chiral benzofluorene derivative by incorporating an additional methyl substituent within the fjord region of the helicene. The helically chiral lower half 11 (Scheme 4) was prepared in an analogous fashion to the synthesis of 5 (Scheme 2).

DFT calculations at the r^2^SCAN-3c/CPCM(CH_2_Cl_2_) level of theory allowed to determine an inversion barrier of Δ*G*^‡^_e-__11_ = 144 kJ mol^−1^ for the methylated lower half 11, corresponding to a half-life of 6.0 × 10^4^ years at 25 °C, resulting in a drastic increase in half-life compared to the non-methylated counterpart 5.

Molecular motor prototype M2 was then synthesized by first generating the thioketone 12 derived from the methylated helicene lower-half 11 and subsequent Barton-Kellogg coupling with 11-diazo-11*H*-benzo[*a*]fluorene^40^ (Scheme 4).

**Fig. 3:**
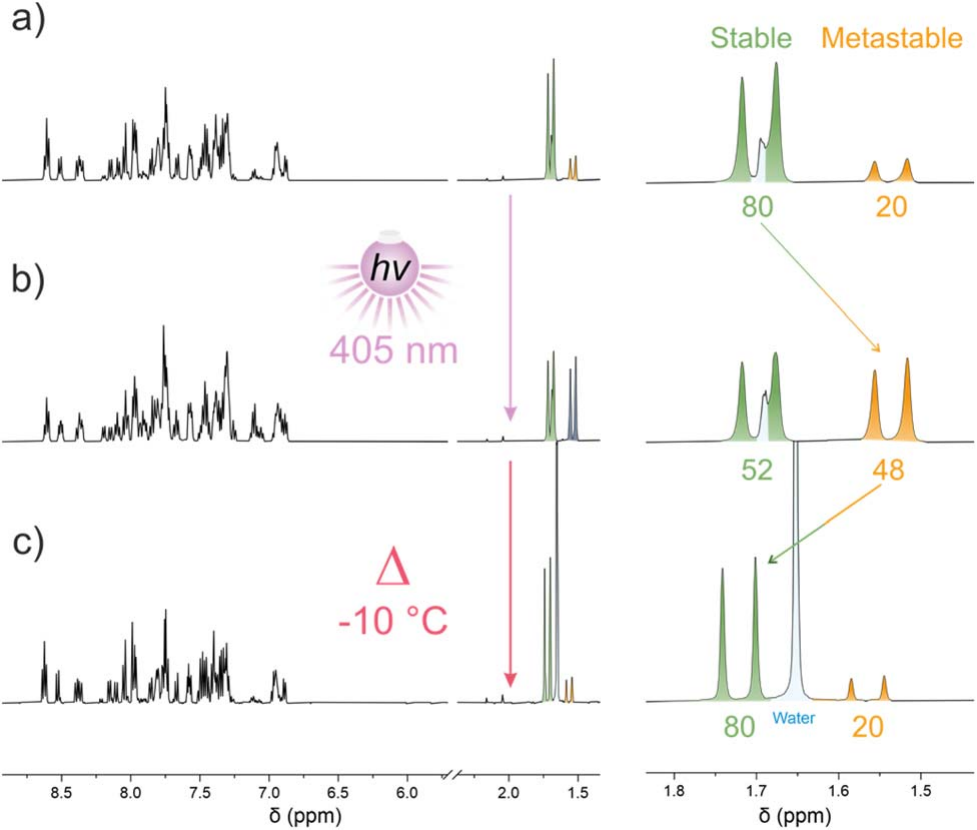
*In situ* ^1^H NMR (500 MHz, CD_2_Cl_2_) irradiation with 405 nm light of an isomeric mixture of M2 at −45 °C followed by thermal relaxation (THI) at −10 °C. (a) Initial mixture, (b) at PSS after irradiation with 405 nm light at −45 °C, (c) after relaxation at −10 °C.

After purification of target M2, ^1^H NMR spectroscopy indeed showed the presence of four distinct isomers in a *ca.* 1 : 1 : 0.25 : 0.25 ratio (80 : 20 stable/metastable) which could be differentiated using the characteristic methyl signal of the lower-half (Fig. 3a).

Irradiation of the mixture in CD_2_Cl_2_ at −45 °C with 405 nm light resulted in the partial conversion of the stable isomers into their metastable counterparts to reach a 52 : 48 stable/metastable PSS ratio (Fig. 3b). Increasing the temperature to −10 °C resulted in an apparent THI process yielding the original isomeric mixture (Fig. 3c).

This thermal decay was monitored by ^1^H NMR spectroscopy at five different temperatures, and an Eyring analysis allowed us to determine the Gibbs free energy of activation at 20 °C of Δ*G*^‡^_THI-iso1-__M2_ = 81.17 ± 0.94 kJ mol^−1^ for the most shielded isomer (1.52 ppm) corresponding to a half-life time of 33 s at the same temperature (Fig. S4) and a similar value of Δ*G*^‡^_THI-iso2-__M2_ = 82.57 ± 1.97 kJ mol^−1^ for the less shielded isomer (1.56 ppm). This free energy value is somewhat comparable to the one obtained by DFT calculations of Δ*G*^‡^_THI-*Z*-__M2__-calc_ = Δ*G*^‡^_THI-*E*-__M2__-calc_ = 92 kJ mol^−1^ (Fig. S25).

**Fig. 4:**
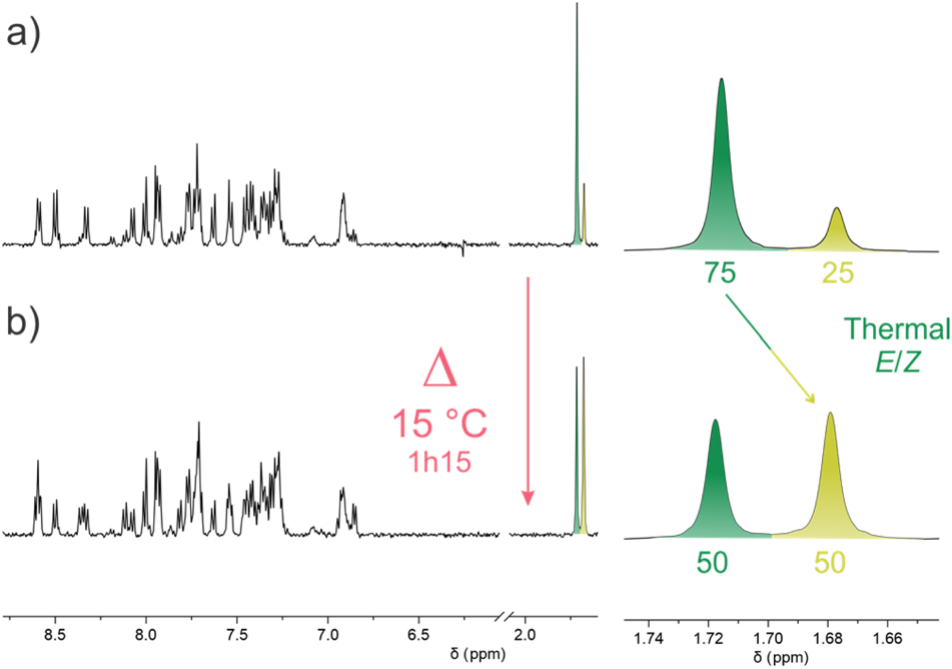
^1^H NMR evidence (500 MHz, CD_2_Cl_2_) of the spontaneous TEZ isomerization of M2 at 15 °C. (a) Initial state, (b) after 1h15.

In order to confirm that the observed photoisomerization and thermal relaxation steps result in the unidirectional rotation of M2, we then aimed to separate its *E* and *Z* isomers in order to investigate the stepwise rotation over the whole cycle and to rule out potentially detrimental TEZ isomerisation processes. Nevertheless, for this particular overcrowded-alkene derivative, chromatographic methods (manual or HPLC) proved to be inefficient in obtaining samples enriched in one isomer or the other. Performing HPLC on a chiral stationary phase, however, allowed to resolve M2 into two separate peaks, which we initially expected to be the *E* and *Z* isomers (Fig. S11). However, based on the mirror CD spectra of the isolated peaks *via* semipreparative HPLC, the peaks were identified to be (*M*)-M2 and (*P*)-M2 instead (Fig. S14). Gratifyingly, slow precipitation of M2 from a concentrated CH_2_Cl_2_ solution by slow diffusion of a methanol top-layer allowed us to obtain a mixture highly enriched with a single isomer, as confirmed by the integral ratio of the methyl groups in the ^1^H NMR spectrum (Fig. 4). However, at room temperature and in the dark, the ratio of both isomers quickly equilibrated back to a 1 : 1 mixture of *E* and *Z* isomers as initially synthesized (Fig. 4), *via* a TEZ isomerisation. The half-life time of this process was found to be *t*_1/2_ = 25.7 ± 1.6 min at 15 °C (Fig. S6) corresponding to a Gibbs free energy of activation of *ca.* Δ*G*^‡^_TEZ-__M2_ = 89 kJ mol^−1^ at the same temperature.

This TEZ process is thus detrimental to the unidirectional rotation of M2, resulting in the rapid equilibration of both *E* and *Z* isomers under ambient conditions, rendering the experimental characterization of the unidirectional rotation impractical and tedious, as, despite our efforts, we could only enrich one of the two isomers. The facile TEZ is furthermore providing explanation as to why separation using conventional chromatographic methods was unsuccessful. We ascribe this TEZ process to the bifluorenylidene structure of M2, which results in an increased steric hindrance within the fjord region and an augmented planarity of the upper half compared to conventional second-generation motors. Nevertheless, we determined an energy barrier (Δ*G*^‡^_THI-__M2_) of *ca.* 80 kJ mol^−1^ for the productive THI process (*vide supra*), which, extrapolated to 15 °C, would correspond to a half-life time *t*_1/2_ = 37 s, ∼40 times faster than the unproductive TEZ process at the same temperature. This means that M2 can indeed provide a directional rotative motion at low temperature, but the study of its entire rotation cycle in a stepwise manner was impossible due to a fast TEZ prohibited under ambient conditions and rather low PSS ratios.

### Combination of both designs

In a subsequent attempt to produce and evidence unidirectional rotation driven by helicity as the sole chiral element, the two previous designs of M1 and M2 were combined by using the upper half of M1 but the methylated lower-half of M2 to prevent racemization. From the calculations performed on M1 (Fig. S19), half of the rotation cycle (*E* isomer) should be directional while the other half should be non-directional due to the similar stability of the stable and “metastable” states of the *Z* isomer. Overall, this should lead to a direction bias in the rotation cycle, if the chirality of the lower half can be retained, *i.e.* LHI is prevented.

**Scheme 5:**
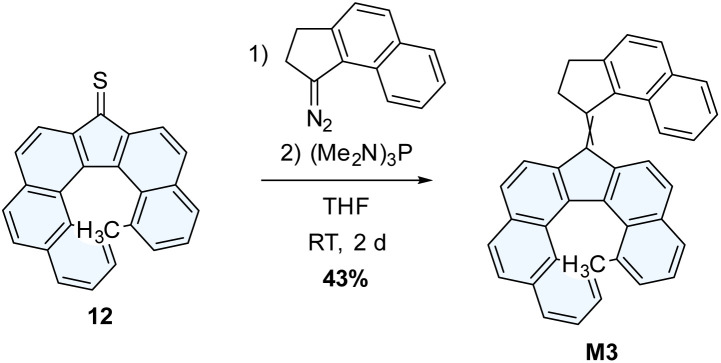
Synthesis of M3.

M3 was synthesized *via* a Barton–Kellogg coupling of the previously-used thioketone 12 and the *in situ* generated 1-diazo-2,3-dihydro-1*H*-cyclopenta[*a*]naphthalene (Scheme 5).

**Fig. 5:**
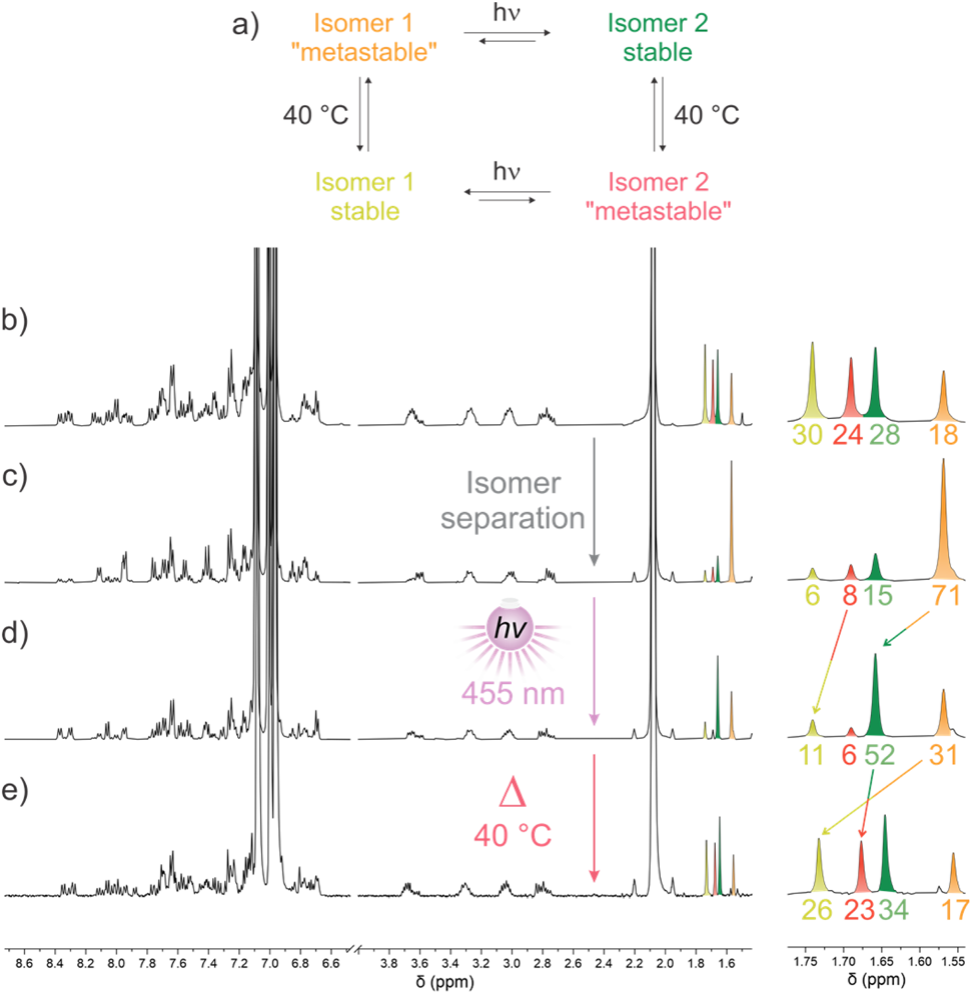
^1^H NMR (500 MHz, toluene-d8) spectra. (a) Switching cycle of M3. (b) Sample obtained after purification containing a mixture of 4 isomers (*E*/*Z*, stable/metastable) at 20 °C. (c) Sample enriched in a single metastable isomer of M3 at 15 °C. (d) PSS obtained after *in situ* irradiation with 455 nm light at 15 °C of the metastable isomer displayed above. (e) Subsequent thermal relaxation at 40 °C.

M3 was obtained as a mixture of four isomers (*E*/*Z*, stable/“metastable”), based on ^1^H NMR spectroscopy (Fig. 5a). *E* and *Z* isomers could be highly enriched by column chromatography (Fig. 5c and S61–S62), indicating no considerable TEZ under ambient conditions. The absence of a TEZ isomerization was further confirmed by HPLC on a chiral stationary phase where we observed the presence of 6 different peaks, wherein the fourth eluting peak consisted of multiple species based on the significant shouldering (Fig. S15). Using semipreparative columns, we then isolated the fifth and sixth eluting peak, which under ambient conditions equilibrated into two sets of different peaks, indicating that they converted into equilibrated mixtures of isomers with different chirality of the lower half (Fig. S16). This was further confirmed by the mirror CD spectra of the equilibrated mixtures (Fig. S17).

Chromatographic separation allowed to selectively enrich a single species: isomer 1 “metastable” (in orange, Fig. 5c). Upon irradiation of this “metastable” state with 455 nm light in toluene-d8 at 15 °C, a single stable state was generated (isomer 2 stable, in green), with a PSS ratio of 37 : 63 “metastable” : stable. During this irradiation, the minor isomer 2 “metastable” (in red) is also converting to isomer 1 stable (yellow, the process can be seen more clearly in Fig. S7). This suggests an asymmetry in the rotation cycle, with a hint of directionality provided by both photochemical steps.^41^ The thermal relaxation at 40 °C afforded a mixture composed of all four isomers, suggesting a small energy difference between stable and metastable isomers of both *E* and *Z* isomers (Fig. 5d and e). This mixture results from two simultaneous thermal equilibration processes. The partial conversion of stable isomer 2 (green) into “metastable” isomer 2 (red) is here productive with respect to the directionality of the photochemical steps. Conversely, the simultaneous equilibration of “metastable” isomer 1 (orange) back to its stable form (isomer 1 stable, yellow) is counterproductive, as this decay opposes the directionality of the other steps.

Irradiation of another isomeric mixture composed of both stable states in a *ca.* 75 : 25 ratio at 15 °C in toluene-d8 with 405 nm light resulted in the formation of the two corresponding “metastable” isomers at the same rate (Fig. S9). Noteworthily, at PSS, the stable states are still present in excess with respect to the metastable ones, as expected from the previous experiments (Fig. 5). Increase of the temperature to 40 °C did not produce any notable change (Fig. S10), which is likely the result of a PSS ratio similar to the Boltzmann distribution.

These results were confirmed by DFT calculations, showing that unlike for M1, for which half of the calculated cycle should result in directional bias driven by energetically downhill stable states compared to their metastable counterparts, M3 doesn't seem to show any significant difference in energy between stable and “metastable” states for both isomers (Fig. S26). More specifically, DFT predicted an energy difference of 1.0 and 1.9 kJ mol^−1^ for the *E* and *Z* isomers, respectively, which is close to the error margin of the calculation method and too small to efficiently induce a significant thermal decay, but rather lead to thermal equilibration between stable and “metastable” states.

Overall, under specific conditions, it is possible to observe directionality in the rotation cycle of M3 (Fig. 5), with an apparent preferential direction for 3 of the 4 steps when starting from a pure “metastable” isomer, mostly as a consequence of the preferential formation of the stable states upon irradiation at the chosen wavelength. Nevertheless, both photochemical and thermal steps favor the stable isomers, and no change in the ratio is observed when irradiating thermally equilibrated isomeric mixtures as the stable states are both favored thermally and “photochemically” with a ratio at thermal equilibrium close to the one observed at PSS. M3 is thus not able to produce a continuous, directional rotative motion, which would definitely qualify it as a molecular motor. Compared to other second-generation motors, the difference in energy between stable and metastable states is too small to provide significant ratcheting. We ascribe this lower difference in energy between the stable and “metastable” of M3 compared to the ones of M1 to an increased twisting of the lower half because of the introduction of the methyl substituent. This structural modification allowed to efficiently raise the racemization barrier of the helical lower half, but lowered the destabilization of the “metastable” state, thus preventing unidirectional rotation.

## Conclusions

**Fig. 6:**
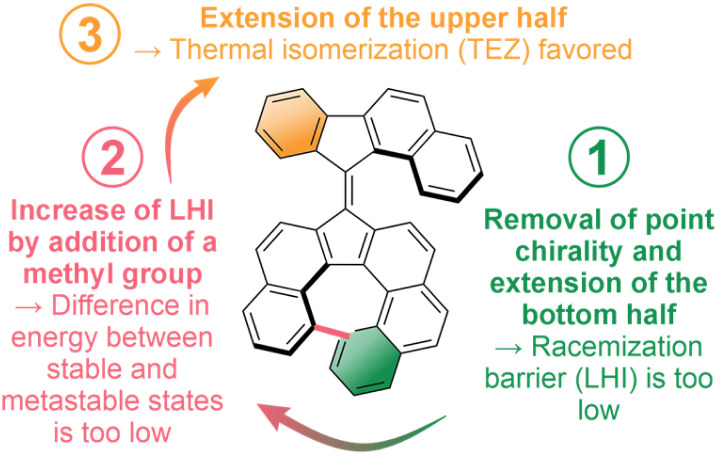
Summary of the design changes attempted, and their consequence on the properties of the obtained overcrowded alkenes.

In conclusion, we developed a series of unique overcrowded-alkenes as prototypes of molecular motors displaying only helical chirality, distinct from the previous generations of molecular motors which always display (pro-)chiral centers. We revealed the importance of a subtle balance between different isomerization processes in these inherently chiral photoactive systems. Nevertheless, despite theoretically evidencing some directionality bias which could potentially be harnessed to perform partial unidirectional rotative motion, our molecular designs could not efficiently perform complete continuous unidirectional rotation cycles. This lack in directionality was influenced by various factors such as low helix inversion barrier of the helicene fragment, a facile unproductive TEZ isomerization process, or, mainly, a small energy difference between stable and “metastable” isomers, hampering the ratcheting mechanism, which is crucial for the directional rotation of most overcrowded-alkene based molecular motors (Fig. 6).

By establishing the molecular design principles in this study, we expect that access to more widely applicable molecular motors, which harness helicity as the chiral source of unidirectional rotation, will be enabled in the near future.

## Author contributions

Conceptualization: YG, BLF. Funding acquisition: YG, ES, BLF. Investigation, methodology: YG, ES. Writing original draft, review and editing: YG, ES, BLF. Supervision: BLF.

## Conflicts of interest

There are no conflicts to declare.

## Supplementary Material

SC-017-D6SC00373G-s001

SC-017-D6SC00373G-s002

## Data Availability

The supporting data has been provided as part of the supplementary information (SI). Supplementary information: experimental procedures and details; detailed computational studies and calculated coordinates; CD and NMR spectra; HPLC chromatograms. See DOI: https://doi.org/10.1039/d6sc00373g.
