# Supplementary material for: Towards helical-chirality-controlled molecular motors
Source: Chem Sci. 2026 Apr 29;17(25):12377–84. doi: 10.1039/d6sc00373g (PMC13174676; doi:10.1039/d6sc00373g)
Supplement: SC-017-D6SC00373G-s001 [file SC-017-D6SC00373G-s001.pdf]

Supplementary Information (SI) for Chemical Science.
This journal is © The Royal Society of Chemistry 2026

Supporting Information

Towards Helical-Chirality-Controlled Molecular Motors

Yohan Gisbert,[1,2]* Eric Sidler,[1,3] Ben L. Feringa[1]*

1) Stratingh Institute for Chemistry, University of Groningen, Nijenborgh 3, 9747 AG Groningen (The Netherlands).
2) Current address: Univ Rennes, CNRS, ISCR (Institut des Sciences Chimiques de Rennes) − UMR 6226, F-35000 Rennes, France.
3) Current address: University of Zurich, Department of Chemistry, Winterthurerstrasse 190, 8057 Zürich, Switzerland.

Table of contents

1. Supplementary experimental procedures ______ ***2***

1.1. General methods ______ **2**

1.1. Computational methods ______ **3**

1.2. Synthetic procedures ______ **4**

2. In-situ NMR irradiation and relaxation experiments ______ ***17***

3. Chiral Resolution and CD spectra ______ ***26***

4. Enantiomerization Barrier ______ ***31***

5. Computational analysis ______ ***32***

6. NMR spectra of new compounds ______ ***38***

7. References ______ ***74***

1. Supplementary experimental procedures

1.1. General methods

Commercial reagents and solvents: All chemicals and solvents were purchased from commercial suppliers unless otherwise stated. Anhydrous solvents were obtained using a MBraun SPS 800 system and stored under N_2.

Synthesized reagents:

2,3-dihydrophenanthren-4(1*H*)-one[1], 2-methylene-1-oxo-1,2,3,4-tetrahydronaphthalene,[2] 8-methyl-1-tetralone[1], (2,3-dihydro-1*H*-cyclopenta[*a*]naphthalen-1-ylidene)hydrazone[3], 11-diazo-11H-benzo[a]fluorene[4] were synthesized according to reported literature procedures and characterized using routine characterization techniques.

Synthesis and purification: Standard Schlenk techniques were used, employing nitrogen or argon as the inert gas. If they were not performed at room temperature, the reaction temperatures refer to the temperature of the heating/cooling bath or heating block.

Flash column chromatography was performed on a Biotage Selekt system using the indicated solvents. TLC analysis was done on Merck silica gel 60 F_{254} aluminum sheets, and compounds were visualized with a UV lamp (254 nm or 365 nm).

Analysis:

NMR: Full characterization of the newly synthesized compounds (including ^{1}H, ^{13}C, ^{29}Si and 2D NMR experiments) was performed using a Bruker Avance Neo 600 (600 MHz) or a Agilent 400-MR (400 MHz) spectrometer. Chemical shifts (δ) are given in parts per million (ppm) relative to TMS, using the solvent residual peak as internal standard ($CDCl_3$: δ = 7.26 for ^{1}H, δ = 77.16 for ^{13}C; CD_2Cl_2: δ = 5.32 for ^{1}H, δ = 53.84 for ^{13}C). Multiplicity (s = singlet, d = doublet, dd = doublet of doublets, ddd = doublet of doublets of doublets (etc.), t = triplet, t_p = apparent/pseudo-triplet, q = quartet, br. = broad, m = multiplet), coupling constants *J* (Hz), and integration. Signals were assigned with the help of 2D NMR experiments. Variable temperature NMR and in-situ irradiation experiments were performed using a Varian Inova 500 (500 MHz) spectrometer. NMR irradiation experiments were performed at the indicated temperature with a fiber-coupled LED and a 1500 µm optical fiber (FT1500UMT) to guide the light directly into the NMR tube inside the NMR spectrometer.

High-resolution mass (HMRS) spectra were recorded on a Thermofisher LTQ Orbitrap XL.

HPLC analysis was performed using a Shimadzu Prominence System equipped with a diode array detector using CHIRALPAK columns (Daicel Corporation) with mixtures of HPLC-grade *n*-heptane and dichloromethane as the eluent and a column temperature of 40 to 70 °C. **CD** spectra were recorded on a Jasco J-815 spectropolarimeter.

Irradiation experiments were performed using fiber-coupled LEDs (M405L4, M455L4) powered with a T-Cube™ LEDD1B driver obtained from Thorlabs Inc.

1.1. Computational methods

All calculations were performed using the Orca 5.0.3 package.[5] Geometries were optimized with the composite method r^{2}SCAN-3c,[6] using the conductor-like polarizable continuum CPCM(CH_2Cl_2) solvent model.[7] The thermochemical data were calculated at the same level of theory. The minima and transition states had no or one imaginary frequency, respectively.

1.2. Synthetic procedures

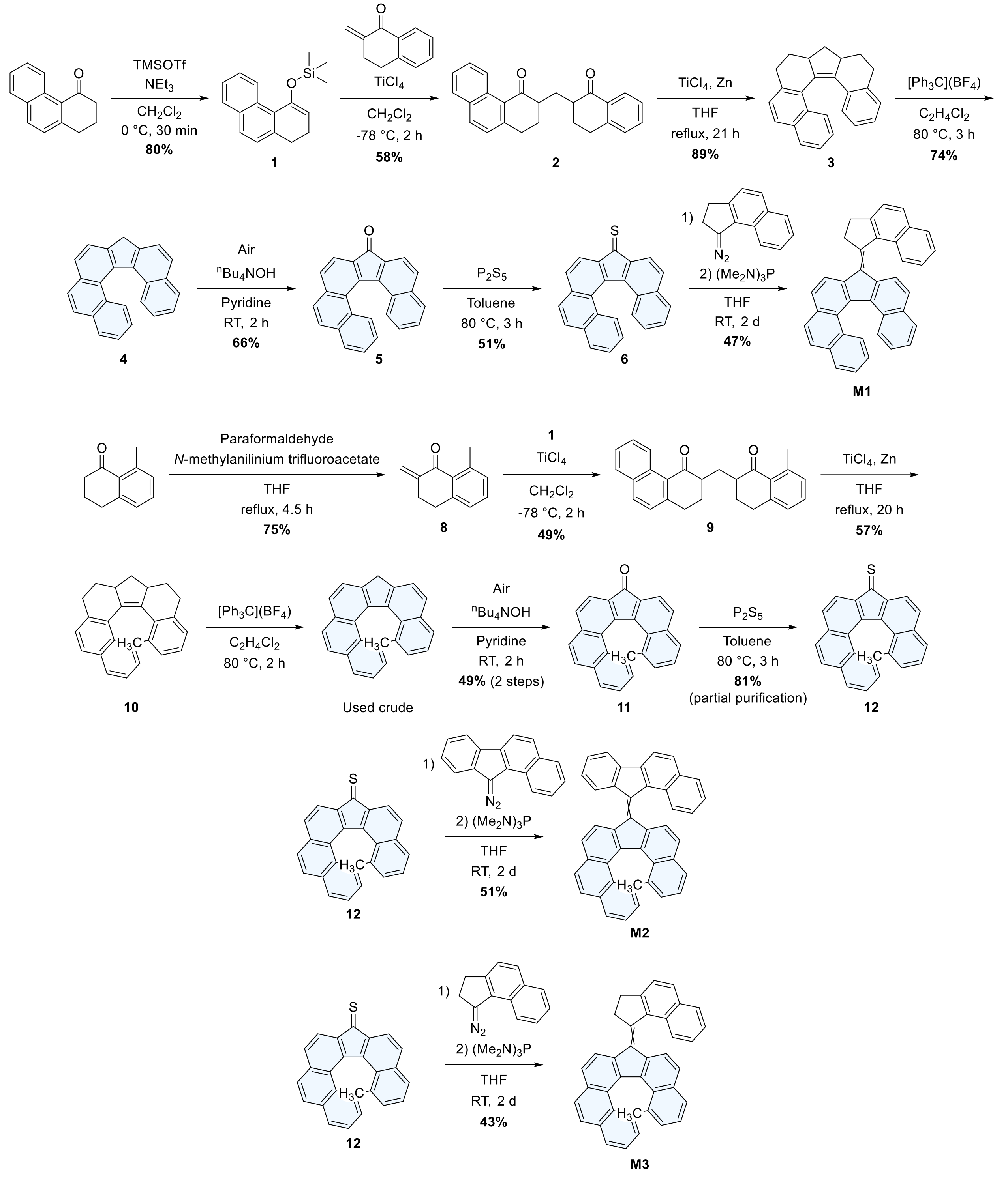

((1,2-Dihydrophenanthren-4-yl)oxy)trimethylsilane (**1**)

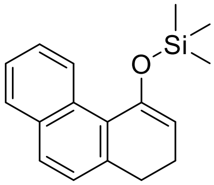

2,3-Dihydrophenanthren-4(1*H*)-one[1] (1.80 g, 9.17 mmol, 1 equiv.) was dissolved in anhydrous dichloromethane (13 mL) and the solution was cooled down to 0 °C under a nitrogen atmosphere. Then, triethylamine (1.4 mL, 10.08 mmol, 1.1 equiv.) and trimethylsilyl trifluoromethanesulfonate (1.7 mL, 9.17 mmol, 1.0 equiv.) were successively added. The resulting solution was stirred at this temperature for 30 min before being filtered over a short silica plug eluted with CH_2Cl_2/pentane 0:100 to 20:80. Solvents were removed to afford ((1,2-dihydrophenanthren-4-yl)oxy)trimethylsilane **1** as a turbid oil in 80% yield (1.96 g, 7.30 mmol). The obtained silyl enol ether was either used immediately or temporarily stored in a freezer at –20 °C and used within a few days.

***R*$_f$** = 0.37 (SiO_2, pentane). **^{1}H NMR** (600 MHz, $CDCl_3$, 25 °C): δ = 8.92 (d, *J* = 8.8 Hz, 1H), 7.76 (d, *J* = 8.9 Hz, 1H), 7.66 (d, *J* = 8.1 Hz, 1H), 7.40 (dddd, *J* = 20.2, 8.0, 6.7, 1.5 Hz, 2H), 7.30 (d, *J* = 8.2 Hz, 1H), 5.46 (t, *J* = 5.1 Hz, 1H), 2.87 – 2.80 (m, 2H), 2.27 – 2.22 (m, 2H), 0.19 (s, 9H) ppm. **^{13}C{^{1}H} NMR** (151 MHz, $CDCl_3$, 25 °C): δ = 150.8, 137.0, 133.7, 129.8, 129.3, 128.4, 128.0, 127.2, 126.2, 125.5, 124.7, 108.4, 30.7, 21.9, 0.1 ppm. **^{29}Si{^{1}H} NMR** (80 MHz, $CDCl_3$, 25 °C) : δ = 18.89 ppm. **HR-MS** (ESI+): calcd. for $C_{17}H_{21}OSi^+$ $[MH]^+$: 269.13562, found 269.13526.

1,5-Diketone **2**

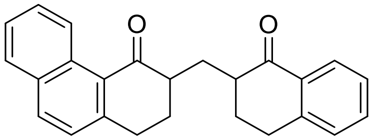

Degassed anhydrous dichloromethane (46 mL) was cooled down to –78 °C under an inert atmosphere. $TiCl_4$ (1.4 mL, 12.70 mmol, 1.1 equiv.) was slowly added. Then, a solution of 2-methylene-1-oxo-1,2,3,4-tetrahydronaphthalene[2] (1.83 g, 11.55 mmol, 1 equiv.) dissolved in 17 mL of anhydrous dichloromethane and a solution of ((1,2-dihydrophenanthren-4-yl)oxy)trimethylsilane **1** (3.10 g, 8.84 mmol, 1 equiv.) dissolved in 17 mL of dichloromethane were successively added. The reaction mixture was stirred at –78 °C for 2 h before being warmed-up to room temperature and the mixture quenched by addition of a saturated aqueous solution of potassium carbonate (30 mL). The layers were separated, and the aqueous fraction was washed with dichloromethane (2x30 mL). The combined organic layers were washed with brine (100 mL), dried over magnesium sulfate and the solvents were evaporated in vacuo. The crude product was then adsorbed on celite and purified by flash column chromatography (SiO_2, acetone/pentane 5:95) to afford 1,5-diketone **2** as a yellow oil in 58% yield (2.38 g, 6.72 mmol). The obtained product is composed of a mixture of all four possible diastereomers.

***R*f** = 0.43 (SiO_2, acetone/pentane 6:94). **^{1}H NMR** (600 MHz, $CDCl_3$, 25 °C): δ = 9.27 – 9.22 (m, 1H), 8.01 (d, J = 7.9 Hz, 1H), 7.90 (d, J = 8.4 Hz, 1H), 7.81 – 7.78 (m, 1H), 7.61 – 7.57 (m, 1H), 7.49 – 7.44 (m, 2H), 7.31 – 7.27 (m, 2H), 7.26 – 7.22 (m, 1H), 3.27 – 3.16 (m, 2H), 3.12 – 2.98 (m, 2H), 2.97 – 2.92 (m, 1H), 2.85 – 2.72 (m, 2H), 2.45 – 2.32 (m, 2H), 2.11 – 1.91 (m, 2H), 1.69 – 1.62 (m, 1H) ppm. **^{13}C{^{1}H} NMR** (151 MHz, $CDCl_3$, 25 °C): δ = 203.7, 203.3, 201.0, 200.4, 145.5, 144.1, 144.0, 134.0, 133.9, 133.3, 132.9, 132.9, 132.7, 132.6, 131.4, 131.4, 128.8, 128.7, 128.7, 128.4, 127.8, 127.7, 127.6, 127.5, 127.5, 127.0, 126.7, 126.5, 125.9, 47.4, 46.3, 46.0, 45.2, 31.3, 30.5, 30.1, 30.0, 29.5, 29.5, 29.2, 29.2, 28.7, 28.6 ppm. **HR-MS** (APCI+): calcd. for $C_{25}H_{22}O_2Na^+$ $[M+Na]^+$: 377.15120, found 377.15008.
Due to the presence of diastereoisomers featuring overlapping ^{13}C signals, the number of reported signals is lower than the expected number of carbon atoms.

Hydrogenated fluoreno[6]helicene **3**

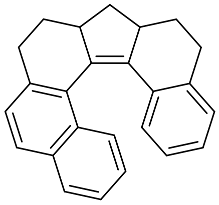

Zinc powder (4.24 g, 64.49 mmol, 10 equiv.) was stirred vigorously under an inert atmosphere for one hour in order to partially break the oxidation layer. Degassed anhydrous tetrahydrofuran (65 mL) was then added and the suspension was cooled to 0 °C before slowly adding $TiCl_4$ (2.9 mL, 25.96 mmol, 4 equiv.). The mixture was heated at reflux for 3 h before being allowed to cool down to room temperature. A solution of diketone **2** (2.30 g, 6.49 mmol, 1 equiv.) dissolved in tetrahydrofuran (15 mL) was then added dropwise over 2 h using a syringe pump. The reaction mixture was heated at reflux for 16 h. After cooling down to room temperature, the solvents were removed, and the residue redissolved in CH_2Cl_2 (100 mL). The reaction mixture was filtered over celite and washed with 100 mL of a 1M aqueous HCl solution, followed by water (100 mL) and brine (100 mL). The organic layer was then dried over magnesium sulfate and the solvents were removed in vacuo. The crude product was then purified by column chromatography (SiO_2, CH_2Cl_2/pentane 0:100 to 5:95) to afford the hydrogenated fluoreno[6]helicene **3** as a white solid in 89% yield (1.87 g, 5.80 mmol). The obtained product is composed of a mixture of all four possible diastereomers.

***R*f** = 0.39 (SiO_2, pentane). **^{1}H NMR** (600 MHz, $CDCl_3$, 25 °C): δ = 7.87 (d, *J* = 7.5 Hz), 7.80 – 7.76 (m), 7.70 (d, *J* = 8.4 Hz), 7.67 (d, *J* = 8.5 Hz), 7.43 (d, *J* = 8.2 Hz), 7.41 – 7.38 (m), 7.34 – 7.28 (m), 7.15 (d, *J* = 7.0 Hz), 7.12 (d, *J* = 7.1 Hz), 6.99 (t, *J* = 6.7 Hz), 6.96 (t, *J* = 7.4 Hz), 6.88 (d, *J* = 8.1 Hz), 6.67 (d, J = 7.9 Hz), 6.59 – 6.53 (m), 3.17 (m), 3.13 – 2.99 (m), 2.96 – 2.89 (m), 2.88 – 2.80 (m), 2.78 – 2.70 (m), 2.60 (m), 2.51 – 2.45 (m), 2.31 – 2.24 (m), 2.24 – 2.15 (m), 2.08 – 2.02 (m), 2.02 – 1.84 (m), 1.83 – 1.78 (m), 1.71 – 1.63 (m), 1.35 – 1.27 (m) ppm. **^{13}C{^{1}H} NMR** (151 MHz, $CDCl_3$, 25 °C): δ = 140.8, 139.1, 137.8, 136.8, 136.2, 134.2, 133.9, 133.9, 133.6, 133.3, 132.9, 132.4, 132.1, 132.1, 129.1, 128.8, 128.7, 128.6, 128.3, 128.0, 127.7, 127.5, 127.4, 127.4, 127.1, 127.0, 126.8, 126.6, 126.3, 126.1, 125.5, 125.4, 125.0, 124.9, 124.6, 47.7, 46.9, 46.2, 44.3, 40.4, 34.8, 32.3, 32.3, 31.2, 30.9, 30.2, 29.7, 29.5, 29.4 ppm. **HR-MS** (ESI+): calcd. for $C_{25}H_{23}^+$ $[M+H]^+$: 323.17943, found 323.17855.

Due to the presence of diastereoisomers featuring overlapping ^{13}C signals, one aromatic carbon signal is missing. Moreover, the ^{1}H NMR spectrum could not be accurately integrated, for the same reason.

Fluoreno[6]helicene **4**

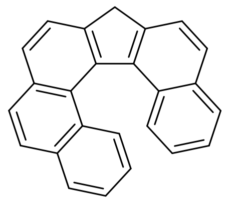

Hydrogenated fluoreno[6]helicene **3** (1.74 g, 5.38 mmol, 1 equiv.) was dissolved in anhydrous dichloroethane (135 mL). Tritylium tetrafluoroborate (8.00 g, 24.23 mmol, 4.5 equiv.) was then added under an inert atmosphere and the mixture was stirred at 80 °C for 3 h. The mixture was then cooled down to room temperature, filtered through neutral alumina (CH_2Cl_2) and the solvents were removed by rotary evaporation. The crude product was purified by column chromatography (SiO_2, CH_2Cl_2/Pentane 0:100 to 5:95) to afford fluoreno[6]helicene **4** as a white solid in a 74% yield (1.26 g, 3.97 mmol).

***R*f** = 0.25 (SiO_2, CH_2Cl_2/pentane 5:95). **^{1}H NMR** (600 MHz, $CDCl_3$, 25 °C): δ = 8.12 (d, *J* = 8.3 Hz, 1H), 7.93 (d, *J* = 8.2 Hz, 1H), 7.90 (d, *J* = 8.2 Hz, 2H), 7.85 – 7.77 (m, 5H), 7.54 (d, *J* = 8.7 Hz, 1H), 7.48 (t, *J* = 7.4 Hz, 1H), 7.38 (t, *J* = 7.4 Hz, 1H), 7.08 (t, *J* = 7.6 Hz, 1H), 7.04 (t, *J* = 7.6 Hz, 1H), 4.33 (d, J = 22.3 Hz, 1H), 4.14 (d, *J* = 22.3 Hz, 1H) ppm. **^{13}C{^{1}H} NMR** (151 MHz, $CDCl_3$, 25 °C): δ = 144.5, 143.1, 139.5, 137.0, 133.5, 132.7, 132.4, 130.3, 129.9, 128.8, 128.6, 128.3, 128.2, 127.5, 127.2, 127.1, 127.0, 126.5, 126.4, 124.9, 124.4, 124.3, 123.2, 122.8, 38.9 ppm. **HR-MS** (APCI+): calcd. for $C_{26}H_{16}^{+}$ $[M+H]^{+}$: 317.13248, found 317.13288.

Fluoreno[6]helicenone **5**

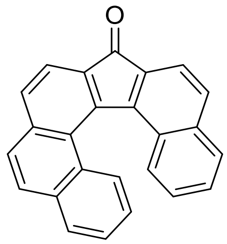

To a solution of fluoreno[6]helicene **4** (0.55 g, 1.74 mmol, 1 equiv.) was added pyridine (1.7 mL) in an oversized 25 mL round bottom flask equipped with the largest possible ovoid stir bar. A 1M solution of tetrabutylammonium hydroxide in methanol (45 µL, 0.05 mmol, 0.26 equiv.) was added, inducing a sudden color change to dark purple. The mixture was submitted to strong stirring at room temperature without closing the flask for 2 h. The pyridine was then evaporated and the residue was redissolved in dichloromethane (15 mL) and washed with a 0.1 M aqueous HCl solution (2x15 mL) followed by brine (15 mL). The organic layer was then dried over magnesium sulfate and the solvent was removed by rotary evaporation. The crude product was adsorbed onto celite and purified by column chromatography (SiO_2, CH_2Cl_2/pentane 10:90 to

20:80) to afford pure 9*H*-benzo[6,7]indeno[2,1-*c*]phenanthren-9-one **5** as a purple solid in 66% yield (0.38 g, 1.16 mmol).

***R*f** = 0.23 (SiO_2, CH_2Cl_2/pentane 20:80). **^{1}H NMR** (600 MHz, $CDCl_3$, 25 °C): δ = 8.11 (d, *J* = 8.2 Hz, 1H), 7.86 – 7.79 (m, 5H), 7.75 (d, *J* = 8.8 Hz, 1H), 7.72 (d, *J* = 7.5 Hz, 1H), 7.65 (d, *J* = 8.8 Hz, 1H), 7.50 (ddd, *J* = 8.0, 7.0, 1.1 Hz, 1H), 7.44 – 7.39 (m, 2H), 7.10 (ddd, *J* = 8.4, 7.0, 1.4 Hz, 1H), 7.02 (ddd, *J* = 8.4, 6.8, 1.3 Hz, 1H) ppm. **^{13}C{^{1}H} NMR** (151 MHz, $CDCl_3$, 25 °C): δ = 194.0, 148.7, 144.4, 138.8, 138.4, 135.8, 133.2, 133.1, 130.5, 130.4, 130.3, 129.5, 129.4, 129.3, 129.1, 128.6, 128.2, 127.8, 127.8, 127.6, 127.1, 125.9, 124.7, 120.6, 120.2 ppm. **HR-MS** (ESI+): calcd. for $C_{26}H_{15}O^+$ $[M+H]^+$: 331.11174, found 331.11125.

Thioketone **6**

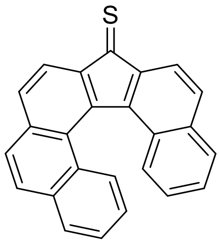

To a solution of **5** (60 mg, 181 µmol, 1 equiv.) in anhydrous toluene (1.8 mL) under a N_2 atmosphere was added P_2S_5 (404 mg, 1.82 mmol, 10 equiv.). The suspension was heated to 80 °C under vigorous stirring for three hours. The mixture was allowed to cool down to room temperature, before being directly purified using a short chromatographic column (SiO_2, pentane/CH_2Cl_2 100:0 to 50:50) to yield pure thioketone **6** as a dark brown solid in a 51% yield (32 mg, 92 µmol).

***R*f** = 0.46 (SiO_2, CH_2Cl_2/pentane 20:80). **^{1}H NMR** (600 MHz, $CDCl_3$, 25 °C): δ = 8.15 (d, *J* = 8.3 Hz, 1H), 7.93 (d, *J* = 8.3 Hz, 1H), 7.90 (d, *J* = 7.7 Hz, 1H), 7.81 (d, *J* = 7.8 Hz, 1H), 7.75 (d, *J* = 7.9 Hz, 1H), 7.73 (d, *J* = 8.6 Hz, 1H), 7.71 (d, *J* = 8.3 Hz, 1H), 7.60 (t_p, *J* = 7.7 Hz, 2H), 7.48 (t_p, *J* = 7.4 Hz, 1H), 7.43 (d, *J* = 8.6 Hz, 1H), 7.38 (t_p, *J* = 7.5 Hz, 1H), 7.10 (t, *J* = 7.6 Hz, 1H), 7.00 – 6.96 (m, 1H) ppm. **^{13}C{^{1}H} NMR** (151 MHz, $CDCl_3$, 25 °C): δ = 147.6, 143.9, 141.2, 138.5, 138.5, 138.1, 133.4, 130.7, 130.6, 130.0, 129.9, 129.5, 129.1, 129.0, 128.2, 128.1, 127.7, 127.6, 127.4, 127.3, 126.0, 124.6, 121.1, 120.7 ppm. **HR-MS** (APCI+): calcd. for $C_{25}H_{15}S^+$ $[M+H]^+$: 347.08890, found 347.08893.
^{13}C NMR signal for C_{quat}=S was not observed in the recorded spectra (up to 220 ppm) and is probably downfield from this value.

M1

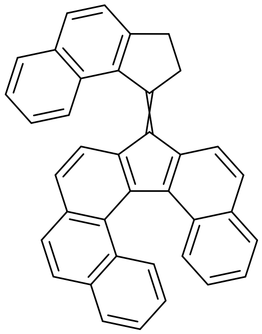

(2,3-dihydro-1*H*-cyclopenta[*a*]naphthalen-1-ylidene)hydrazone[3] (19 mg, 98 µmol, 2.0 equiv.) was dissolved in anhydrous tetrahydrofuran (1.3 mL) and the suspension was degassed by bubbling argon for 3 min. The mixture was cooled down to 0 °C and placed in the dark. MnO_2 (26 mg, 0.29 mmol, 6 equiv.) was added and the mixture was stirred at 0 °C for 10 min. The *in-situ* generated diazo compound was then filtered and transferred to another Schlenk flask containing thioketone **6** (17 mg, 49 µmol, 1 equiv.) by cannula filtration. An extra 1 mL of THF was used for rinsing. The resulting solution was then stirred at room temperature for 24 h in the dark before tris(dimethylamino)phosphine (25 µL, 0.15 mmol, 3 equiv.) was added. The reaction mixture was stirred for a further 16 h and the volatiles were removed in vacuo. The crude product was purified by column chromatography (SiO_2, CH_2Cl_2/pentane, 10:90) to yield pure **M1** as a mixture of stereoisomers, as an orange solid in 47% yield (11 mg, 23 µmol) as a ca. 1:1 mixture of the *Z* and *E* isomers.

Analytical samples of pure or highly enriched *Z* and *E* isomers were obtained by column chromatography (SiO_2, Et_2O/pentane 3:97). In order to obtain enrichment, the column has to be long (approx. 40 cm x 1 cm for 5-10 mg of sample) and protected from direct light. Only the head and tail of the product were collected and studied.

$\boldsymbol{R_f}$ = 0.41 (SiO_2, CH_2Cl_2/pentane 10:90). **^{1}H NMR** (600 MHz, $CDCl_3$, 25 °C): δ = 8.41 (d, *J* = 8.3 Hz), 8.37 (d, *J* = 8.4 Hz), 8.35 – 8.28 (m), 8.26 – 8.15 (m), 8.01 – 7.96 (m), 7.94 – 7.79 (m), 7.75 – 7.62 (m), 7.58 (d, *J* = 8.6 Hz), 7.56 – 7.40 (m), 7.40 – 7.32 (m), 7.22 – 7.11 (m), 7.09 – 6.93 (m), 4.16 – 4.00 (m), 3.90 – 3.75 (m), 3.57 – 3.45 (m), 3.40 – 3.25 (m) ppm. **^{13}C{^{1}H} NMR** (151 MHz, $CDCl_3$, 25 °C): δ = 151.0, 150.4, 143.4, 138.7, 137.8, 137.5, 136.5, 133.6, 133.5, 133.2, 133.1, 132.8, 132.5, 132.0, 131.8, 130.6, 129.9, 129.8, 129.7, 129.1, 128.9, 128.9, 128.72, 128.2, 128.2, 128.0, 127.8, 127.8, 127.6, 127.5, 127.4, 127.4, 127.3, 127.1, 127.0, 126.8, 126.8, 126.6, 126.3, 125.9, 125.9, 125.8, 125.3, 125.1, 125.1, 124.8, 124.5, 124.4, 124.1, 123.6, 123.6, 123.5, 123.4, 122.6, 122.4, 121.7, 42.0, 40.7, 40.6, 34.2, 34.0, 33.9, 31.1 ppm. **HR-MS** (ESI+): calcd. for $C_{38}H_{25}^+$ $[M+H]^+$: 481.19508, found 481.19365.
Due to the presence of all the possible stereoisomers in varying ratios, ^{1}H NMR could not be integrated accurately.

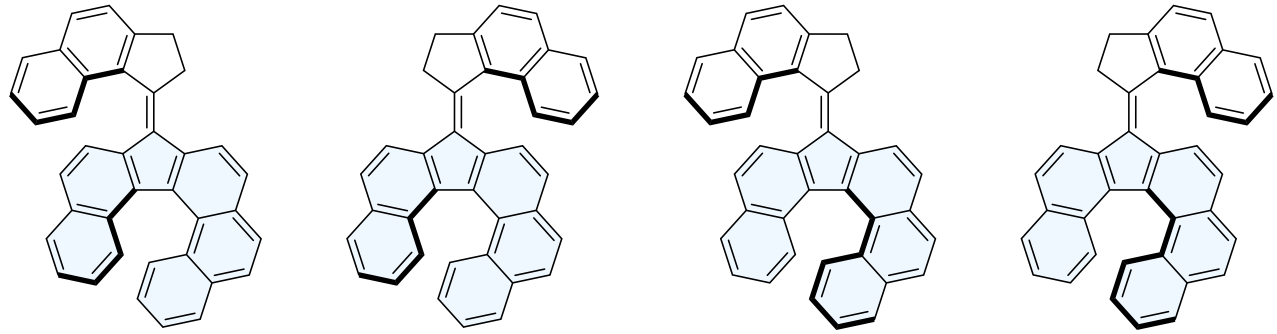

(P,P)-E-**M1** (M,P)-Z-**M1** (P,M)-E-**M1** (M,M)-Z-**M1**

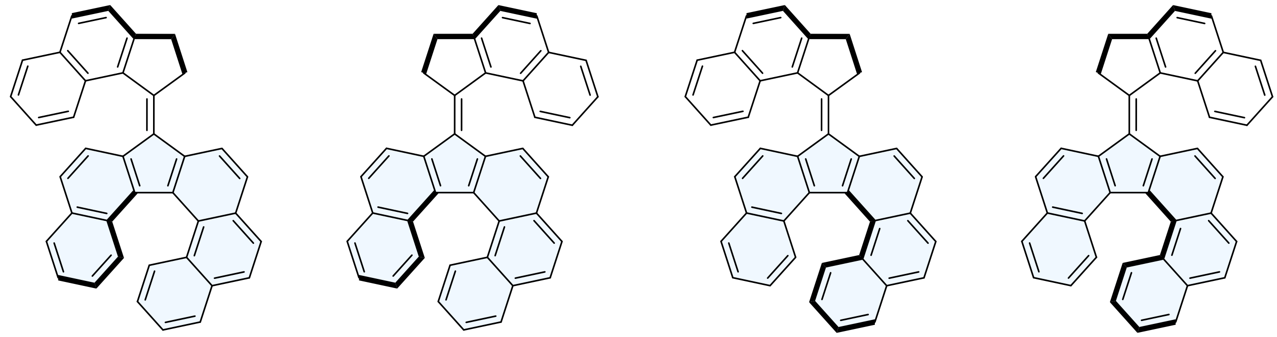

(M,P)-E-**M1** (P,P)-Z-**M1** (M,M)-E-**M1** (P,M)-Z-**M1**

Figure S1. The eight stereoisomers of **M1**.

10-Fluoro-11*H*-benzo[*a*]fluoren-11-one **7**

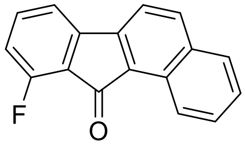

10-Fluoro-11*H*-benzo[*a*]fluoren-11-one **7** was synthesized according to a method described by Lautens et al.[8] for other substituted benzofluorenones.

2-Bromo-6-fluorobenzaldehyde (1.20 g, 5.9 mmol, 1.5 equiv.), 1-iodonaphthalene (1 g, 3.94 mmol, 1 equiv.), palladium(II) acetate (88 mg, 0.39 mmol, 0.1 equiv.), triphenylphosphine (0.206 g, 0.79 mmol, 0.2 equiv.), cesium carbonate (3.85 g, 11.8 mmol, 3 equiv.) and norbornene (1.11 g, 11.8 mmol, 3 equiv.) were placed in a dry Schlenk tube which was then filed with argon. Anhydrous dimethoxyethane (80 mL) was added and the mixture was degassed by bubbling argon for 15 min under stirring. The Schlenk tube was sealed and heated to 90 °C for 24 h. After cooling down to room temperature, the crude mixture was filtered through SiO_2 (eluted with CH_2Cl_2). The solvents were removed and the residue was redissolved in CH_2Cl_2. Heptane (20 mL) was added, and the CH_2Cl_2 was removed by rotary evaporation, inducing precipitation of the product. The suspension was then cooled with an ice bath, the precipitate was filtered out, rinsed with a small amount of ice-cold pentane and dried under vacuum to yield pure 10-fluoro-11*H*-benzo[*a*]fluoren-11-one **7** in a 95% yield (0.926 g, 3.73 mmol).

***R*$_f$** = 0.22 (SiO_2, CH_2Cl_2/pentane 20:80). **^{1}H NMR** (600 MHz, $CDCl_3$, 25 °C): δ = 8.96 (d, *J* = 8.4 Hz, 1H), 8.00 (d, *J* = 8.2 Hz, 1H), 7.79 (d, *J* = 8.3 Hz, 1H), 7.64 (d, *J* = 8.3 Hz, 1H), 7.62 – 7.59 (m, 1H), 7.48 – 7.44 (m, 1H), 7.42 (ddd, *J* = 8.2, 7.2, 4.8 Hz, 1H), 7.29 (d, *J* = 7.2 Hz, 1H), 6.92 (t_p, *J* = 8.6 Hz, 1H) ppm. **{^{1}H} NMR** (151 MHz, $CDCl_3$, 25 °C): δ = 191.8, 160.0, 158.2, 146.0 (d, J = 3.7 Hz), 145.1 (d, J = 3.8 Hz), 136.7 (d, J = 8.3 Hz), 136.0, 134.8, 130.2, 129.8, 128.6, 126.9, 124.4, 120.0 (d, J = 12.4 Hz), 118.2 (d, *J* = 21.2 Hz), 118.2, 116.3 (d, J = 2.8 Hz) ppm. **^{19}F NMR** (565 MHz, $CDCl_3$, 25 °C) δ -114.70 (dd, J = 8.9, 4.9 Hz) ppm. **^{13}C HR-MS** (APCI+): calcd. for $C_{17}H_{10}FO^+$ $[M+H]^+$: 250.07437, found 250.07361.

8-Methyl-2-methylene-1-tetralone (**8**)

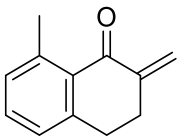

8-Methyl-1-tetralone[1] (2.48 g, 15.48 mmol, 1 equiv.), paraformaldehyde (2.09 g, 69.66 mmol, 4.5 equiv.) and *N*-methylanilinium trifluoroacetate (5.13 g, 23.22 mmol, 1.5 equiv.) was dispersed in anhydrous THF (16 mL) under a nitrogen atmosphere. The resulting suspension was stirred while heated at reflux for 4h30 min. The mixture was allowed to cool down to room temperature and poured in diethyl ether (50 mL) under vigorous stirring resulting in the precipitation of white particles and a red gum. The precipitate was filtered off and the solution was further washed with saturated aq. $NaHCO_3$ (75 mL) and brine (75 mL), dried over anhydrous magnesium sulfate and the solvents were removed in vacuo. The crude residue was purified by a short column chromatography (SiO_2, CH_2Cl_2/pentane 2:8) to afford pure 8-methyl-2-methylene-1-tetralone in

75% yield (1.99 g, 11.56 mmol) as a transparent oil. The obtained 8-methyl-2-methylene-1-tetralone was either used immediately or temporarily stored in a freezer at –20 °C and used within a few days. Decomposition (hetero-Diels-Alder) was observed upon prolongated rotary evaporation. Evaporation was thus performed with a water bath at room temperature as quickly as possible and column chromatography was performed on a short (3-4 cm) stationary phase to minimize the resulting volume of solvent and decomposition on the column.
This procedure is based on a previously reported protocol for the methylenation of 1-tetralone.[2]

***R*f** = 0.28 (SiO_2, CH_2Cl_2/pentane 10:90). **^{1}H NMR** (400 MHz, $CDCl_3$, 25 °C): δ = 7.31 (t, *J* = 7.6 Hz, 1H), 7.13 (d, *J* = 7.6 Hz, 1H), 7.09 (d, *J* = 7.6 Hz, 1H), 6.26 – 6.20 (m, 1H), 5.40 (d, *J* = 1.8 Hz, 1H), 2.98 – 2.91 (m, 2H), 2.82 – 2.77 (m, 2H), 2.68 (s, 3H) ppm. **^{13}C{^{1}H} NMR** (101 MHz, $CDCl_3$, 25 °C): δ = 188.9, 145.4, 144.5, 142.3, 132.4, 131.7, 130.9, 126.5, 121.3, 31.3, 30.6, 23.2 ppm. **HR-MS** (ESI+): calcd. for $C_{12}H_{13}O^+$ $[MH]^+$: 173.09609, found 173.09603.

1,5-diketone **9**

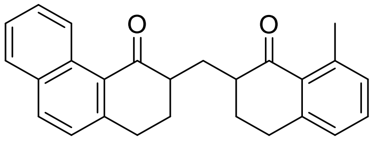

Degassed anhydrous dichloromethane (28 mL) was cooled down to –78 °C under an inert atmosphere. $TiCl_4$ (0.84 mL, 7.66 mmol, 1.1 equiv.) was slowly added. Then, a solution of 8-methyl-2-methylene-1-tetralone **8** (1.20 g, 6.97 mmol, 1 equiv.) dissolved in 10 mL of anhydrous dichloromethane and a solution of ((1,2-dihydrophenanthren-4-yl)oxy)trimethylsilane **1** (1.87 g, 6.97 mmol, 1 equiv.) dissolved in 12 mL of dichloromethane were successively added. The reaction mixture was stirred at –78 °C for 2 h before being warmed-up to room temperature and the mixture quenched by addition of a saturated aqueous solution of potassium carbonate (10 mL). The layers were separated, and the aqueous fraction was washed with dichloromethane (2x10 mL). The combined organic layers were washed with brine (50 mL), dried over magnesium sulfate and the solvents were evaporated in vacuo. The crude product was then adsorbed on celite and purified by flash column chromatography (SiO_2, acetone/pentane 97:3) to afford 1,5-diketone **9** as a transparent oil in 49% yield (1.25 g, 3.39 mmol). The obtained product is composed of a mixture of all four possible diastereomers.

***R*f** = 0.30 (SiO_2, CH_2Cl_2/pentane 30:70). **^{1}H NMR** (600 MHz, $CDCl_3$, 25 °C): δ = 9.26 – 9.21 (m, 1H), 7.93 – 7.89 (m, 1H), 7.84 – 7.76 (m, 1H), 7.63 – 7.56 (m, 1H), 7.51 – 7.45 (m, 1H), 7.31 – 7.28 (m, 2H), 7.08 (t, J = 8.3 Hz, 2H), 3.26 – 3.20 (m, 2H), 3.12 – 3.02 (m, 2H), 2.98 – 2.90 (m, 1H), 2.85 – 2.77 (m, 1H), 2.74 – 2.65 (m, 0.5H), 2.63 – 2.57 (m, 3H), 2.45 – 2.34 (m, 1H), 2.35 – 2.26 (m, 1H), 2.11 – 2.00 (m, 2.5H), 1.97 – 1.90 (m, 1H) ppm. **^{13}C{^{1}H} NMR** (151 MHz, $CDCl_3$, 25 °C): δ = 203.7, 203.3, 203.1, 202.7, 145.5, 145.5, 145.0, 145.0, 141.3, 141.2, 134.0, 133.9, 132.9, 132.9, 132.1, 132.1, 132.0, 131.6, 131.5, 131.5, 131.4, 130.5, 130.4, 128.9, 128.8, 128.7, 128.7, 128.5, 128.4, 127.9, 127.7, 127.0, 126.9, 126.5, 126.5, 125.9, 47.4, 47.4, 46.6, 46.4, 31.7, 30.9, 30.1, 29.9, 29.7, 29.6, 29.5, 29.4, 29.2, 29.0, 23.3, 23.2 ppm. **HR-MS** (ESI+): calcd. for $C_{26}H_{26}O_2^+$ $[M+H]^+$: 369.18491, found 369.18424.

As most ^{1}H NMR signals for the two sets of diastereoisomers of **9** overlap, the sum of the integrals was set as 24 (number of ^{1}H in **9**). Nevertheless, a few of the signals in the aliphatic region don't overlap, resulting in the integrals described with decimal number (0.5 and 2.5 respectively).

Hydrogenated methylated fluoreno[6]helicene **10**

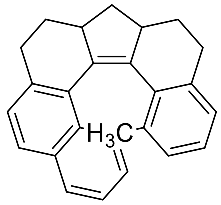

Zinc powder (2.22 g, 33.92 mmol, 10 equiv.) was stirred vigorously under an inert atmosphere for one hour in order to partially break the oxidation layer. Degassed anhydrous tetrahydrofuran (34 mL) was then added and the suspension was cooled to 0 °C before slowly adding $TiCl_4$ (1.5 mL, 13.57 mmol, 4 equiv.). The mixture was then heated at reflux for 3 h before being allowed to cool down to room temperature. A solution of diketone **9** (1.25 g, 3.39 mmol, 1 equiv.) dissolved in tetrahydrofuran (6 mL) was then added dropwise over 2 h using a syringe pump. The reaction mixture was then heated at reflux for 16 h. After cooling down to room temperature, the solvents were removed, the residue redissolved in CH_2Cl_2 (40 mL). The reaction mixture was filtered over celite and washed with 40 mL of a 1M aqueous HCl solution, followed by water (40mL) and brine (40 mL). The organic layer was then dried over magnesium sulfate and the solvents were removed in vacuo. The crude product was then purified by column chromatography (SiO_2, CH_2Cl_2/pentane 5:95 to 10:90) to afford the hydrogenated methylated fluoreno[6]helicene **10** as a white solid in 57% yield (0.65 g, 1.93 mmol). The obtained product is composed of a mixture of all four possible diastereomers.

$\boldsymbol{R_f}$ = 0.74 (SiO_2, pentane). **^{1}H NMR** (600 MHz, $CDCl_3$, 25 °C): δ 7.68 (d, *J* = 8.2 Hz, 2H), 7.62 (d, *J* = 8.4 Hz, 2H), 7.32 (d, *J* = 8.2 Hz, 2H), 7.18 (t, *J* = 7.0 Hz, 2H), 7.12 (d, *J* = 7.4 Hz, 2H), 7.09 – 7.06 (m, 2H), 7.05 – 7.01 (m, 2H), 6.73 – 6.64 (m, 4H), 3.12 – 3.05 (m, 4H), 3.00 – 2.91 (m, 4H), 2.91 – 2.83 (m, 4H), 2.76 – 2.69 (m, 2H), 2.44 – 2.37 (m, 2H), 2.15 – 2.07 (m, 2H), 1.99 – 1.89 (m, 2H), 1.74 (br. s, 2H), 1.47 (q, *J* = 11.2 Hz, 2H), 1.10 (s, 6H) ppm. **^{13}C{^{1}H} NMR** (151 MHz, $CDCl_3$, 25 °C): δ = 140.8, 139.2, 138.8, 137.2, 136.9, 136.7, 135.9, 135.3, 135.0, 133.5, 132.3, 131.9, 130.3, 129.6, 129.4, 128.5, 128.3, 127.9, 127.6, 127.5, 127.3, 127.2, 127.1, 126.7, 126.7, 126.6, 125.9, 125.7, 125.4, 125.0, 124.8, 124.7, 124.6, 124.4, 51.0, 49.2, 46.8, 46.1, 40.1, 36.9, 33.8, 33.3, 32.3, 31.9, 31.1, 30.6, 30.2, 29.9, 28.6, 27.7, 20.1, 19.7 ppm. **HR-MS** (APCI+): calcd. for $C_{26}H_{25}^{+}$ $[M+H]^{+}$: 337.19508, found 337.19492.

Methylated fluoreno[6]helicenone **11**

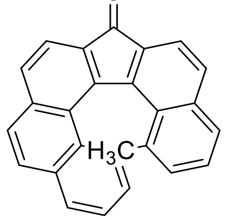

Hydrogenated methylated fluoreno[6]helicene **10** (0.30 g, 0.89 mmol, 1 equiv.) was dissolved in anhydrous dichloroethane (22 mL). Tritylium tetrafluoroborate (1.33 g, 4.01 mmol, 4.5 equiv.) was then added under an inert atmosphere and the mixture was stirred at 80 °C for 2 h. The mixture was then cooled down to room temperature, filtered through SiO_2 and neutral alumina (CH_2Cl_2) and the solvents were removed by rotary evaporation. The crude product was then dissolved in pyridine (4.5 mL) in an oversized 50 mL round bottom flask equipped with the largest possible ovoid stir bar. A 1M solution of tetrabutylammonium hydroxide in methanol (47 µL, 0.05 mmol, 0.05 equiv.) was added, inducing a sudden color change to dark purple. The mixture was submitted to strong stirring at room temperature without closing the flask for 2 h. The pyridine was then evaporated and the residue was redissolved in dichloromethane (20 mL) and washed with a 0.1 M aqueous HCl solution (2x20 mL) followed by brine (20 mL). The organic layer was then dried over magnesium sulfate and the solvent was removed by rotary evaporation. The crude product was adsorbed onto celite and purified by column chromatography (SiO_2, CH_2Cl_2/pentane 7:3) to afford pure methylated fluoreno[6]helicenone **11** as a purple solid in 49% yield over two steps (150 mg, 0.43 mmol).

Note: it was chosen to perform both reactions without intermediate purification because the fluorene derivative resulting from the first oxidation was found to be extremely difficult to separate from the excess triphenylmethane side-product, yielding low and irreproducible yields of a partially purified product when the intermediate purification was attempted.

$\boldsymbol{R_f}$ = 0.23 (SiO_2, CH_2Cl_2/pentane 20:80). **^{1}H NMR** (600 MHz, $CDCl_3$, 25 °C): δ = 7.84 (d, J = 8.3 Hz, 1H), 7.78 (d, J = 7.4 Hz, 1H), 7.76 – 7.71 (m, 3H), 7.65 (dd, J = 10.4, 8.1 Hz, 3H), 7.56 (d, J = 8.8 Hz, 1H), 7.43 (t_p, J = 7.4 Hz, 1H), 7.34 (t_p, J = 7.5 Hz, 1H), 6.92 – 6.87 (m, 2H), 1.64 (s, 3H) ppm. **^{13}C{^{1}H} NMR** (151 MHz, $CDCl_3$, 25 °C): δ 193.4, 149.4, 148.3, 138.9, 138.2, 136.7, 135.1, 134.1, 132.2, 131.2, 130.9, 130.9, 129.7, 129.7, 129.5, 129.2, 128.0, 127.7, 127.5, 127.2, 127.1, 127.0, 125.1, 120.5, 118.8, 22.6 ppm. **HR-MS** (ESI+): calcd. for $C_{26}H_{17}O^+$ $[M+H]^+$: 345.12739, found 345.12734.

Thioketone **12**

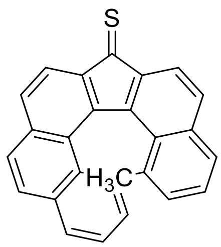

To a solution of **11** (20 mg, 58 µmol, 1 equiv.) in anhydrous toluene (2 mL) under a N_2 atmosphere was added P_2S_5 (129 mg, 580 µmol, 10 equiv.). The suspension was heated to 80 °C under vigorous stirring for three hours. The mixture was allowed to cool down to room temperature, before being directly purified using a short chromatographic column (SiO_2, CH_2Cl_2) to yield partially purified thioketone **12** as a dark brown solid in a 81% yield (17 mg, 47 µmol). This partially purified compound was used directly for the next step.

M2

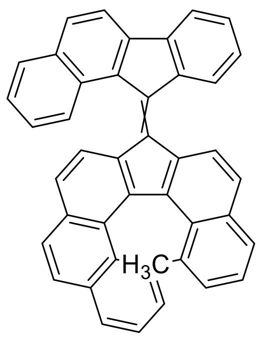

11-Diazo-11H-benzo[a]fluorene[4] (15 mg, 62 µmol, 1.5 equiv.) and thioketone **11** (15 mg, 42 µmol, 1 equiv.) were dissolved in anhydrous tetrahydrofuran (2 mL) under a nitrogen atmosphere. The reaction mixture was protected from light and stirred at room temperature for 24h. Tris(dimethylamino)phosphine (22 µL, 0.12 mmol, 3 equiv.) was added and the mixture was stirred for a further 16 h. The volatiles were then removed in vacuo. The crude product was adsorbed on silica and purified by column chromatography (SiO_2, CH_2Cl_2/pentane, 5:95) to yield pure **M2** as dark purple solid in 51% yield (11 mg, 21 µmol) as a ca. 1:1 mixture of the *Z* and *E* isomers featuring both P and M helicities of the lower and upper halves, for a total of 8 stereoisomers (see Figure S1).
One of the two isomers could be highly enriched by slow precipitation from a concentrated CH_2Cl_2 solution layered with MeOH.

R_f = 0.63 (SiO_2, CH_2Cl_2/pentane 10:90). **^{1}H NMR** (600 MHz, CD_2Cl_2, 25 °C): δ = 8.62 – 8.57 (m), 8.53 – 8.47 (m), 8.39 – 8.30 (m), 8.19 (d, J = 8.0 Hz), 8.15 – 8.06 (m), 8.03 – 7.97 (m), 7.95 – 7.91 (m), 7.88 – 7.84 (m), 7.84 – 7.69 (m), 7.66 – 7.61 (m), 7.57 – 7.51 (m), 7.47 – 7.22 (m), 7.11 – 7.06 (m), 7.06 – 7.01 (m), 6.96 – 6.82 (m), 1.72 (s), 1.69 (s), 1.57 (s), 1.53 (s) ppm. **^{13}C{^{1}H} NMR** (101 MHz, CD_2Cl_2, 25 °C): δ = 145.0, 144.2, 143.4, 142.8, 142.5, 142.0, 141.4, 140.8, 140.7, 140.2, 140.1, 137.2, 137.0, 135.9, 135.3, 134.8, 134.5, 133.8, 132.9, 132.7, 132.6, 132.3, 132.1, 132.0, 131.8, 130.6, 129.9, 129.8, 129.3, 129.2, 129.1, 129.0, 128.9, 128.4, 128.2, 128.2, 128.0, 127.8, 127.7, 127.5, 127.3, 127.2, 127.1, 127.0, 127.0, 126.9, 126.7, 126.7, 126.6, 126.5, 125.8, 125.4, 125.2, 124.9, 124.5, 124.1, 120.3, 119.1, 23.0, 22.9 ppm. **HR-MS** (ESI-): calcd. for $C_{43}H_{26}^-$ $[M]^-$: 542.20258, found 542.20400.
Due to the presence of all the possible stereoisomers in varying ratios, ^{1}H NMR could not be integrated accurately.

M3

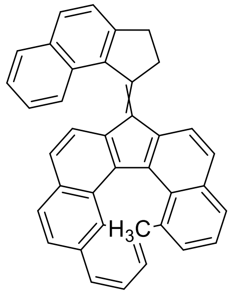

(2,3-dihydro-1*H*-cyclopenta[*a*]naphthalen-1-ylidene)hydrazone[3] (25 mg, 0.56 mmol, 1.2 equiv.) was dissolved in anhydrous tetrahydrofuran (3 mL). Anhydrous sodium sulfate (89 mg, 0.62 mmol, 9 equiv.) was added and the suspension was degassed by bubbling argon for 3 min. The reaction mixture was cooled down to 0 °C and placed in the dark. MnO_2 (36 mg, 0.42 mmol, 6 equiv.) was added and the suspension was stirred at 0 °C for 10 min. The *in-situ* generated diazo compound was then filtered and transferred to another Schlenk flask containing thioketone **11** (25 mg, 69 µmol, 1 equiv.) by cannula filtration. An extra 1 mL of THF was used for rinsing. The resulting solution was then stirred at room temperature for 24 h in the dark before tris(dimethylamino)phosphine (37 µL, 0.21 mmol, 3 equiv.) was added. The reaction mixture was stirred for a further 16 h and the volatiles were removed in vacuo. The crude product was adsorbed on silica and purified by column chromatography (SiO_2, CH_2Cl_2/pentane, 0:100 to 15:85) to yield pure **M3** as an orange solid in 43% yield (15 mg, 30 µmol) as a ca. 1:1 mixture of the *Z* and *E* isomers featuring both P and M helicities of the lower and upper halves, for a total of 8 stereoisomers (see Figure S1).
Analytical samples of pure or highly enriched *Z* and *E* isomers were obtained by column chromatography (SiO_2, Et_2O/pentane 3:97). In order to obtain enrichment, the column has to be long (approx. 40 cm x 1 cm for ca. 10 mg of sample) and protected from direct light. Only the head and tail of the product were collected and studied.

***R*$_f$** = 0.44 (SiO_2, CH_2Cl_2/pentane 10:90). **^{1}H NMR** (600 MHz, $CDCl_3$, 25 °C): δ = 8.21 (d, *J* = 8.0 Hz), 8.19 – 8.13 (m), 8.12 (d, *J* = 8.6 Hz), 8.08 (d, *J* = 8.3 Hz), 8.01 – 7.96 (m), 7.94 (d, *J* = 8.3 Hz), 7.89 – 7.74 (m), 7.74 – 7.68 (m), 7.67 – 7.56 (m), 7.56 – 7.51 (m), 7.48 – 7.23 (m), 7.22 – 7.13 (m), 7.09 (d, J = 8.5 Hz, 1H), 7.06 (d, J = 8.3 Hz, 0H), 6.99 (d, J = 8.4 Hz, 1H), 6.96 – 6.89 (m), 6.85 (d, *J* = 6.7 Hz), 6.84 – 6.76 (m), 4.15 – 3.97 (m), 3.88 – 3.79 (m), 3.56 – 3.45 (m), 3.40 – 3.25 (m), 1.71 (s), 1.61 (s), 1.56 (s), 1.47 (s) ppm. **^{13}C{^{1}H} NMR** (151 MHz, $CDCl_3$, 25 °C): δ = 151.2, 151.1, 151.0, 150.9, 150.4, 150.3, 149.7, 149.6, 142.2, 141.5, 139.2, 138.9, 138.8, 138.5, 138.2, 138.0, 137.9, 137.9, 137.9, 137.9, 137.7, 137.4, 137.4, 137.0, 136.4, 136.3, 135.9, 135.2, 134.4, 133.8, 133.7, 133.7, 133.7, 133.5, 133.4, 133.0, 133.0, 132.9, 132.0, 132.0, 132.0, 132.0, 131.9, 131.9, 131.8, 131.7, 131.7, 131.6, 131.5, 131.4, 131.4, 131.3, 131.3, 131.1, 130.8, 130.6, 130.6, 129.8, 129.8, 129.7, 129.5, 129.0, 128.9, 128.9, 128.7, 128.7, 128.6, 128.5, 128.3, 128.3, 128.2, 128.2, 128.0, 127.9, 127.8, 127.4, 127.4, 127.3, 127.3, 127.3, 127.3, 127.2, 127.2, 127.1, 127.1, 127.0, 127.0, 127.0, 127.0, 126.9, 126.8, 126.7, 126.6, 126.6, 126.5, 126.4, 126.4, 126.3, 126.3, 126.2, 126.2, 126.1, 126.1, 125.9, 125.8, 125.8, 125.8, 125.8, 125.7, 125.5, 125.4, 125.2, 125.1, 125.0, 124.6, 124.6, 124.4, 124.3, 123.5, 123.4, 123.4, 123.4, 122.9, 122.8, 122.5, 122.4, 121.8, 121.5, 121.0, 41.7, 41.4, 41.3, 41.3, 34.3, 34.1, 34.1, 34.0, 22.9, 22.9, 22.8, 22.5 ppm. **HR-MS** (ESI+): calcd. for $C_{39}H_{26}^+$ $[M]^+$: 494.20290, found 494.20311.
Due to the presence of all the possible stereoisomers in varying ratios, ^{1}H NMR could not be integrated accurately.

2. In-situ NMR irradiation and relaxation experiments

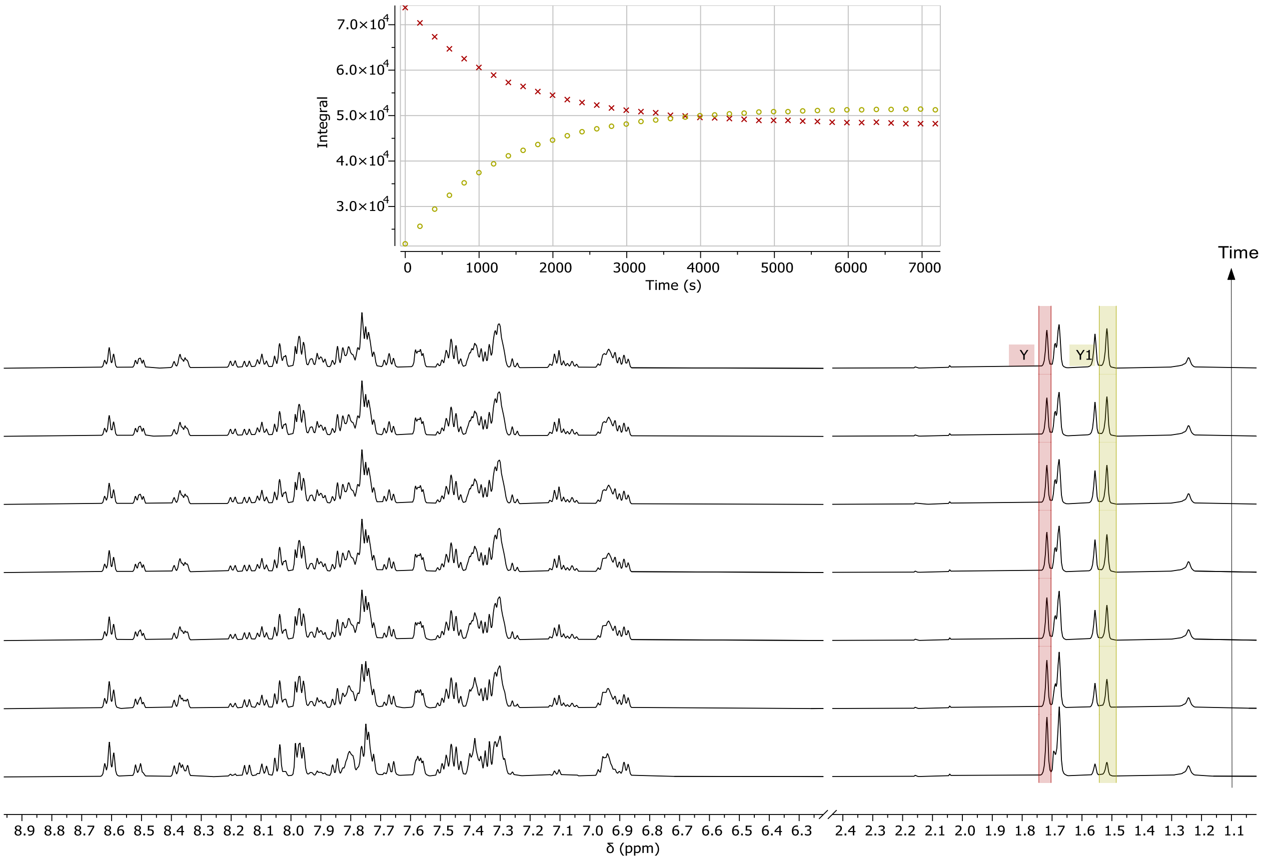

Figure S2. ^{1}H NMR (500 MHz, CD_2Cl_2, –45 °C) monitoring of the photoisomerization of a pristine mixture of isomers (as obtained from synthesis) of **M2** upon irradiation with 405 nm light, showing an enrichment of the metastable states.

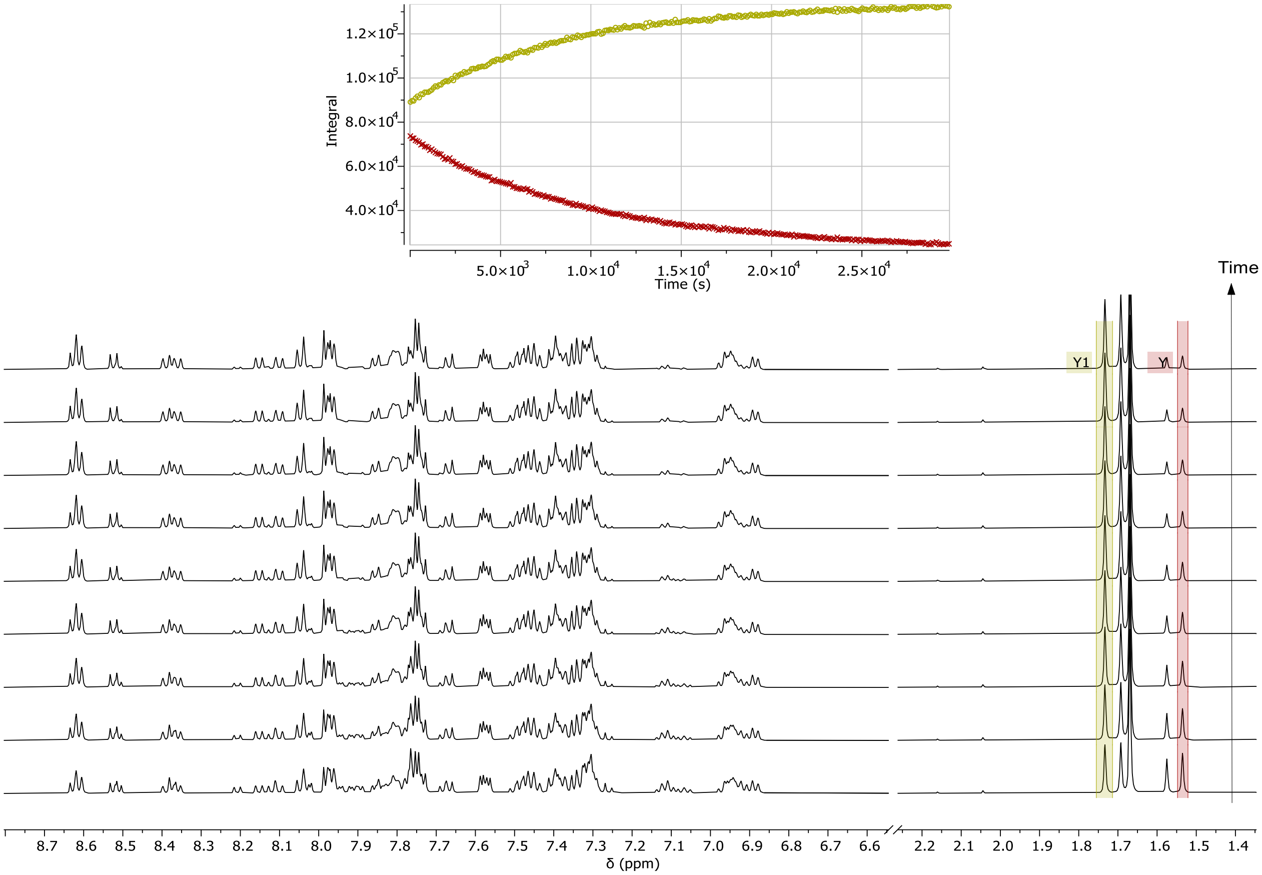

Figure S3. ¹H NMR (500 MHz, CD_2Cl_2, –20 °C) monitoring of the partial thermal relaxation (THI) of the metastable isomers of **M2** into their stable counterparts.

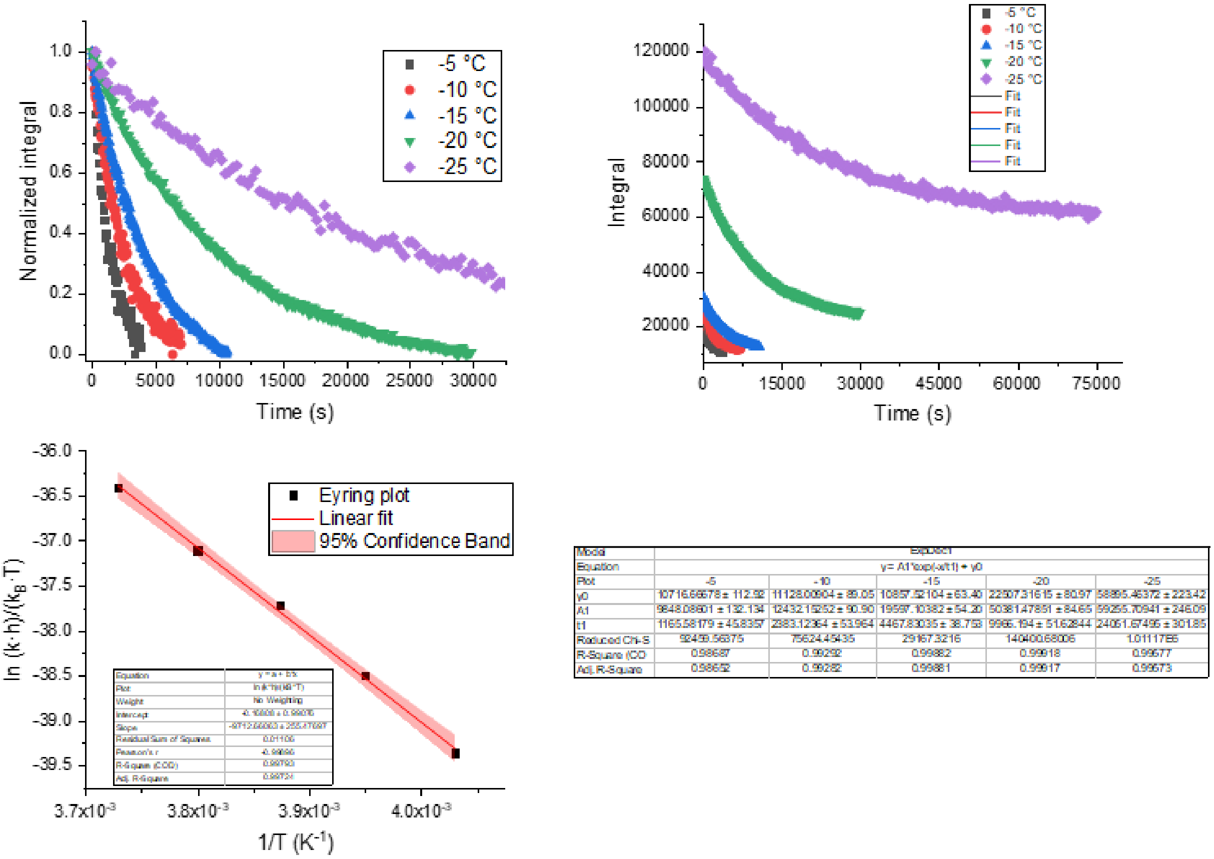

Model	ExpDec1				
Equation	y= A1*exp(-x/t1) + y0				
Plot	-5	-10	-15	-20	-25
y0	10716.66678 ± 112.92	11128.00904 ± 89.05	10857.52104 ± 63.40	22507.31615 ± 80.97	58895.46372 ± 223.42
A1	9848.08801 ± 132.134	12432.15252 ± 90.90	19597.10382 ± 54.20	50381.47851 ± 84.65	59255.70941 ± 246.09
t1	1165.58179 ± 45.8357	2383.12364 ± 53.964	4467.83035 ± 38.753	9986.194 ± 51.62844	24061.67495 ± 301.85
Reduced Chi-S	92409.56375	75624.45435	29167.3216	140400.68006	1.01117E6
R-Square (CO	0.98687	0.99292	0.99882	0.99918	0.99677
Adj. R-Square	0.98652	0.99282	0.99881	0.99917	0.99673

Figure S4. Eyring analysis of thermal helix inversion from metastable **M2** to stable **M2**. The analysis was performed by fitting the ^{1}H NMR integral of the signal at 1.52 ppm (see Figure S3) at 5 different temperatures.

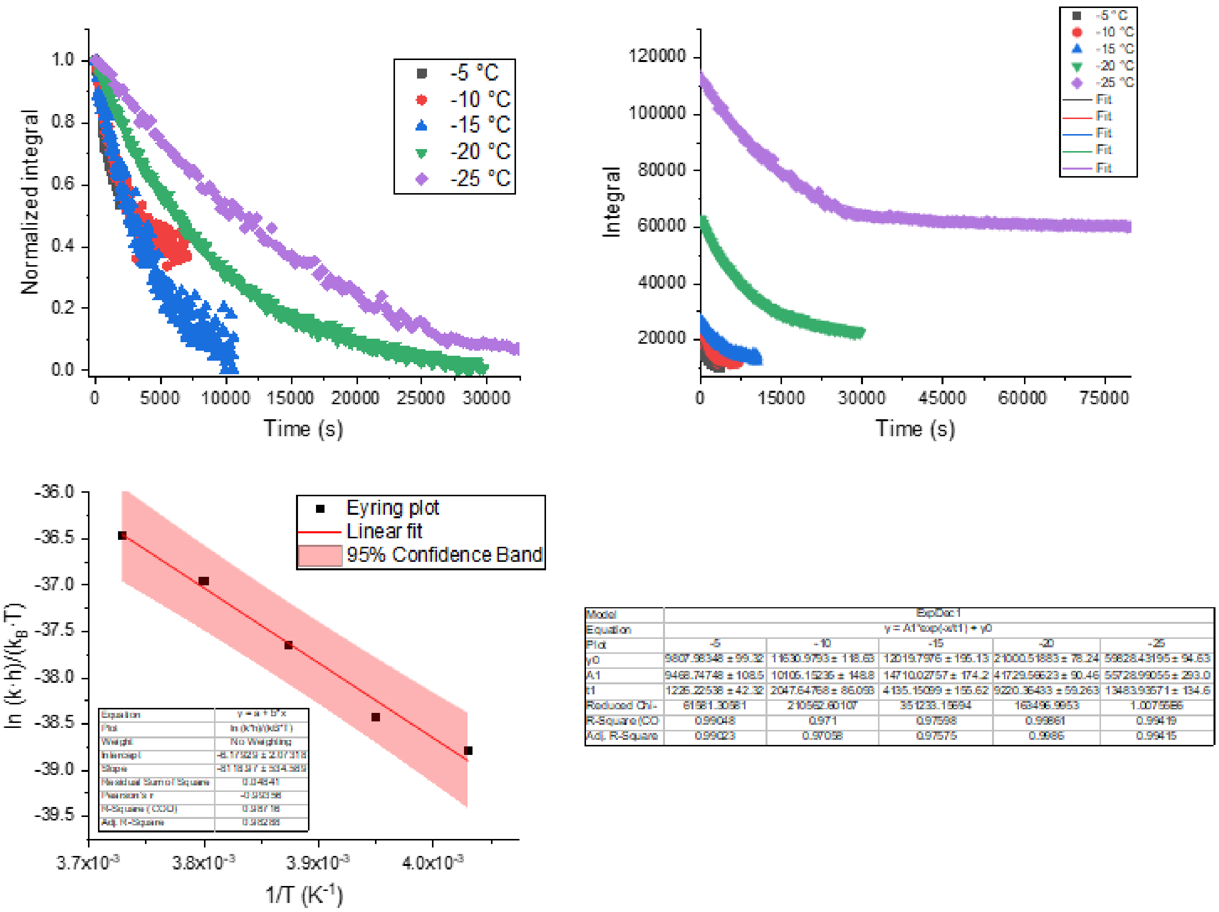

Equation	y = a + b*x
Plot	ln (k*h)/(kB*T)
Weight	No Weighting
Intercept	-6.17929 ± 2.07318
Slope	-8118.97 ± 534.389
Residual Sum of Squares	0.04841
Pearson's r	-0.99358
R-Square (COD)	0.98718
Adj. R-Square	0.98288

Model	ExpDec1				
Equation	y = A1*exp(-x/t1) + y0				
Plot	-5	-10	-15	-20	-25
y0	9807.98348 ± 99.32	11630.9793 ± 118.63	12019.7976 ± 195.13	21000.51883 ± 78.24	59828.43195 ± 94.63
A1	9468.74748 ± 108.5	10106.15236 ± 148.8	14710.02757 ± 174.2	41729.98623 ± 90.46	55728.99055 ± 293.0
t1	1226.22538 ± 42.32	2047.64768 ± 86.093	4135.15099 ± 155.62	9220.36433 ± 59.263	13483.93671 ± 134.6
Reduced Chi-	61581.30981	210562.60107	351233.15694	163496.9953	1.0075586
R-Square (CO	0.99048	0.971	0.97598	0.99861	0.99419
Adj. R-Square	0.99023	0.97058	0.97575	0.9986	0.99415

Figure S5. Eyring analysis of thermal helix inversion from metastable **M2** to stable **M2**. The analysis was performed by fitting the ^{1}H NMR integral of the signal at 1.56 ppm (see Figure S3) at 5 different temperatures.

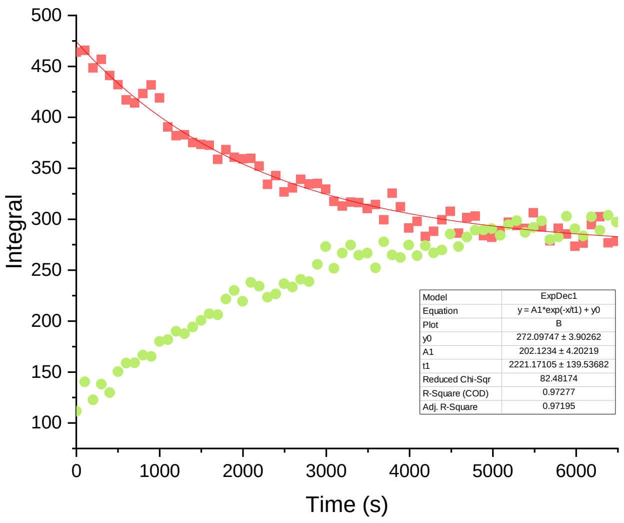

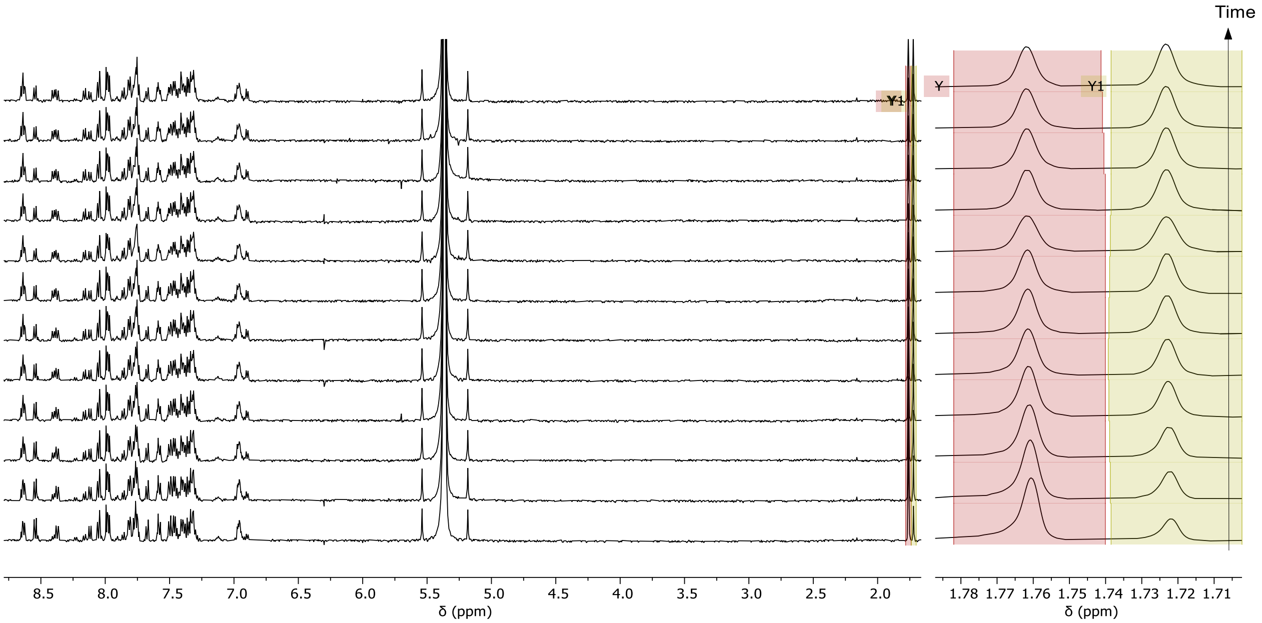

Figure S6. ^{1}H NMR (500 MHz, CD_2Cl_2, 15 °C) monitoring of the thermal *E*/*Z* isomerization of an isomerically enriched sample of **M2** at 15 °C.

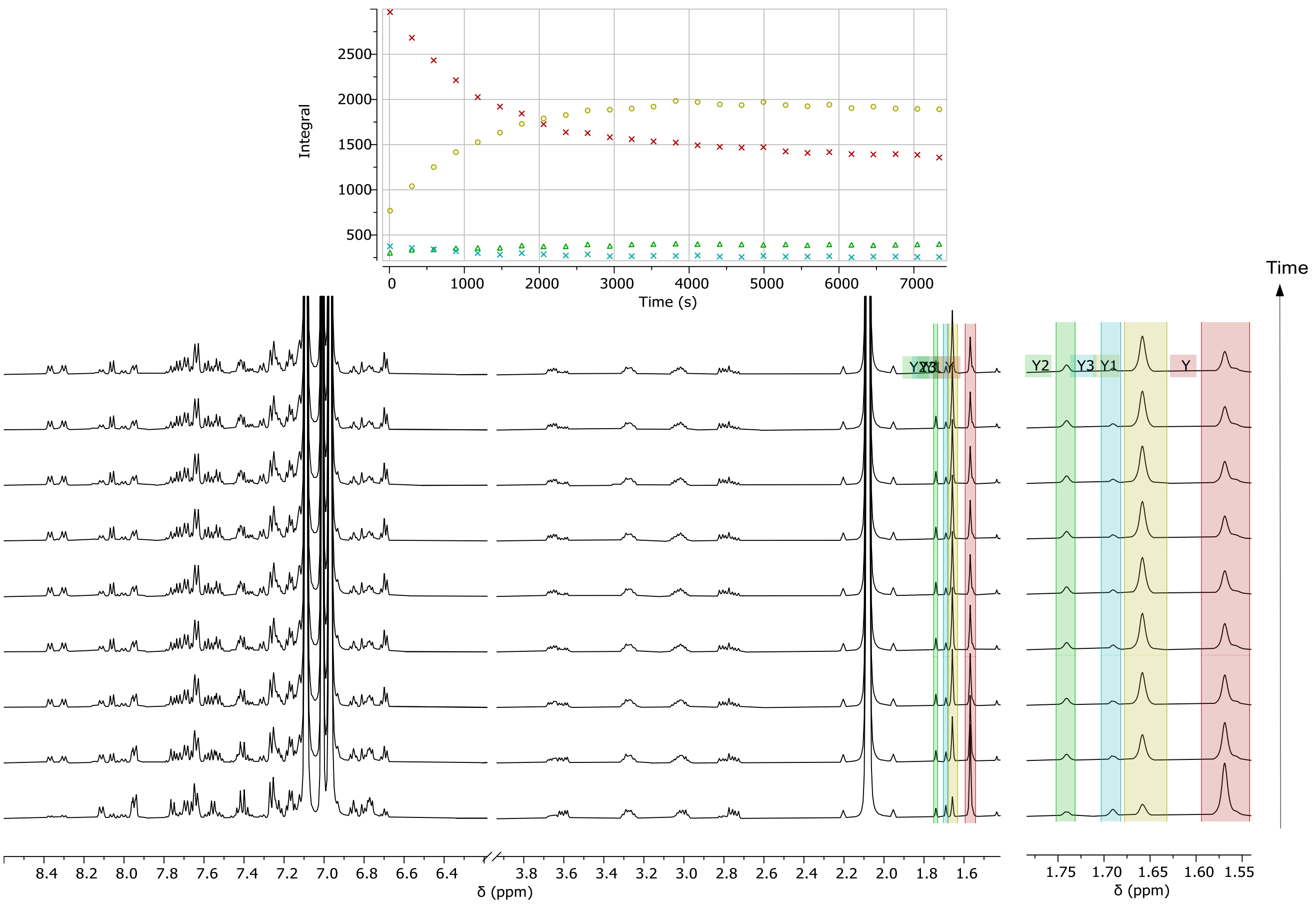

Figure S7. ^{1}H NMR (500 MHz, toluene-d8, 15 °C) monitoring of the photoisomerization of a sample enriched in a single metastable isomer of **M3** at 15 °C upon irradiation with 455 nm light.

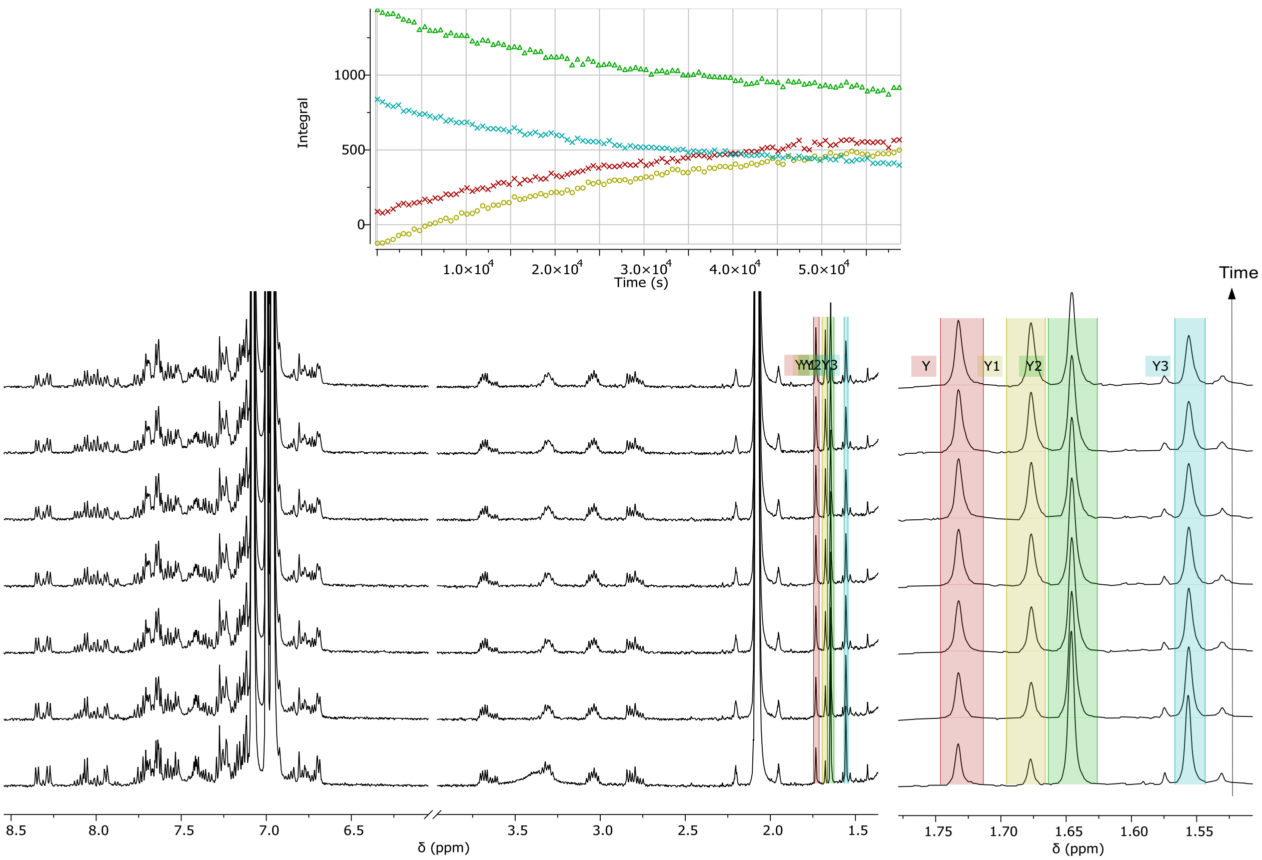

Figure S8. ^{1}H NMR (500 MHz, toluene-d8, 40 °C) monitoring of the partial thermal relaxation (THI) of the stable isomer of **M3** generated upon photoisomerization of its metastable counterpart (Figure S7) to yield an isomeric mixture analogous to the one obtained from the pristine sample before isomeric enrichment.

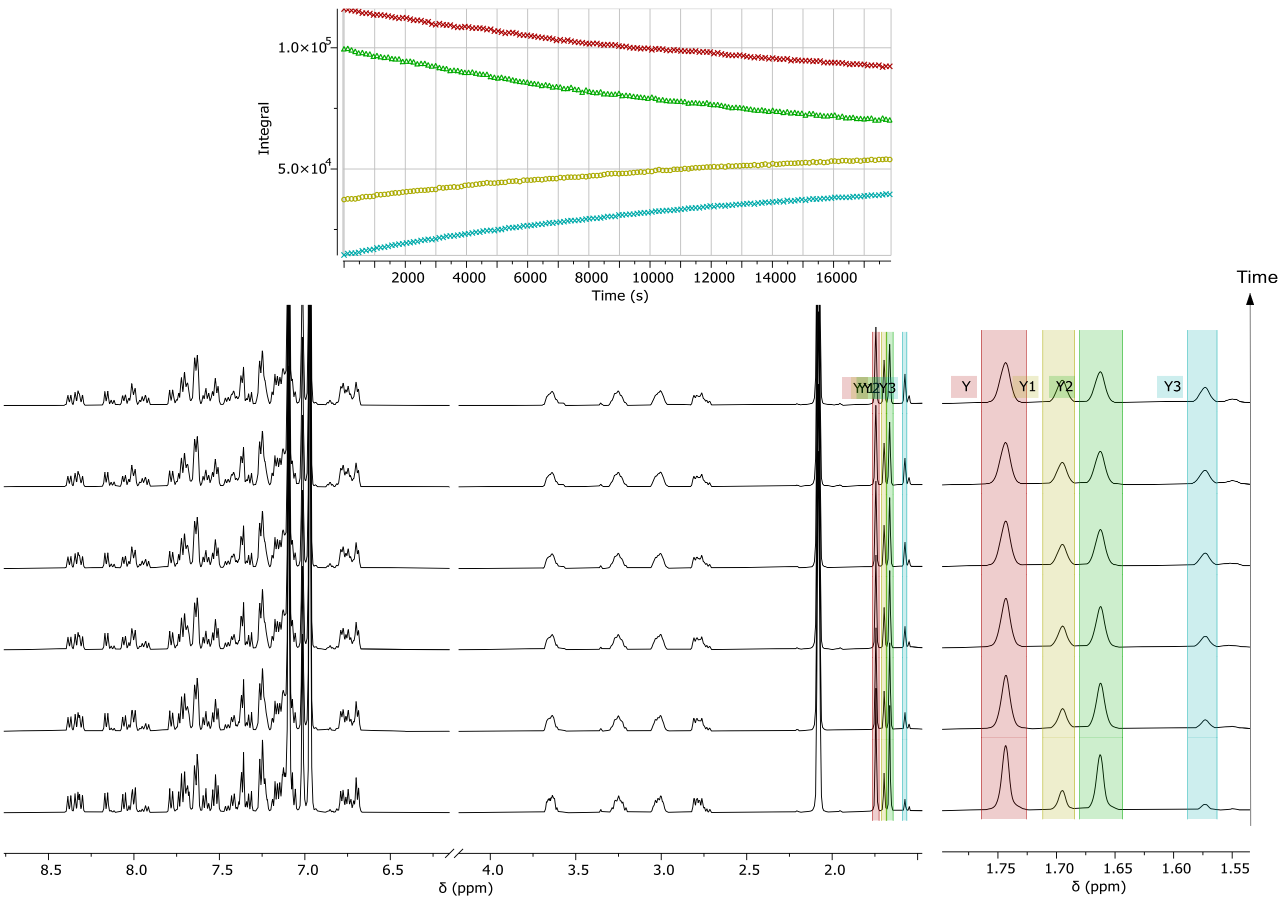

Figure S9. ^{1}H NMR (500 MHz, toluene-d8, 15 °C) monitoring of the photoisomerization of a sample enriched in two isomers of **M3** at 15 °C upon irradiation with 405 nm light.

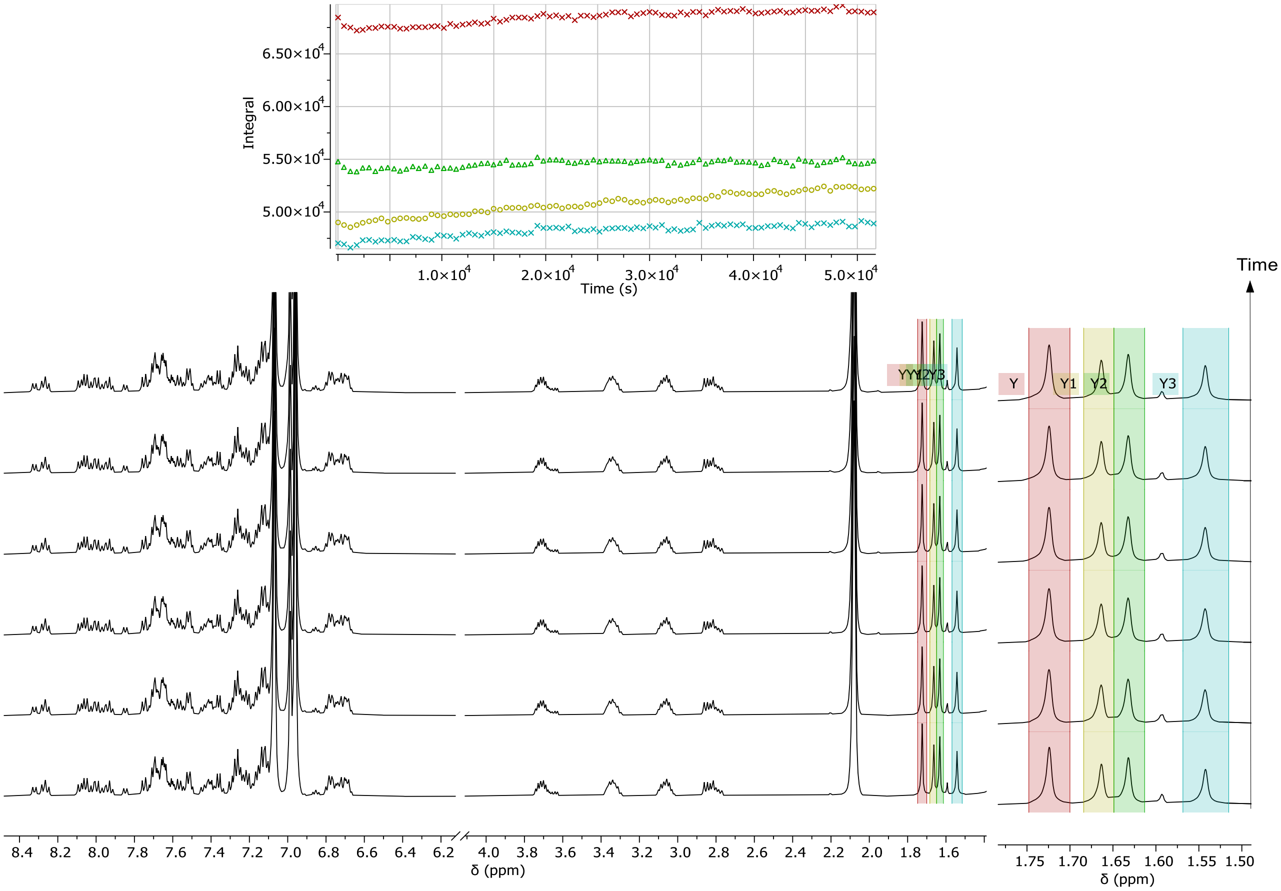

Figure S10. ^{1}H NMR (500 MHz, toluene-d8, 40 °C) monitoring of the heating of an isomeric mixture of **M3** obtained after photoisomerization of an enriched sample of two isomers (Figure S9) showing no signs of THI and negligible changes.

3. Chiral Resolution and CD spectra

Analytical separation of racemic **M2** was achieved on a CHIRALPAK IBN-5 column (4.6x200mm, 5µm particle size) from Daicel Corporation, using a mobile phase of *n*-heptane/CH_2Cl_2 (8:2) and flowrate of 1 mL/min.

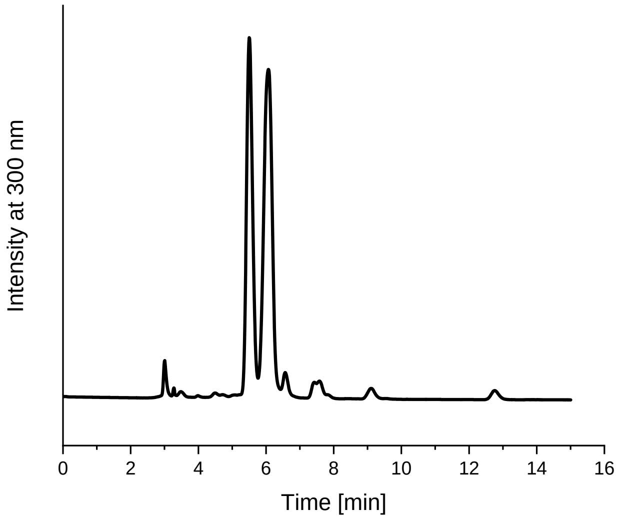

Figure S11. Analytical HPLC chromatogram of **M2**.

Both isolated fractions were isolated on a semipreparative CHIRALPAK IBN-5 column (10x200mm, 5 5µm particle size) and a flowrate of 4 mL/min. The isolated fractions were reinjected into the HPLC using the analytical separation conditions to confirm enantiopurity.

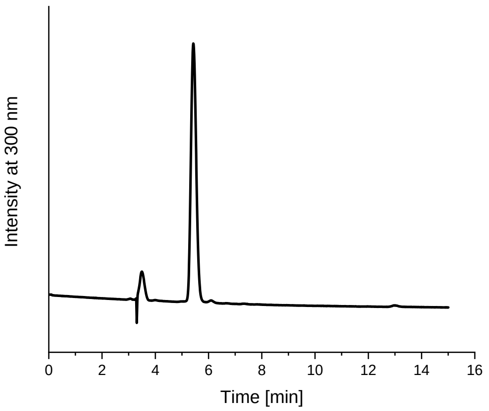

Figure S12. Analytical HPLC chromatogram of the isolated first eluting peak of **M2**.

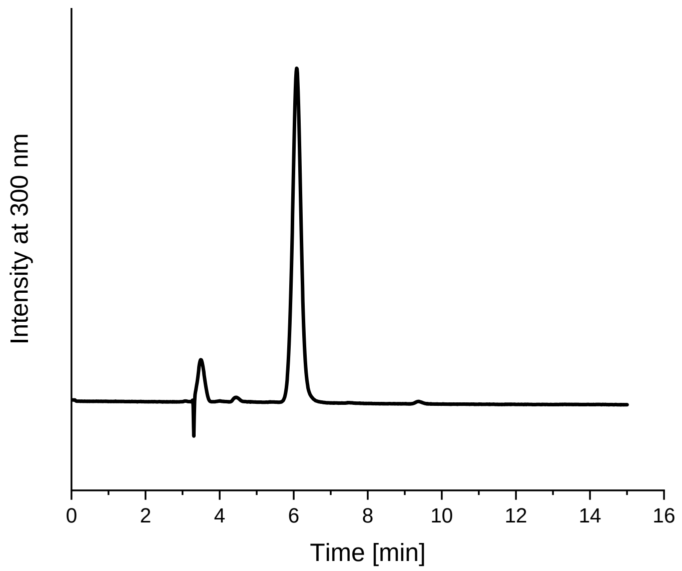

Figure S13. Analytical HPLC chromatogram of the isolated second eluting peak of **M2**.

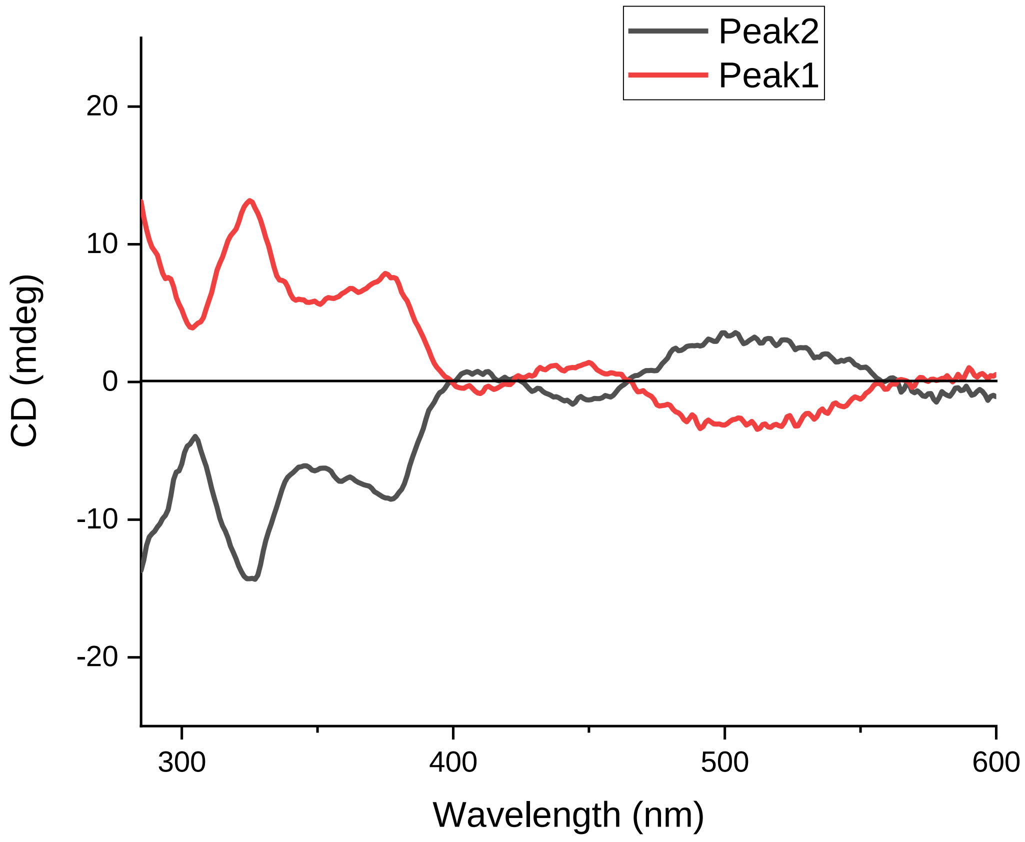

Figure S14. CD spectra of the first (red line) and second (black line) eluting peak of **M2** in toluene.

Analytical separation of racemic **M3** was achieved on a CHIRALPAK IE column (4.6x200mm, 5µm particle size) from Daicel Corporation, using a mobile phase of *n*-heptane/CH_2Cl_2 (8:2) and flowrate of 1 mL/min. Separation into 6 different peaks was observed in the chromatogram, whereas the fourth eluting peak (P4) is broad and consists of multiple compounds, as deduced from the shoulder peak (Figure S15).

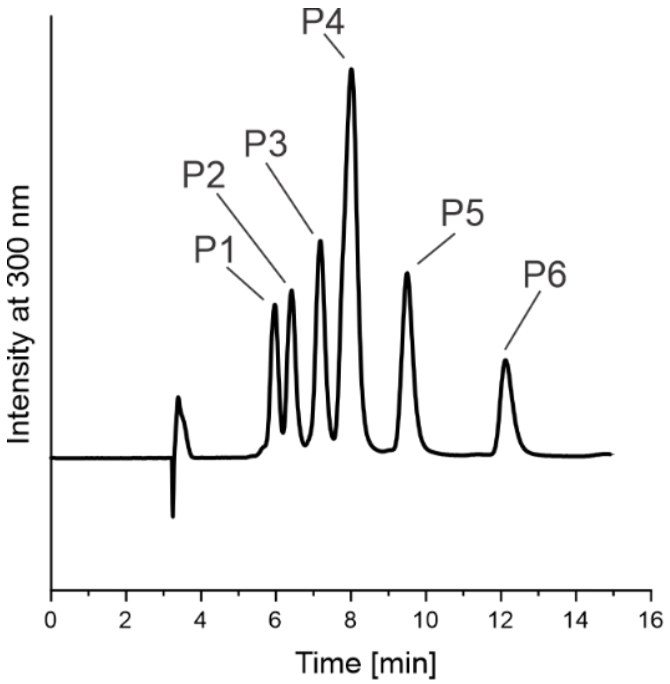

Figure S15. Analytical HPLC chromatogram of racemic **M3**. Peak 4 (P4) is very broad and consists of multiple compounds.

Peak 5 (P5) and peak 6 (P6) were isolated on a semipreparative CHIRALPAK IE column (10x200mm, 5 5µm particle size) and a flowrate of 4.5 mL/min. After evaporation of the solvent, the yellow solid was redissolved in CH_2Cl_2 and the solution was relaxed under ambient conditions for 2 weeks. Reinjecting the relaxed solution into the analytical separation conditions described above revealed the formation of different sets of peaks for P5 and P6, indicating that they represent relaxed mixtures of different chirality ((*P*) or (*M*)).

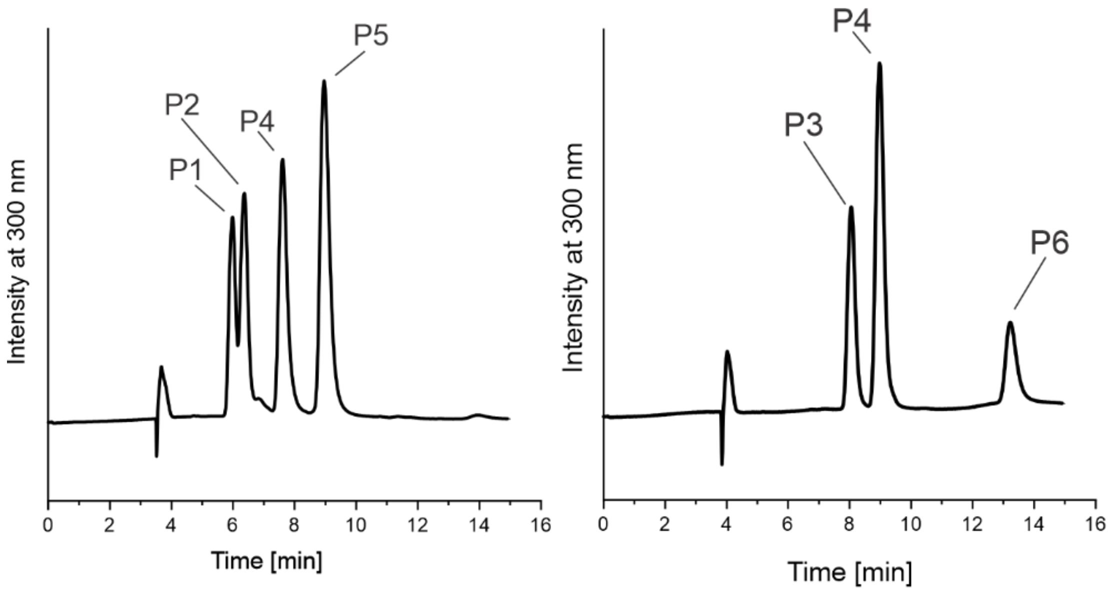

Figure S16. Analytical HPLC chromatogram of isolated and relaxed P5 (left) and P6 (right). In the right chromatogram, P4 is broad and likely consists of two compounds.

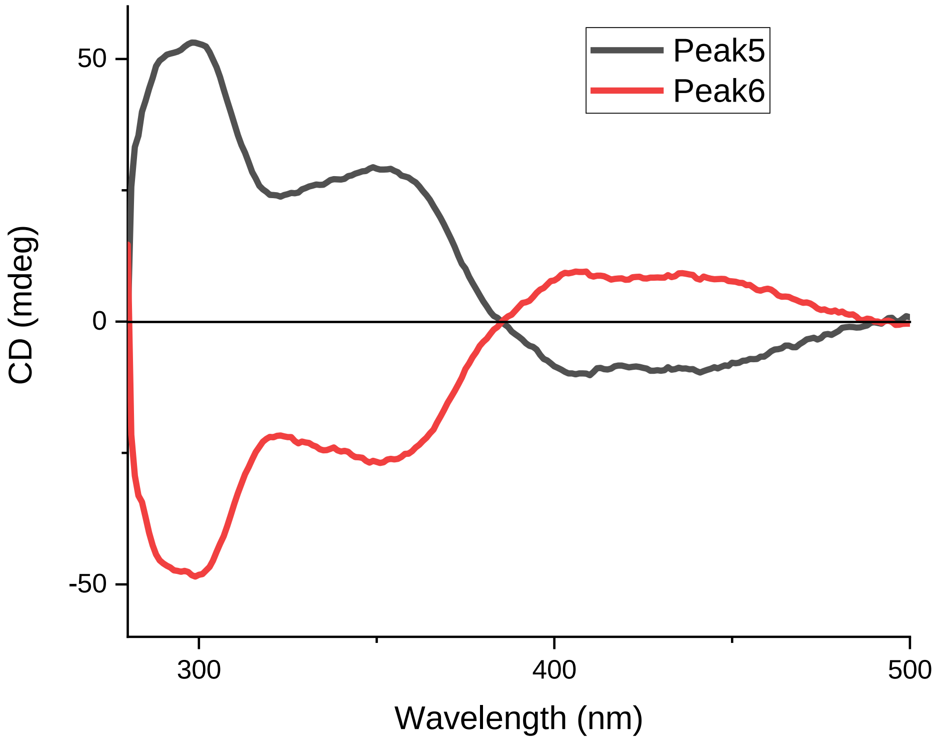

Figure S17. CD spectra of isolated and relaxed P5 (black line) and P6 (red line) of **M3** in toluene.

4. Enantiomerization Barrier

The racemization barrier of **5** was determined by dynamic chromatography on a chiral stationary phase at 70°C oven temperature, using a Shimadzu Prominence HPLC system. The elution profile (Figure S18) was obtained with a *n*-heptane/CH_2Cl_2 (85:15) mobile phase (1 mL flowrate) and a CHIRALPAK IBN-5 column (4.6x200 mm, 5 µm particle size) from Daicel Corporation.

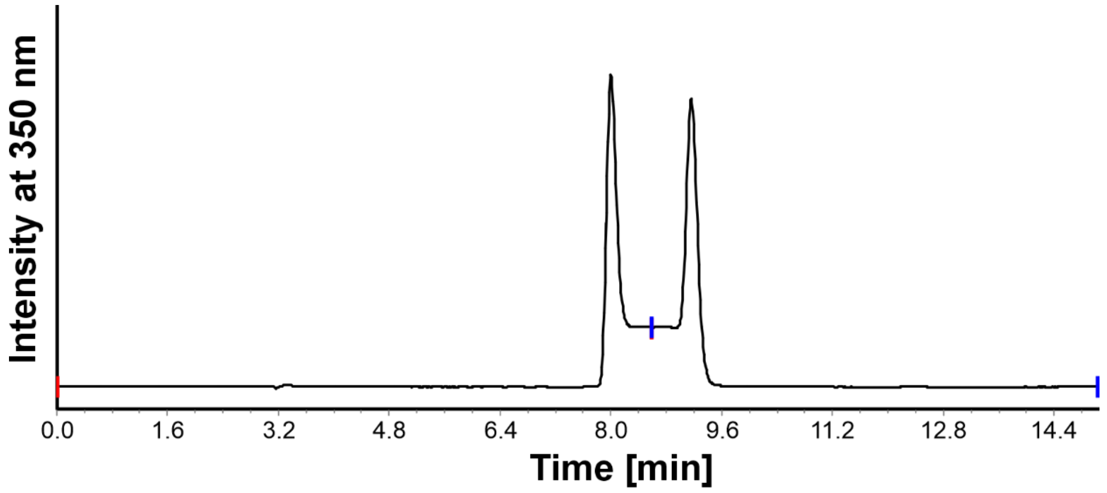

Figure S18. HPLC chromatogram of **5** at 70°C.

The elution profile was analyzed using the DCXplorer software developed by Trapp and coworkers,[9] determining an enantiomerization rate constant of k_e = 1.15x10^{-3} s^{-1}. This results in an enantiomerization barrier at 70 °C of

$$\Delta G^{\ddagger}_{e(343.15)} = 103 \text{ kJ/mol}$$

according to[10]

$$\Delta G^{\ddagger}_{e(T)} = RT[\ln(\frac{k_B T}{h}) - \ln(k_e)]$$

5. Computational analysis

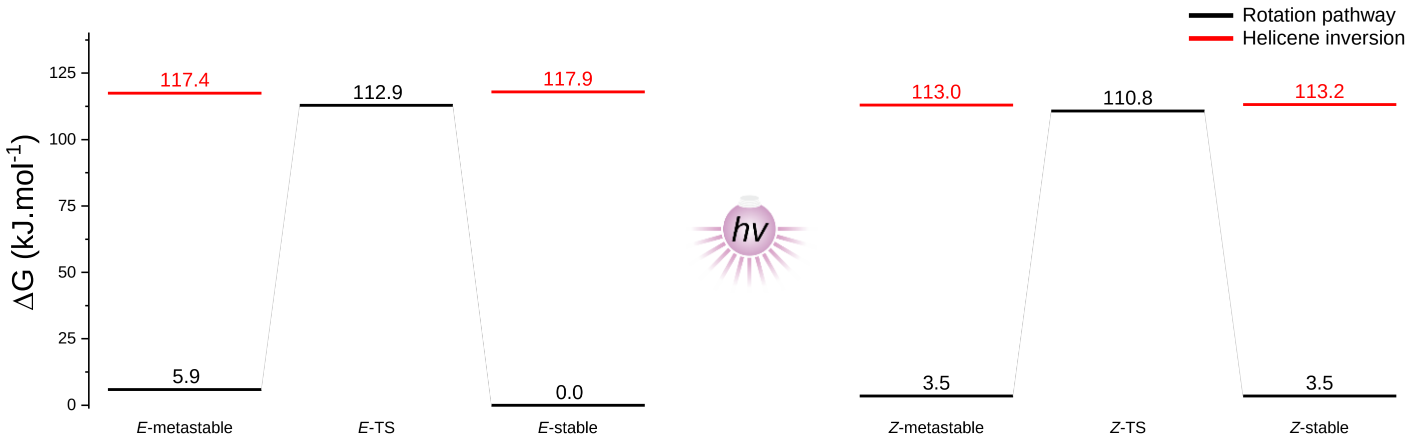

Figure S19. Calculated mechanistic pathways for the rotation and lower helicene inversion (enantiomerization) for **M1** at the r^{2}SCAN-3c/CPCM(CH_2Cl_2) level of theory. Energies are given in kJ.mol^{-1} at 25 °C.

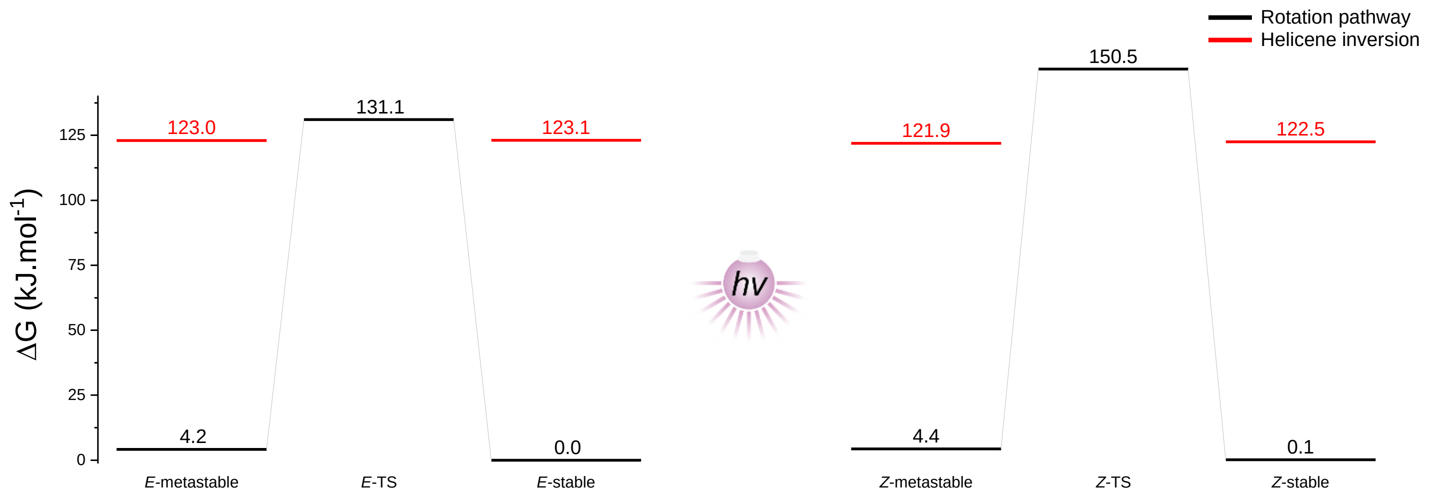

Figure S20. Calculated mechanistic pathways for the rotation and lower helicene inversion (enantiomerization) for **MA** at the r^{2}SCAN-3c/CPCM(CH_2Cl_2) level of theory. Energies are given in kJ.mol^{-1} at 25 °C.

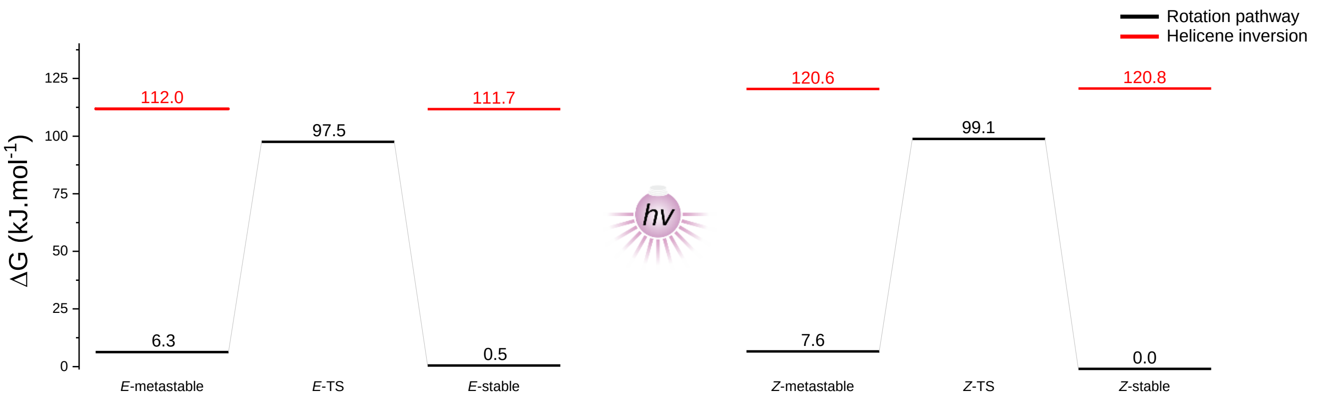

Figure S21. Calculated mechanistic pathways for the rotation and lower helicene inversion (enantiomerization) for **MB** at the r^{2}SCAN-3c/CPCM(CH_2Cl_2) level of theory. Energies are given in kJ.mol^{-1} at 25 °C.

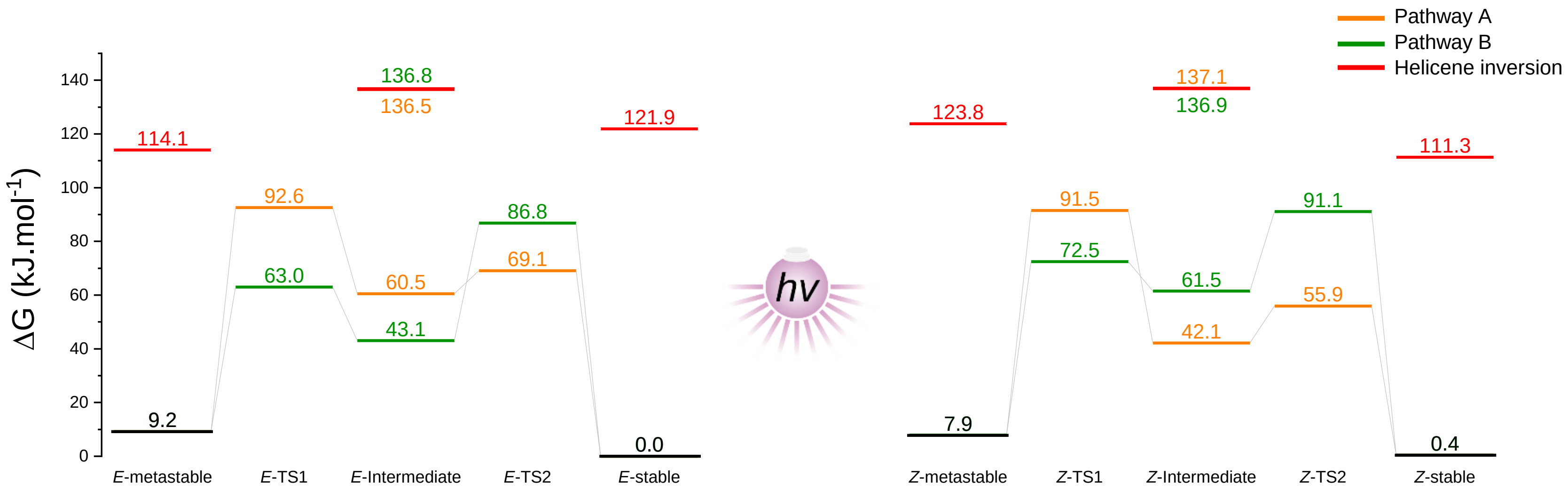

Figure S22. Calculated mechanistic pathways for the rotation and lower helicene inversion (enantiomerization) for **MC** at the r^{2}SCAN-3c/CPCM(CH_2Cl_2) level of theory. Pathway A in orange first involves the helix inversion of the naphthyl moiety of the upper half, followed by the helix inversion of the fluorinated moiety. Pathway B in green first involves the helix inversion of the fluorinated moiety of the upper half, followed by the helix inversion of the naphthyl moiety. The energy level of the bottom-half helicene inversion are represented by red lines. Energies are given in kJ.mol^{-1} at 25 °C

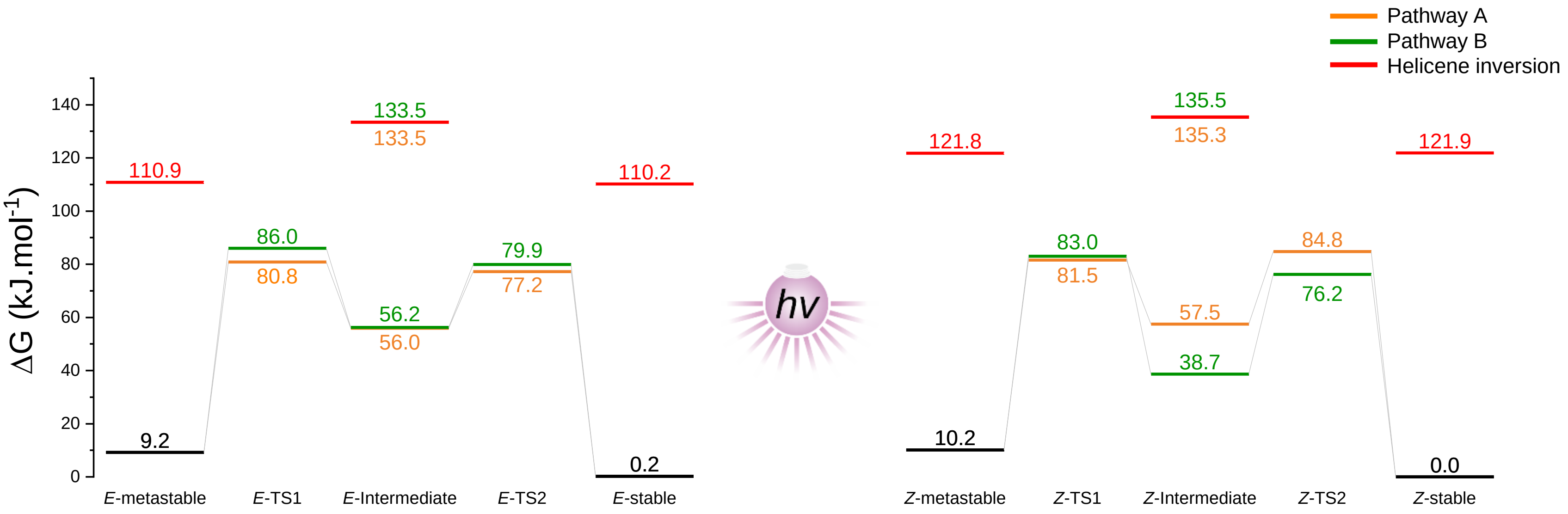

Figure S23. Calculated mechanistic pathways for the rotation and lower helicene inversion (enantiomerization) for **MD** at the r^{2}SCAN-3c/CPCM(CH_2Cl_2) level of theory. Pathway A in orange first involves the helix inversion of the naphthyl moiety of the upper half, followed by the helix inversion of the tolyl moiety. Pathway B in green first involves the helix inversion of the tolyl moiety of the upper half, followed by the helix inversion of the naphthyl moiety. The energy level of the bottom-half helicene inversion are represented by red lines. Energies are given in kJ.mol^{-1} at 25 °C.

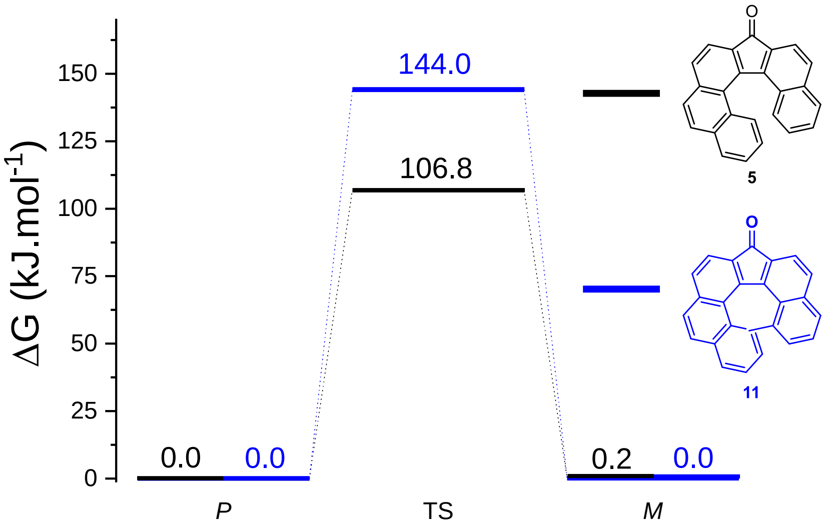

Figure S24. Calculated helicene inversion (enantiomerization) barriers for ketones **5** and **11** at the r^{2}SCAN-3c/CPCM(CH_2Cl_2) level of theory. Energies are given in kJ.mol^{-1} at 25 °C.

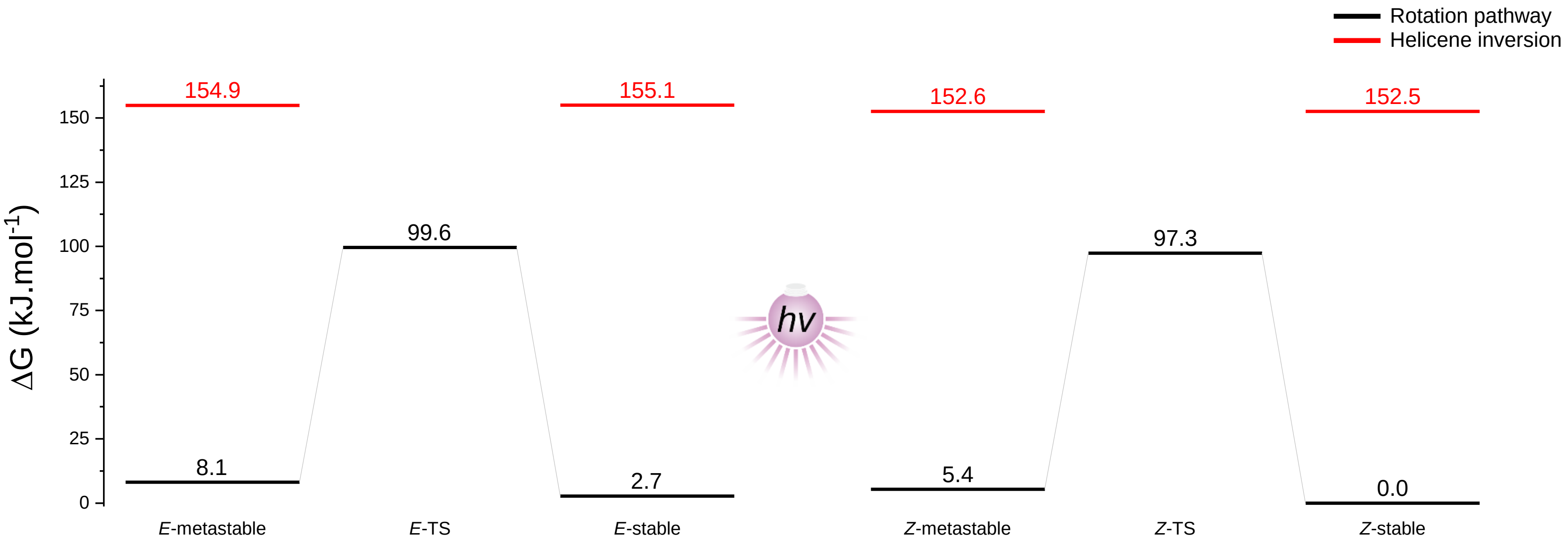

Figure S25. Calculated mechanistic pathways for the rotation and lower helicene inversion (enantiomerization) for **M2** at the r^{2}SCAN-3c/CPCM(CH_2Cl_2) level of theory. Energies are given in kJ.mol^{-1} at 25 °C.

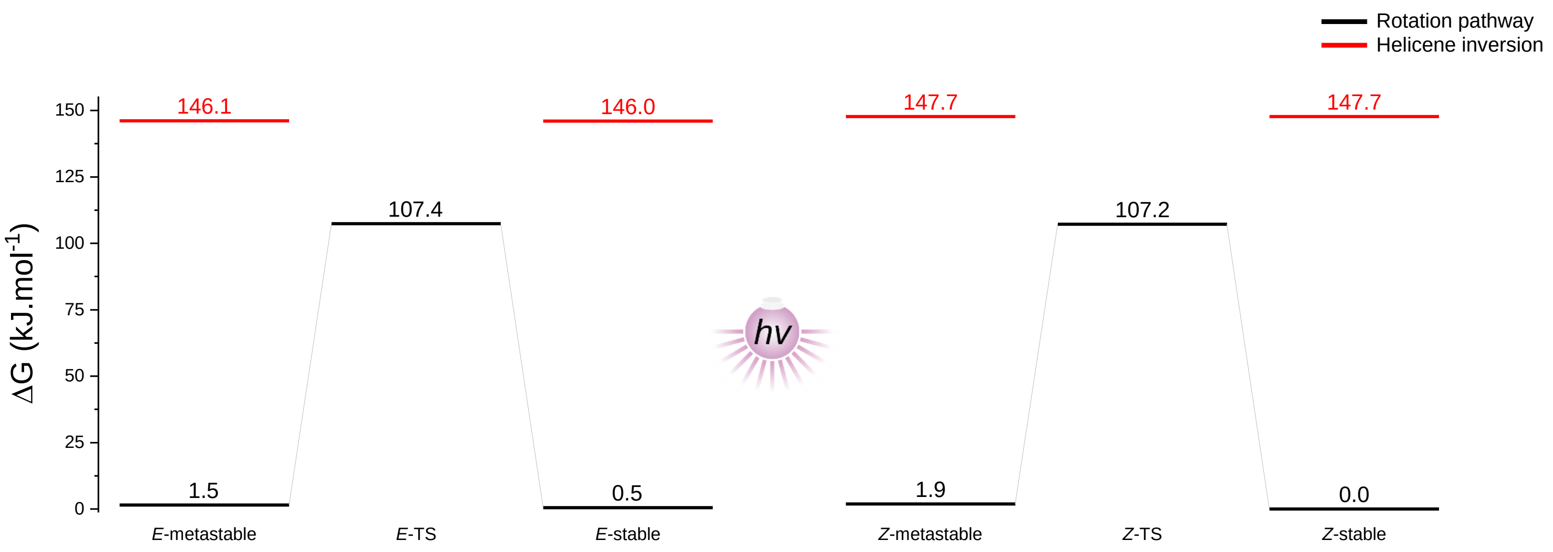

Figure S26. Calculated mechanistic pathways for the rotation and lower helicene inversion (enantiomerization) for **M3** at the r^2SCAN-3c/CPCM(CH_2Cl_2) level of theory. Energies are given in kJ.mol^{-1} at 25 °C

6. NMR spectra of new compounds

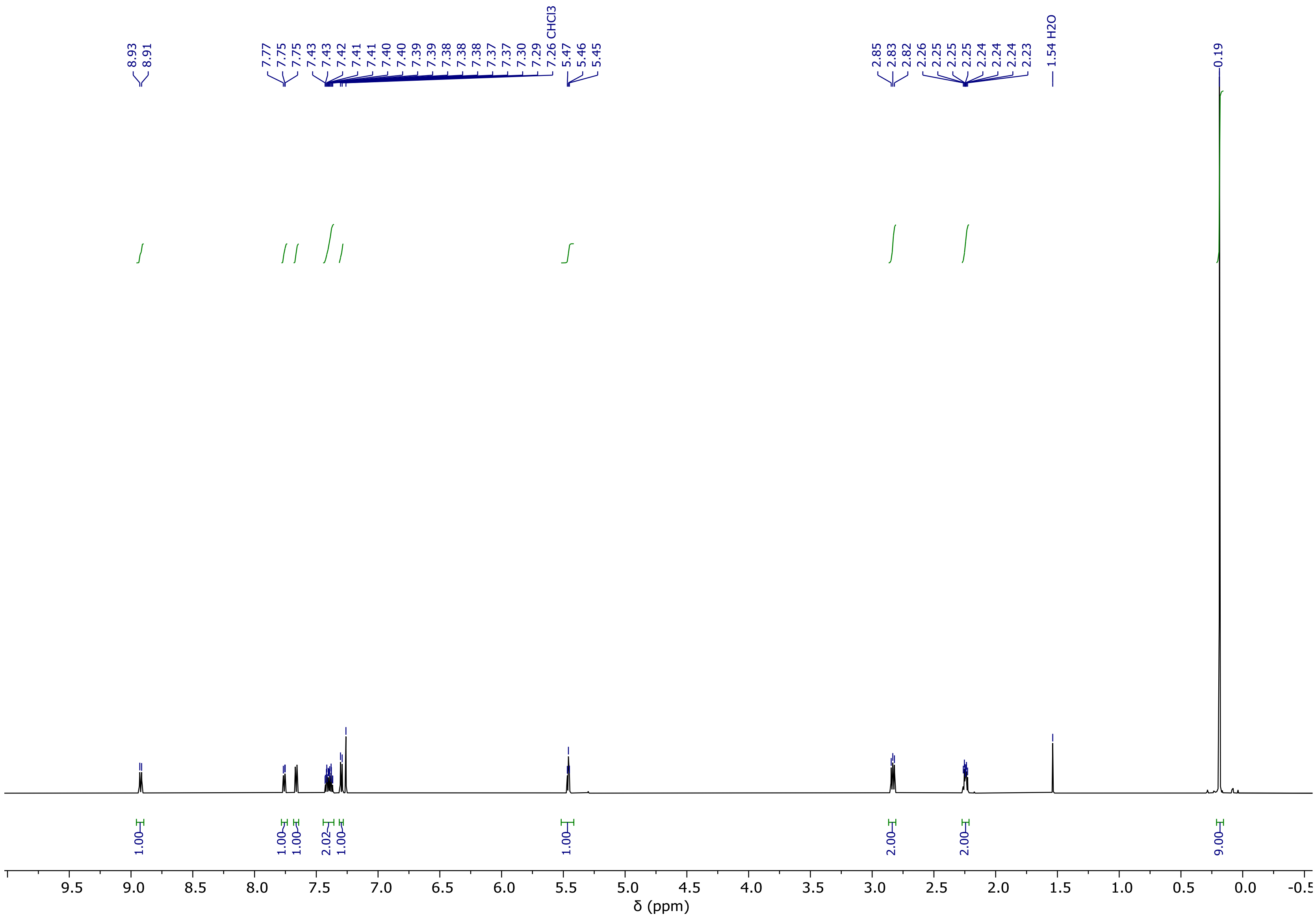

Figure S27. ^{1}H-NMR spectrum of ((1,2-dihydrophenanthren-4-yl)oxy)trimethylsilane (**1**) (600 MHz, $CDCl_3$, 25 °C).

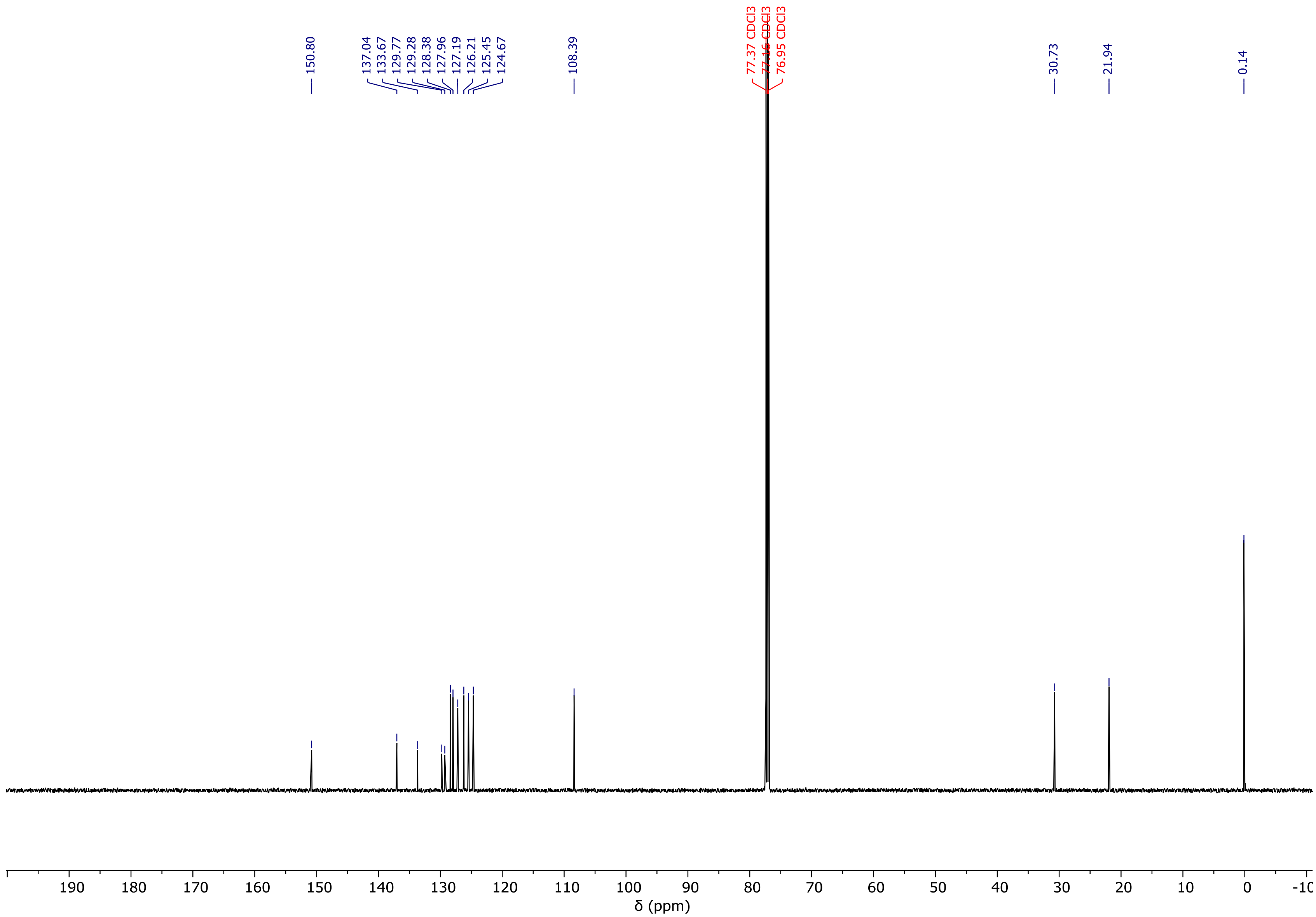

Figure S28. $^{13}C\{^1H\}$-NMR spectrum of ((1,2-dihydrophenanthren-4-yl)oxy)trimethylsilane (**1**) (151 MHz, $CDCl_3$, 25 °C).

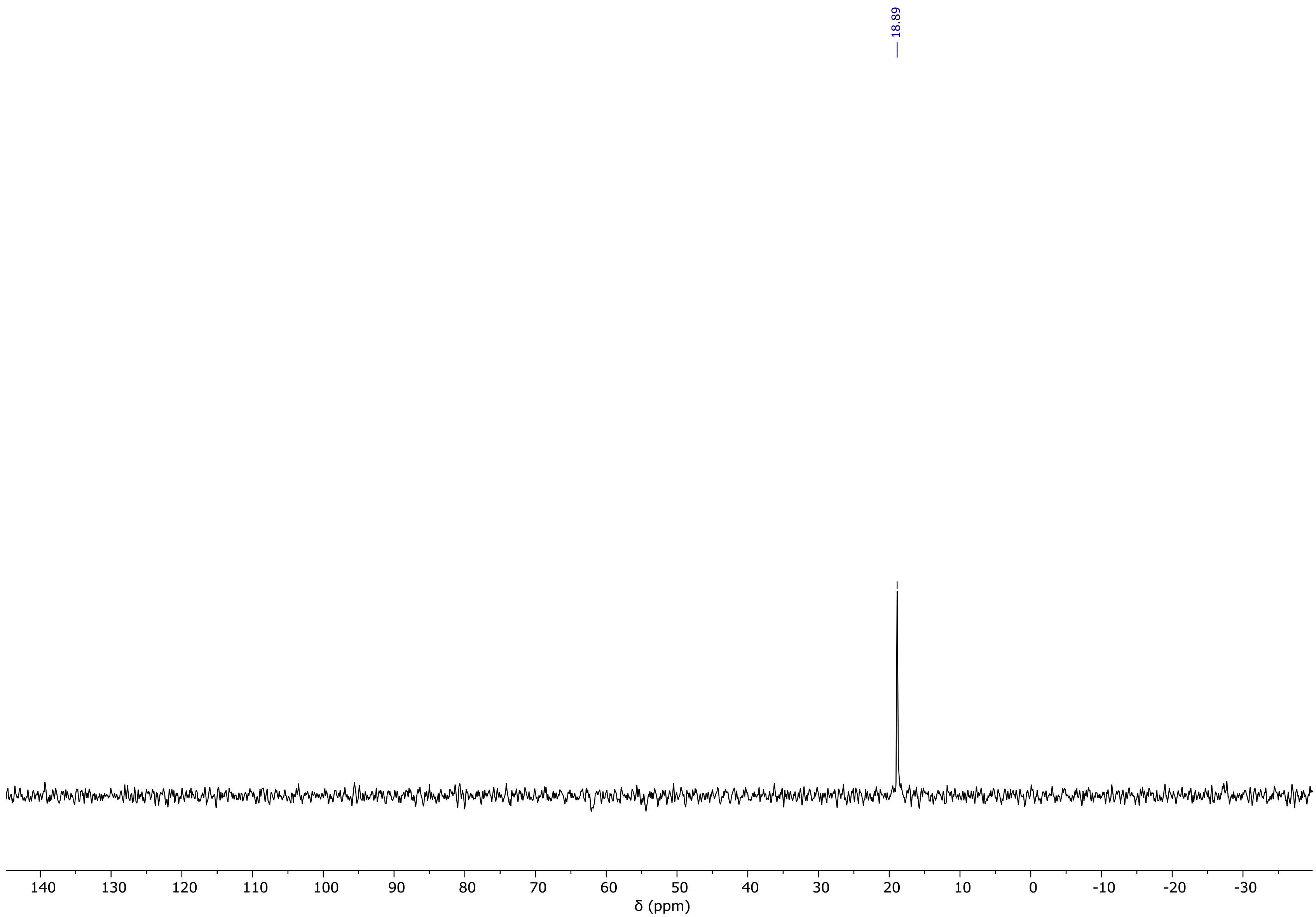

Figure S29. $^{29}Si\{^{1}H\}$-NMR spectrum of ((1,2-dihydrophenanthren-4-yl)oxy)trimethylsilane (**1**) (80 MHz, $CDCl_3$, 25 °C).

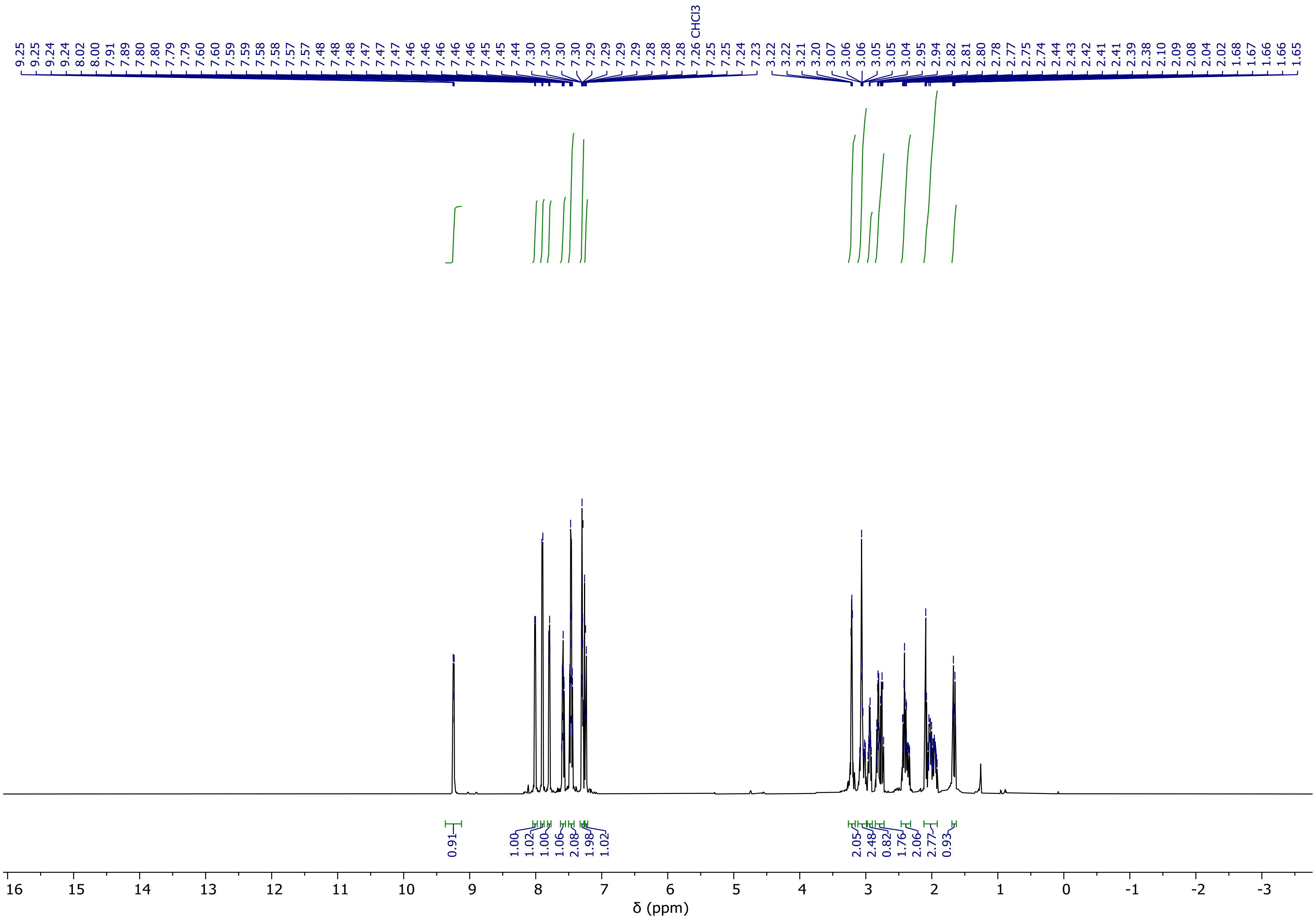

Figure S30. ^{1}H-NMR spectrum of 1,5-diketone **2** (600 MHz, $CDCl_3$, 25 °C).

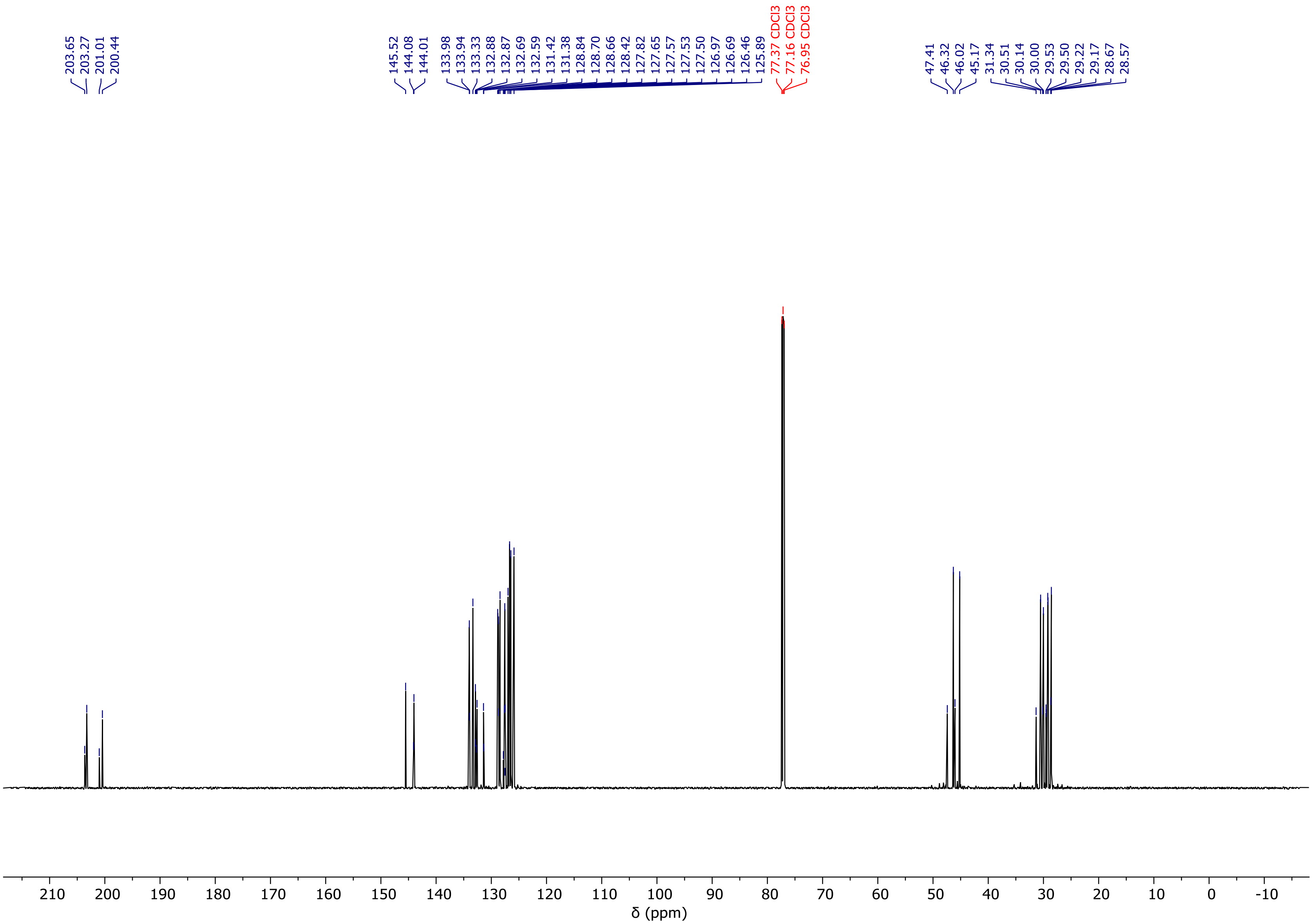

Figure S31. $^{13}C\{^1H\}$-NMR spectrum of 1,5-diketone **2** (151 MHz, $CDCl_3$, 25 °C).

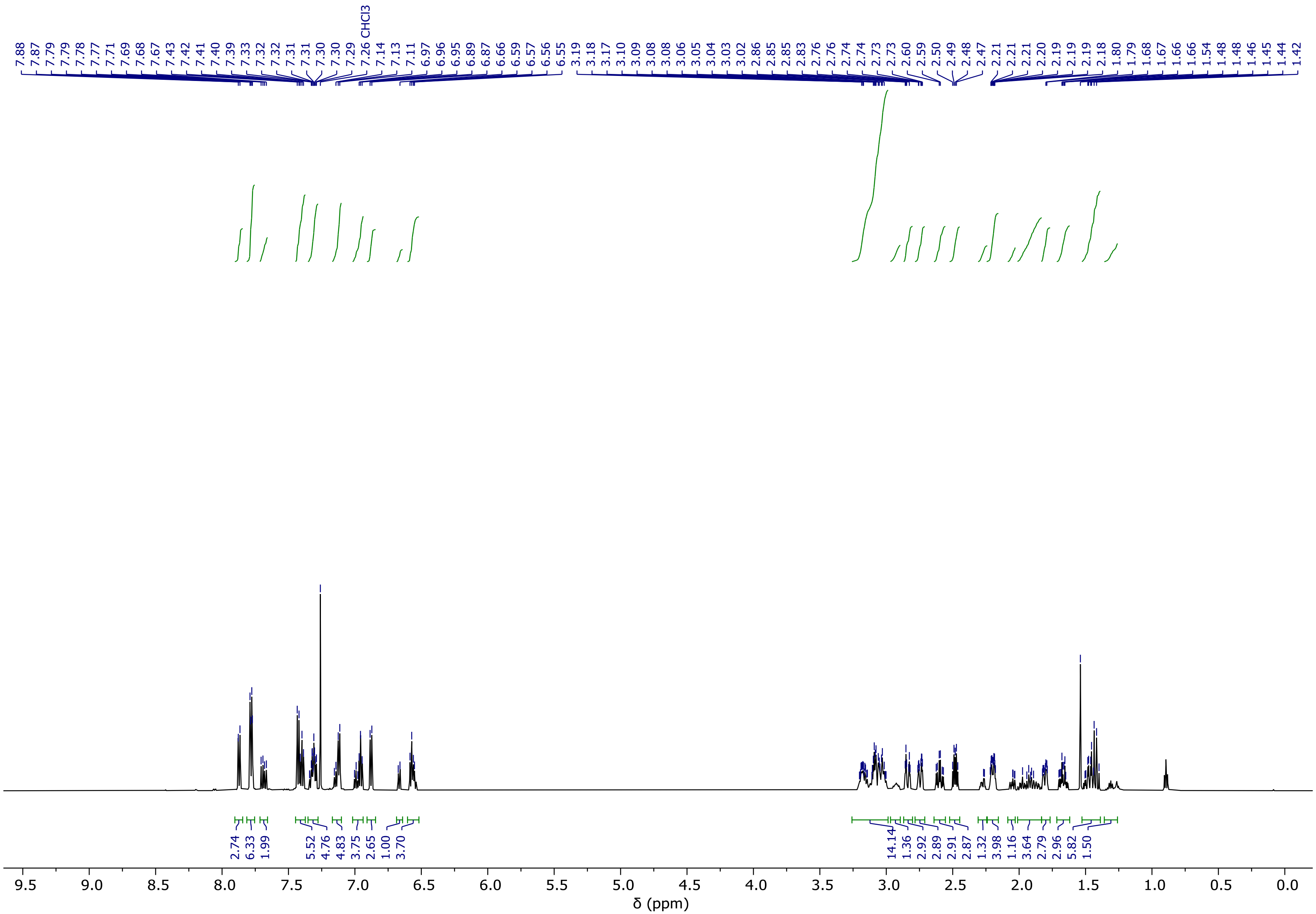

Figure S32. ^{1}H-NMR spectrum of hydrogenated fluoreno[6]helicene **3** (600 MHz, $CDCl_3$, 25 °C).

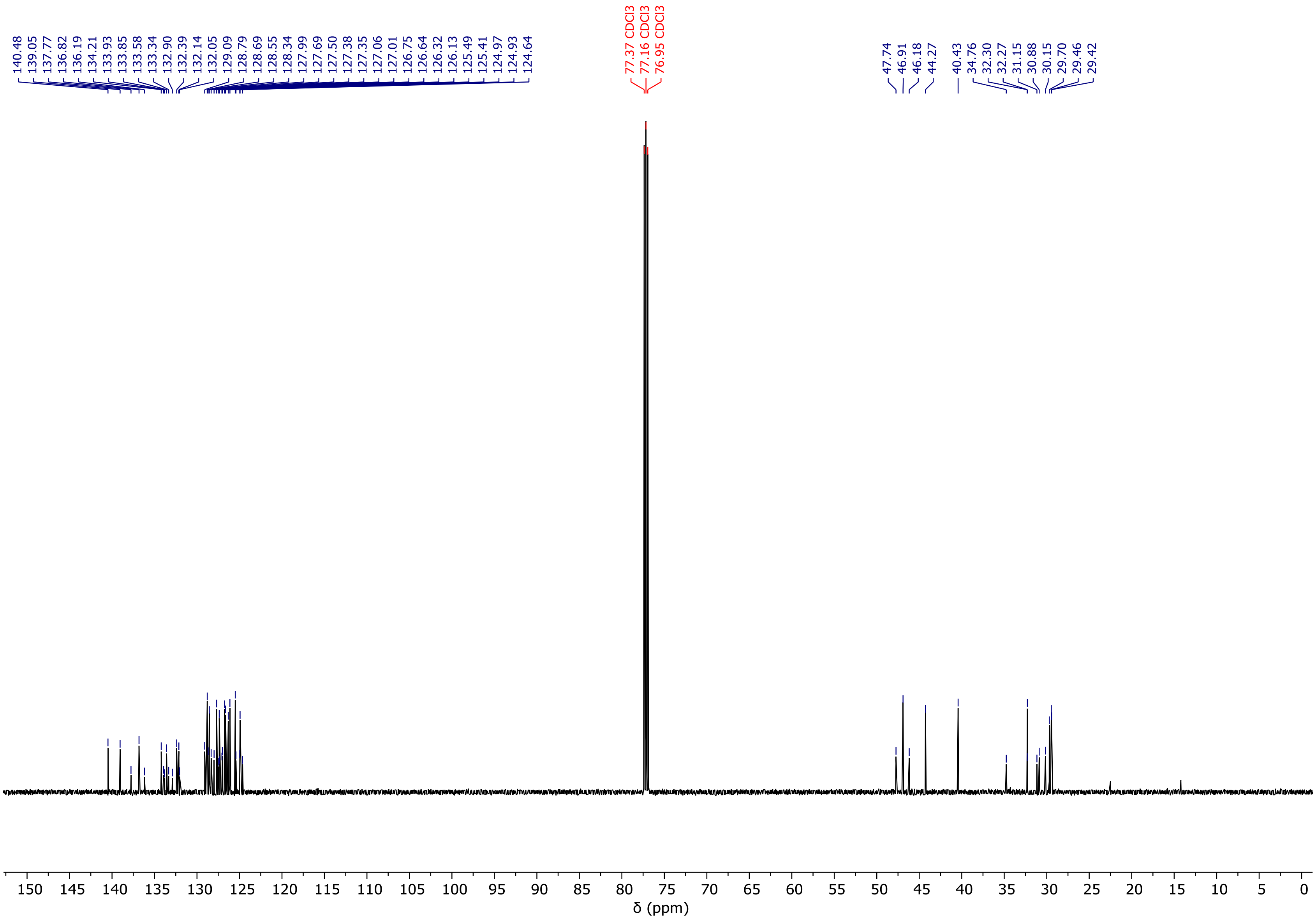

Figure S33. $^{13}C\{^{1}H\}$-NMR spectrum of hydrogenated fluoreno[6]helicene **3** (151 MHz, $CDCl_3$, 25 °C).

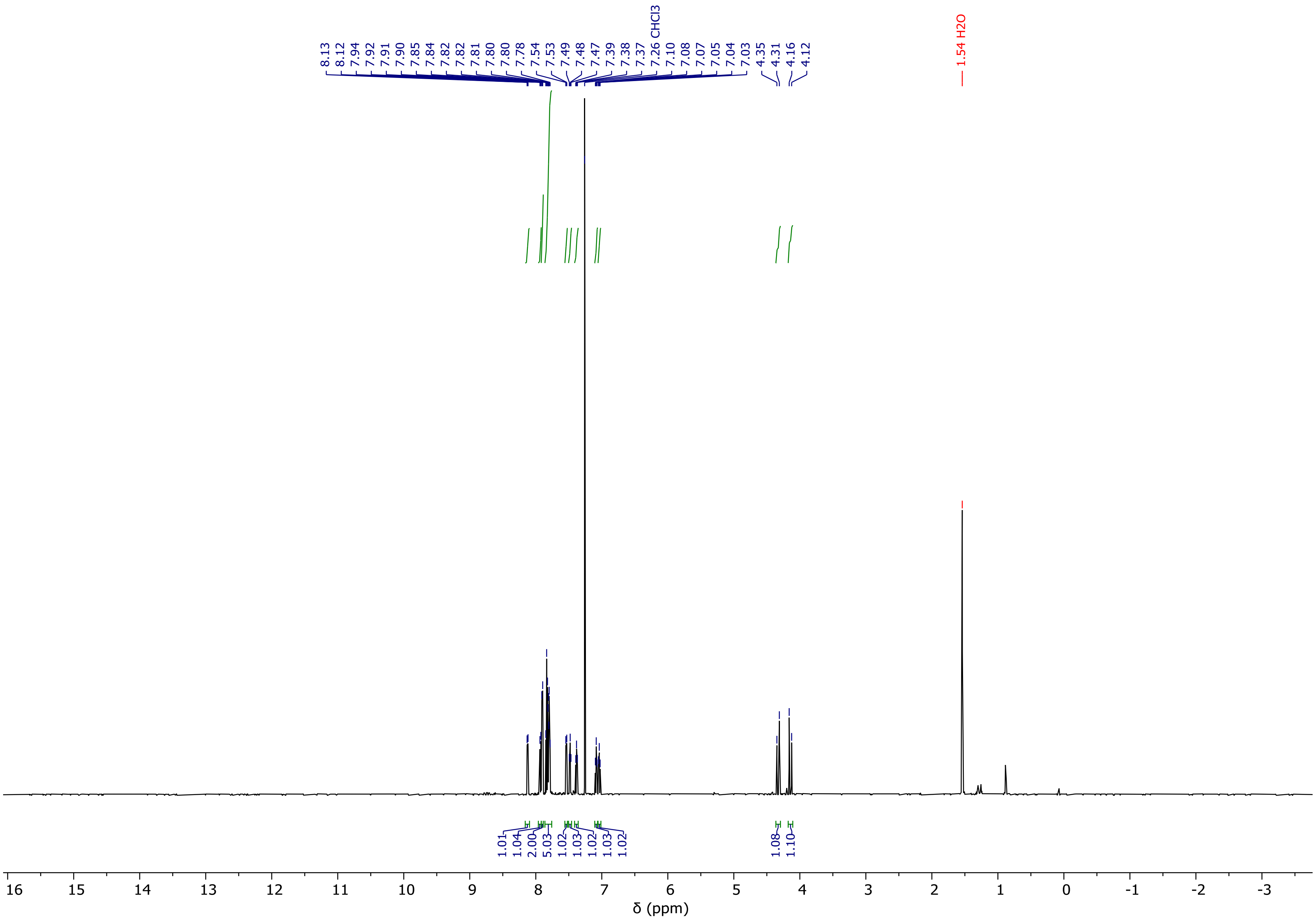

Figure S34. ^{1}H-NMR spectrum of fluoreno[6]helicene **4** (600 MHz, $CDCl_3$, 25 °C).

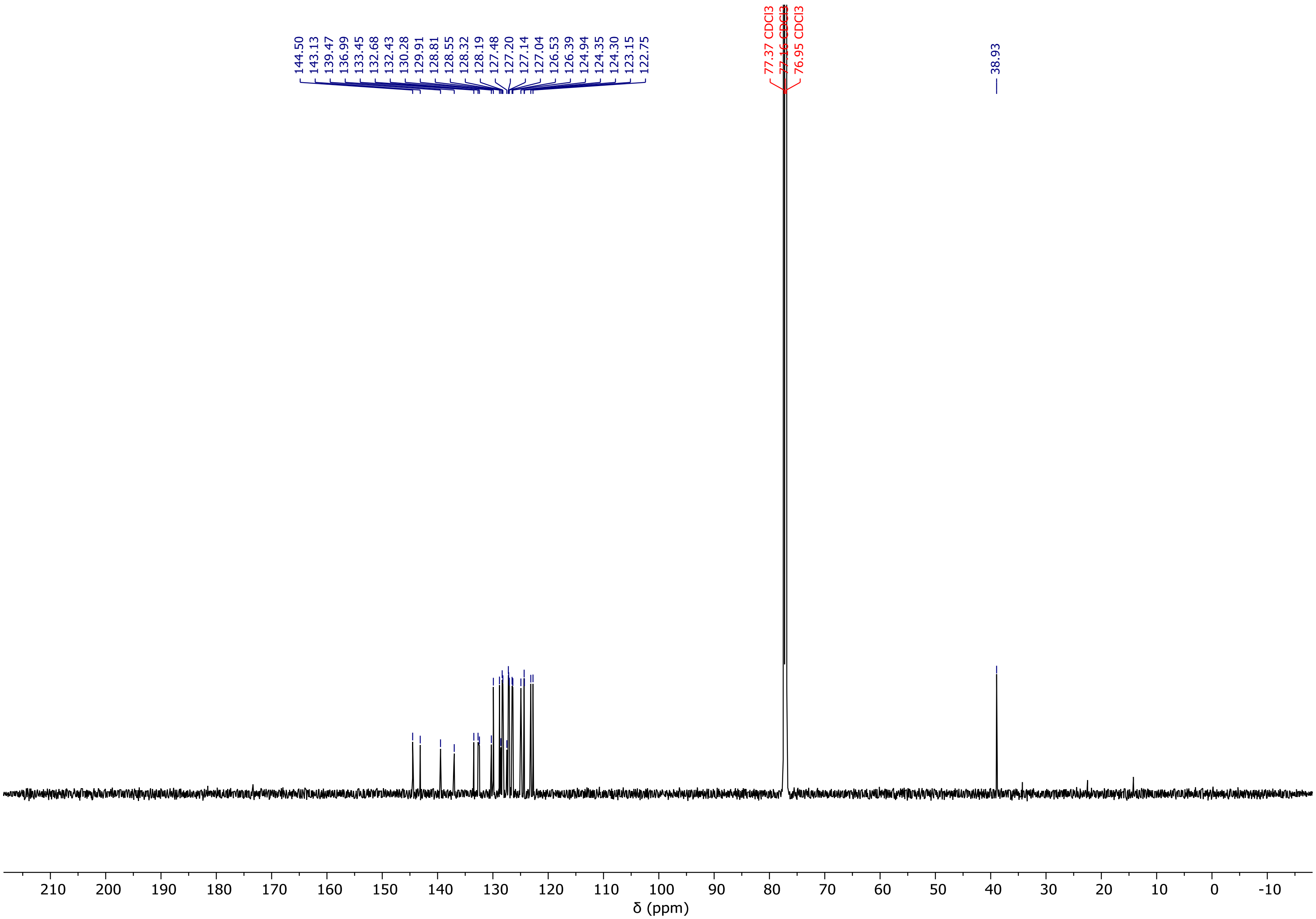

Figure S35. $^{13}C\{^{1}H\}$-NMR spectrum of hydrogenated fluoreno[6]helicene **4** (151 MHz, $CDCl_3$, 25 °C).

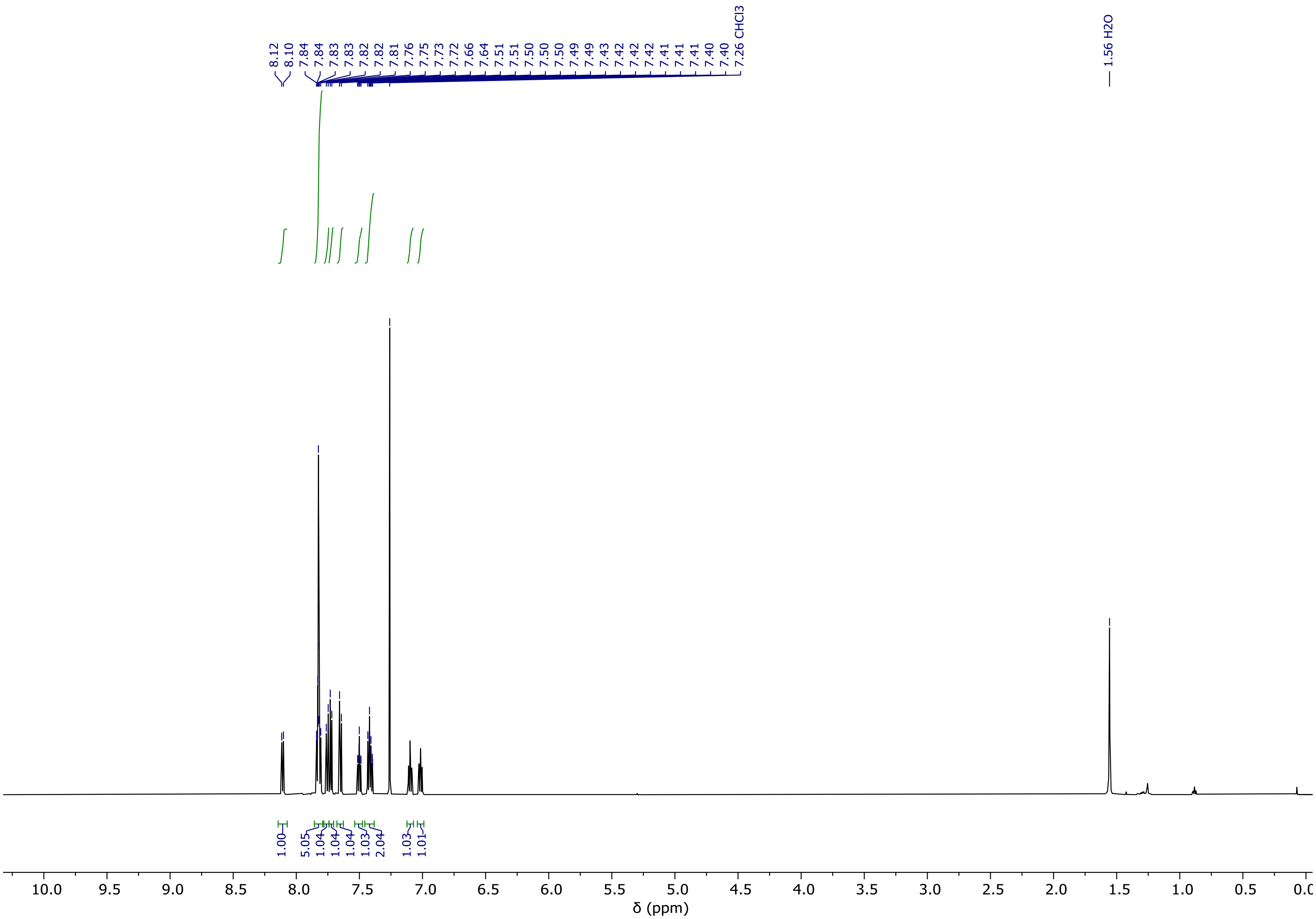

Figure S36. ^{1}H-NMR spectrum of fluoreno[6]helicenone **5** (600 MHz, $CDCl_3$, 25 °C).

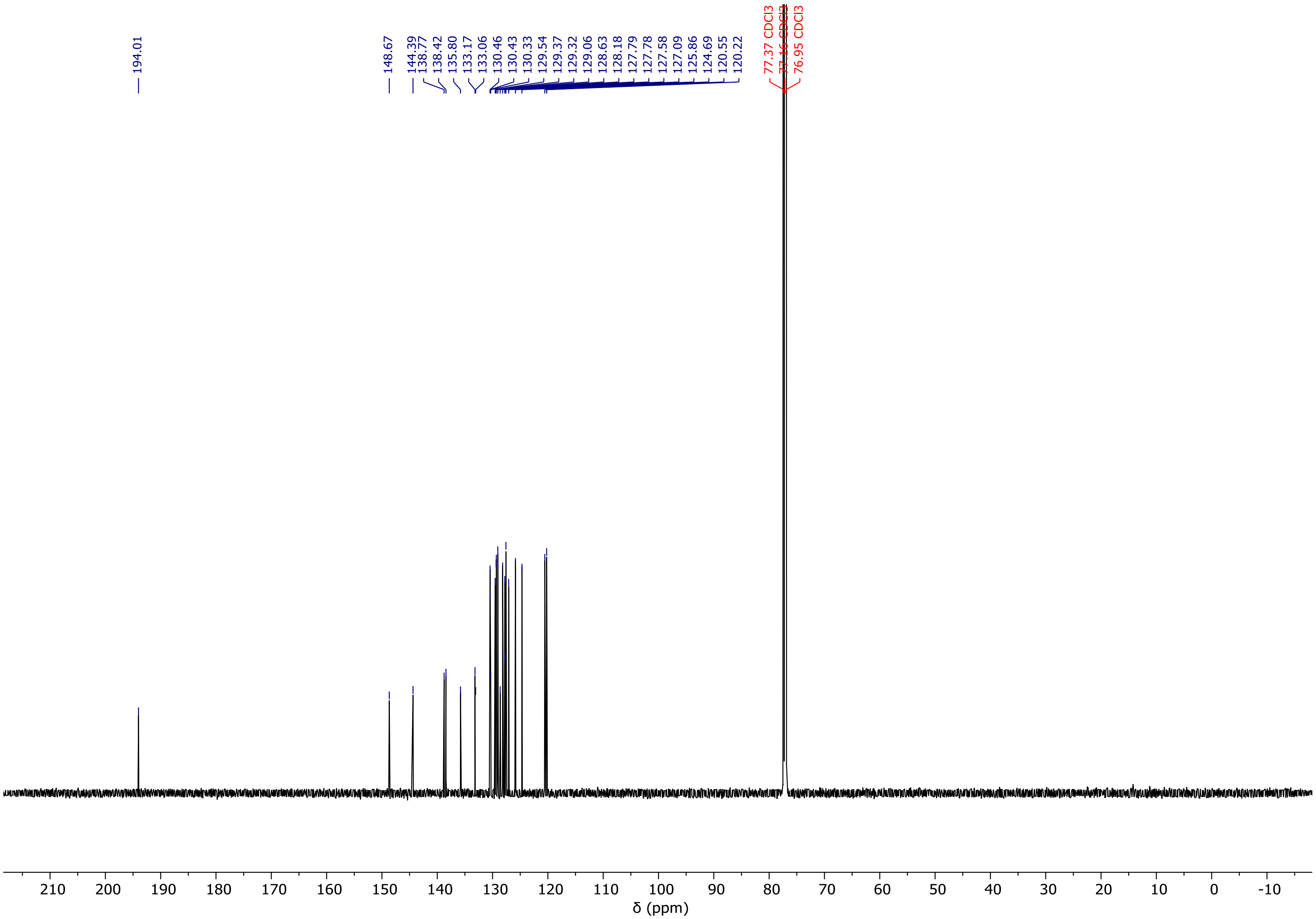

Figure S37. $^{13}C\{^{1}H\}$-NMR spectrum of fluoreno[6]helicenone **5** (151 MHz, $CDCl_3$, 25 °C).

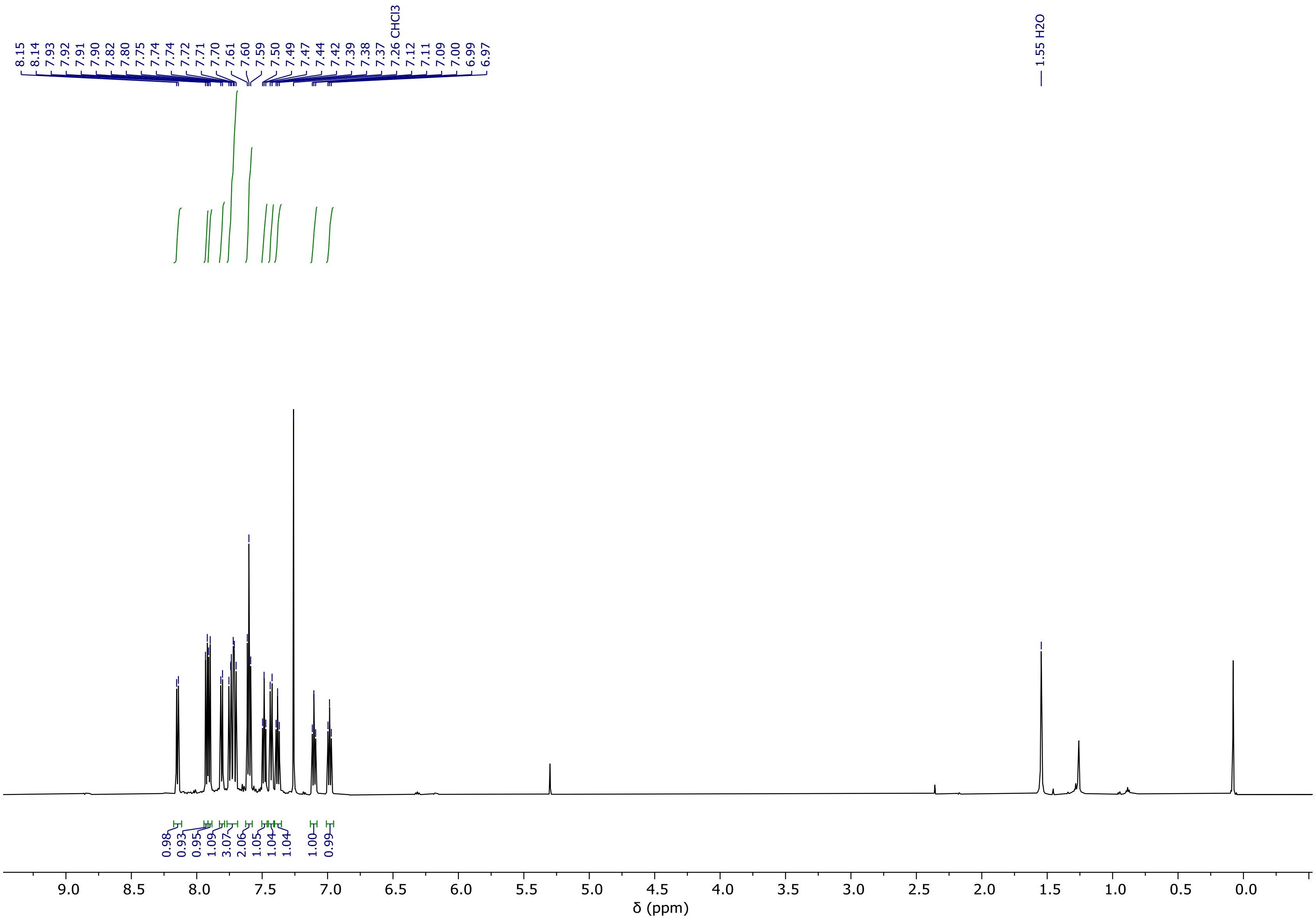

Figure S38. ^{1}H-NMR spectrum of thioketone **6** (600 MHz, $CDCl_3$, 25 °C).

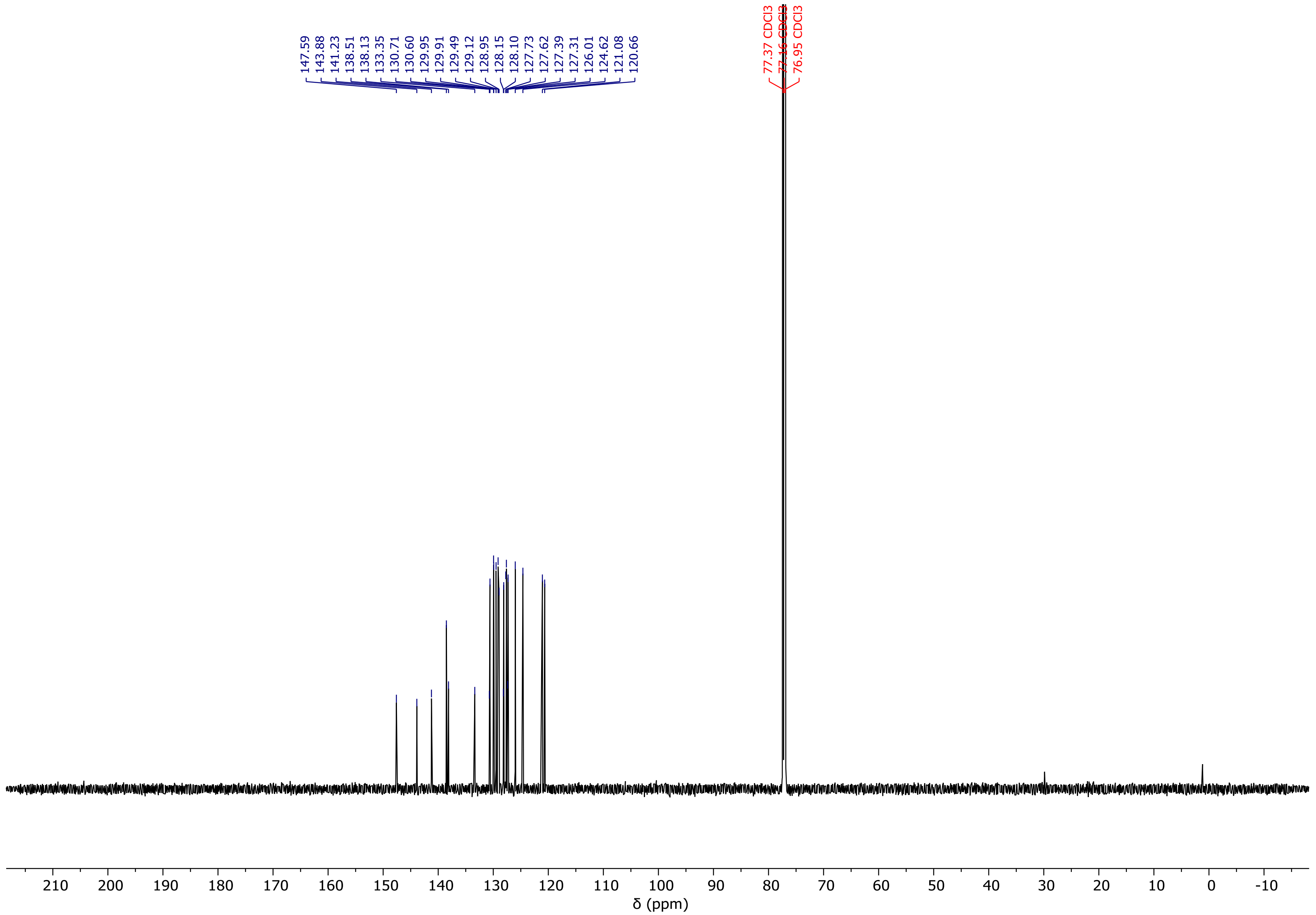

Figure S39. $^{13}C\{^{1}H\}$-NMR spectrum of thioketone **6** (151 MHz, $CDCl_3$, 25 °C).

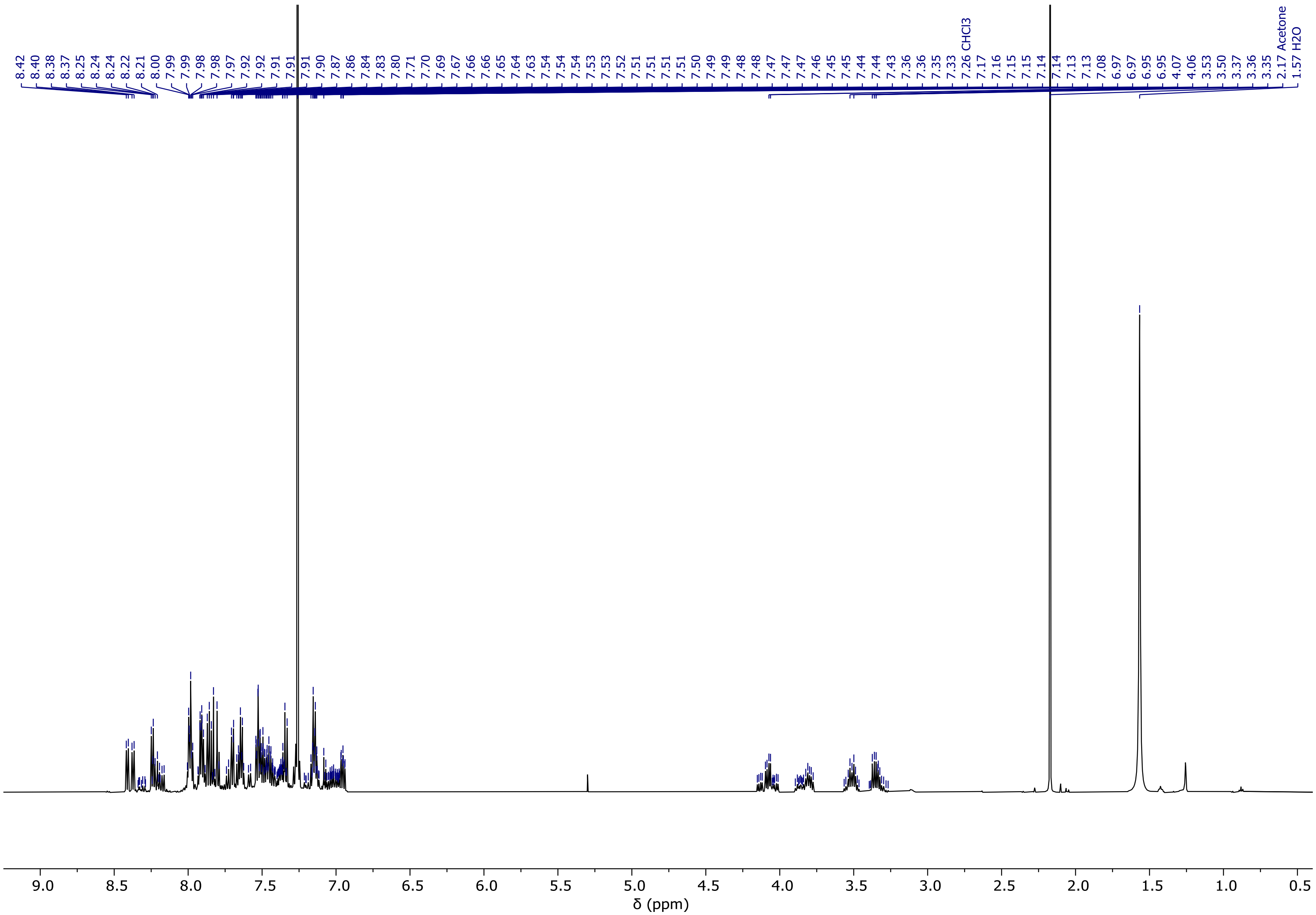

Figure S40. ^{1}H-NMR spectrum of **M1** as a mixture of stereoisomers (600 MHz, $CDCl_3$, 25 °C).

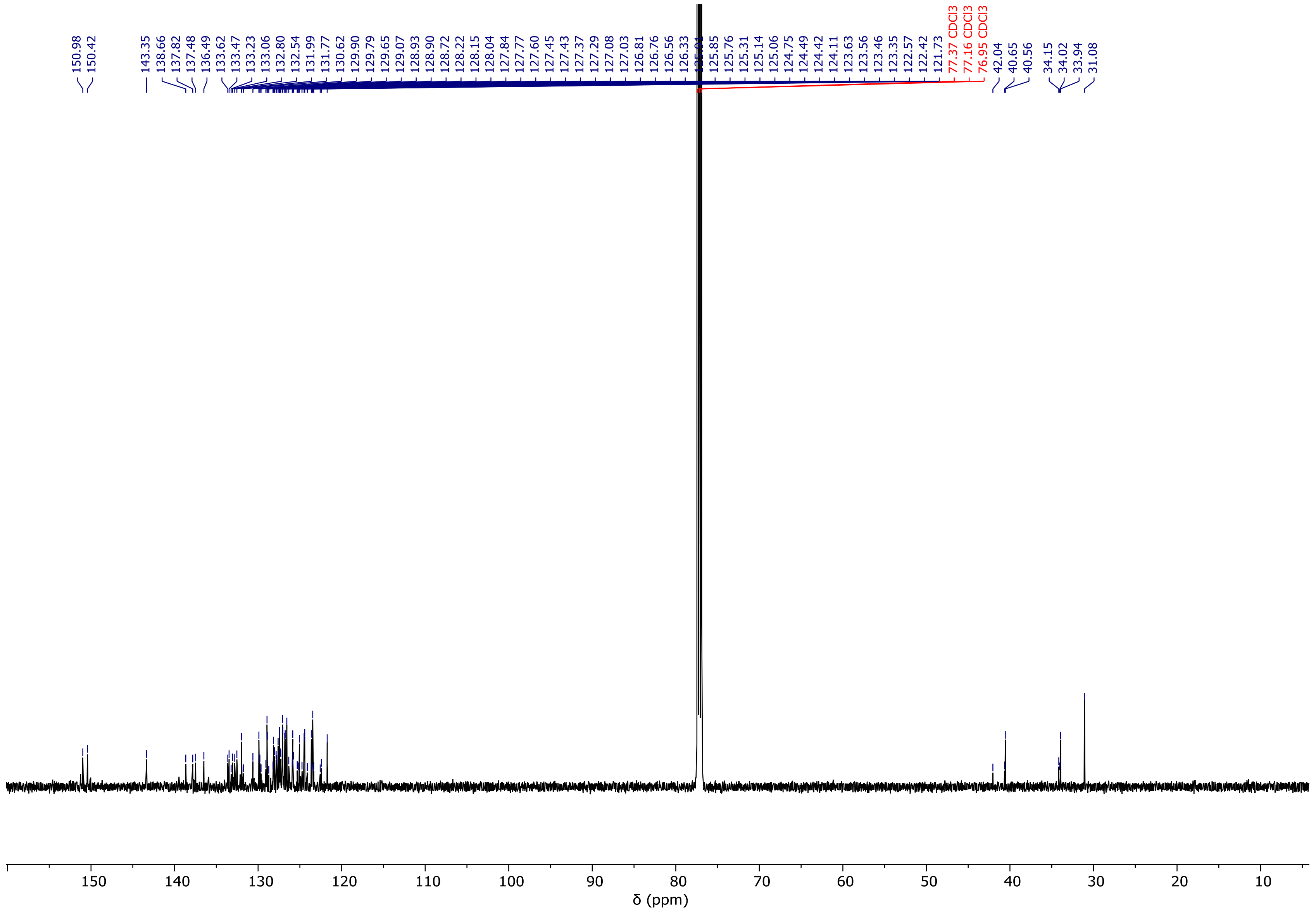

Figure S41. $^{13}C\{^{1}H\}$-NMR spectrum of **M1** as a mixture of stereoisomers (151 MHz, $CDCl_3$, 25 °C).

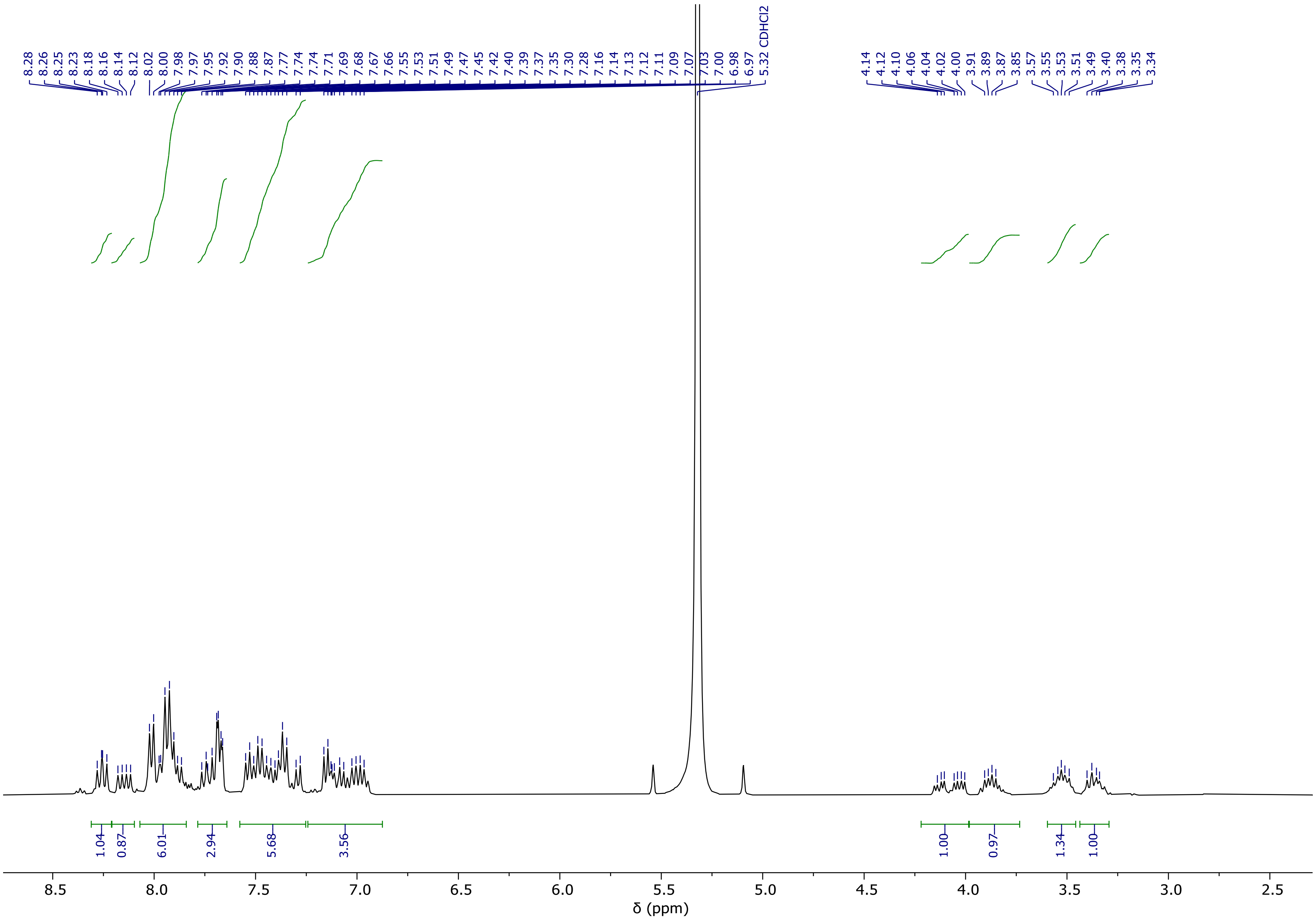

Figure S42. ^{1}H-NMR spectrum of a sample enriched in single configurational isomer at the double bond of **M1** composed of a mixture of its stable and metastable states (400 MHz, CD_2Cl_2, 25 °C).

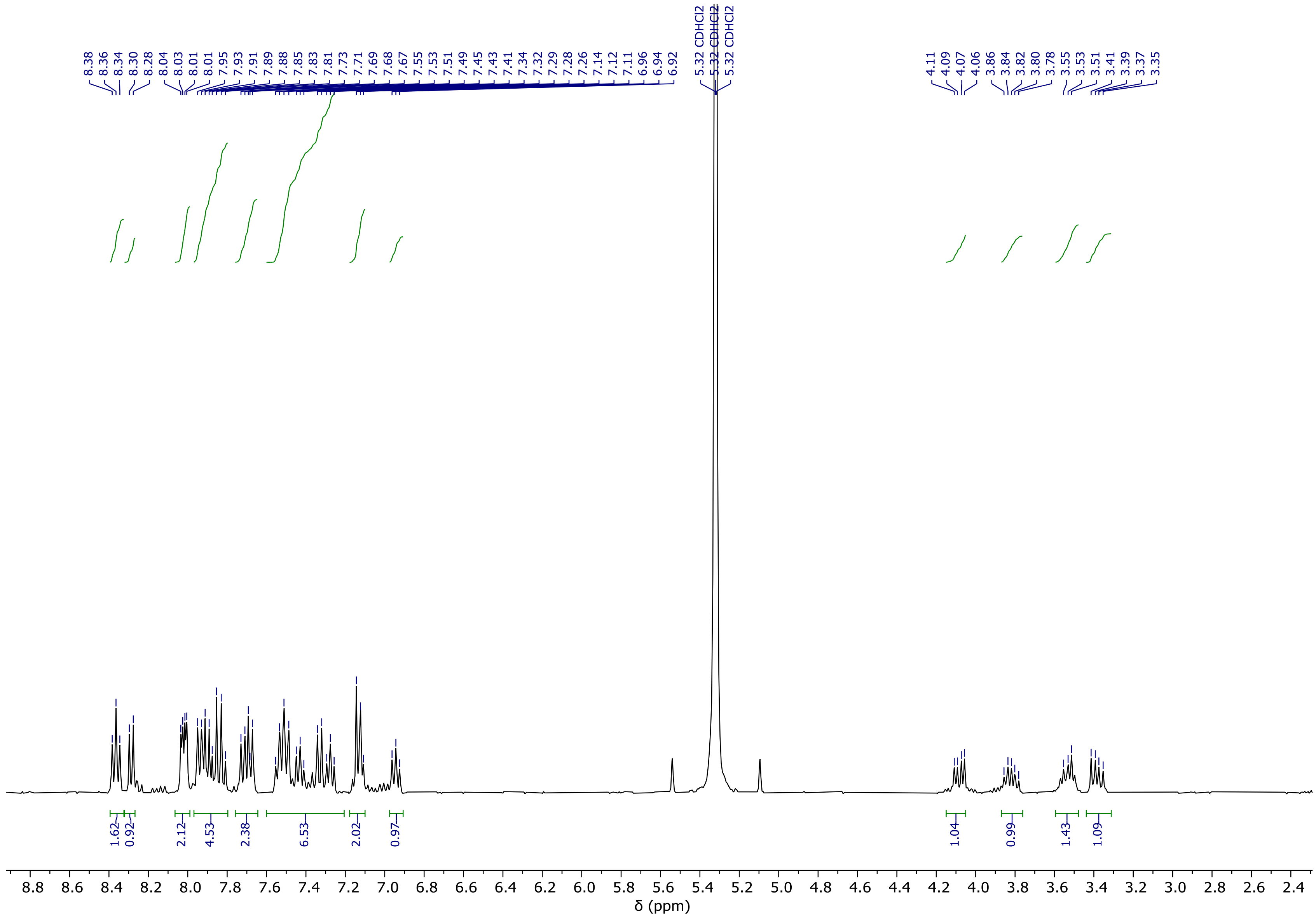

Figure S43. ^{1}H-NMR spectrum of a sample enriched in single configurational isomer at the double bond of **M1** composed of a mixture of its stable and metastable states (400 MHz, CD_2Cl_2, 25 °C).

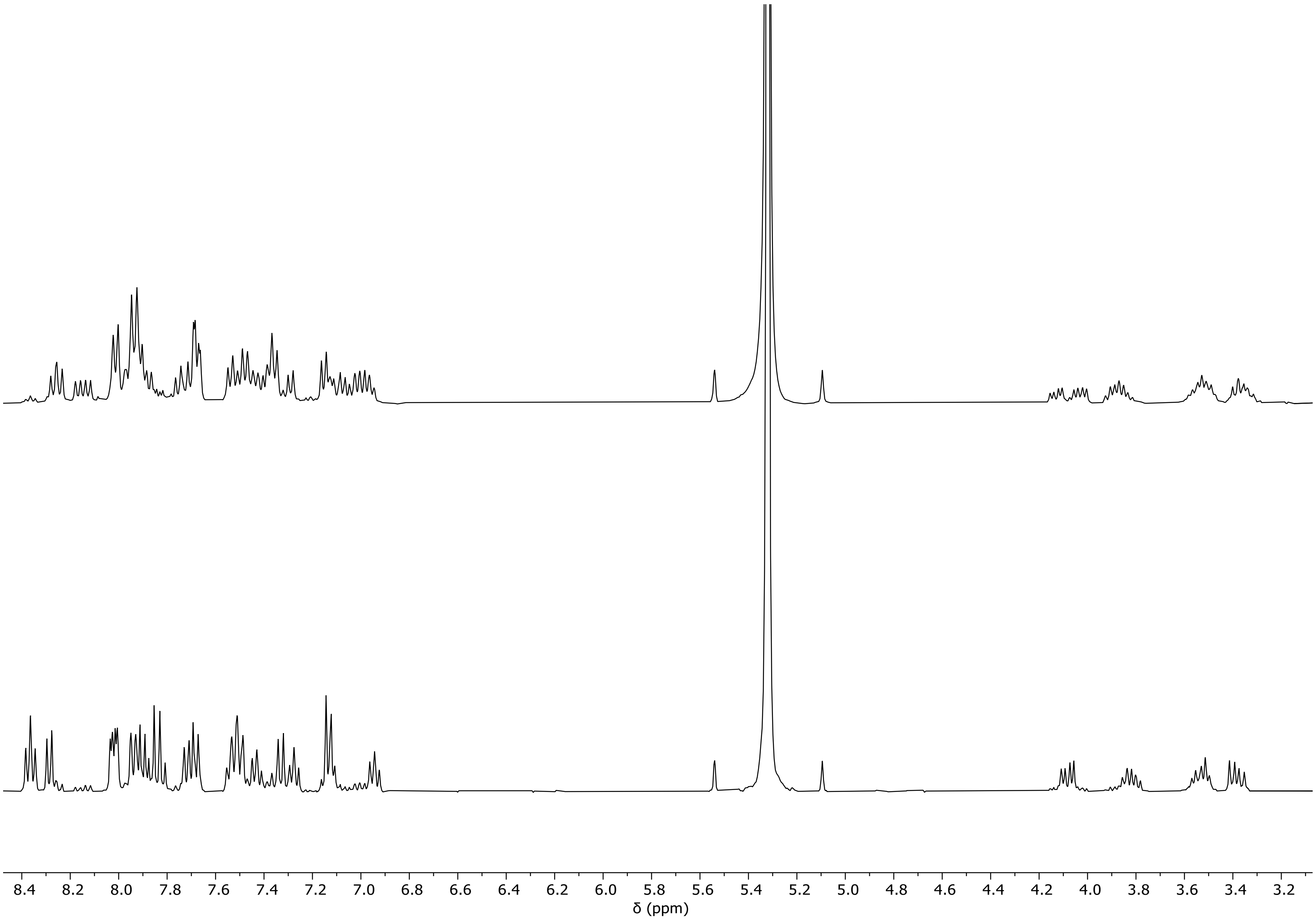

Figure S44. Stacked ^{1}H-NMR spectra of both configurational isomer at the double bond of **M1** (400 MHz, CD_2Cl_2, 25 °C).

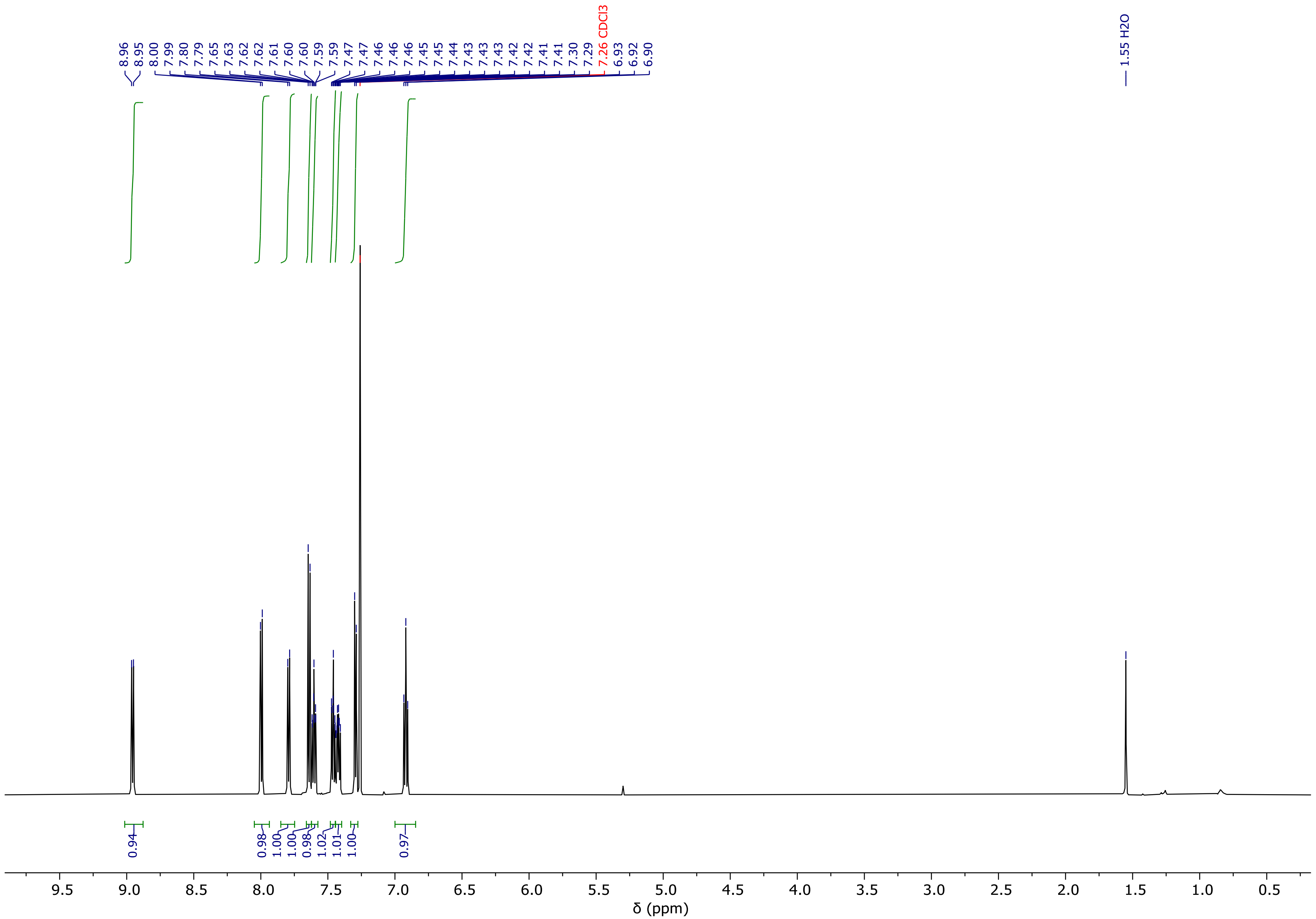

Figure S45. ^{1}H-NMR spectrum of 10-fluoro-11*H*-benzo[*a*]fluoren-11-one **7** (600 MHz, CD_2Cl_2, 25 °C).

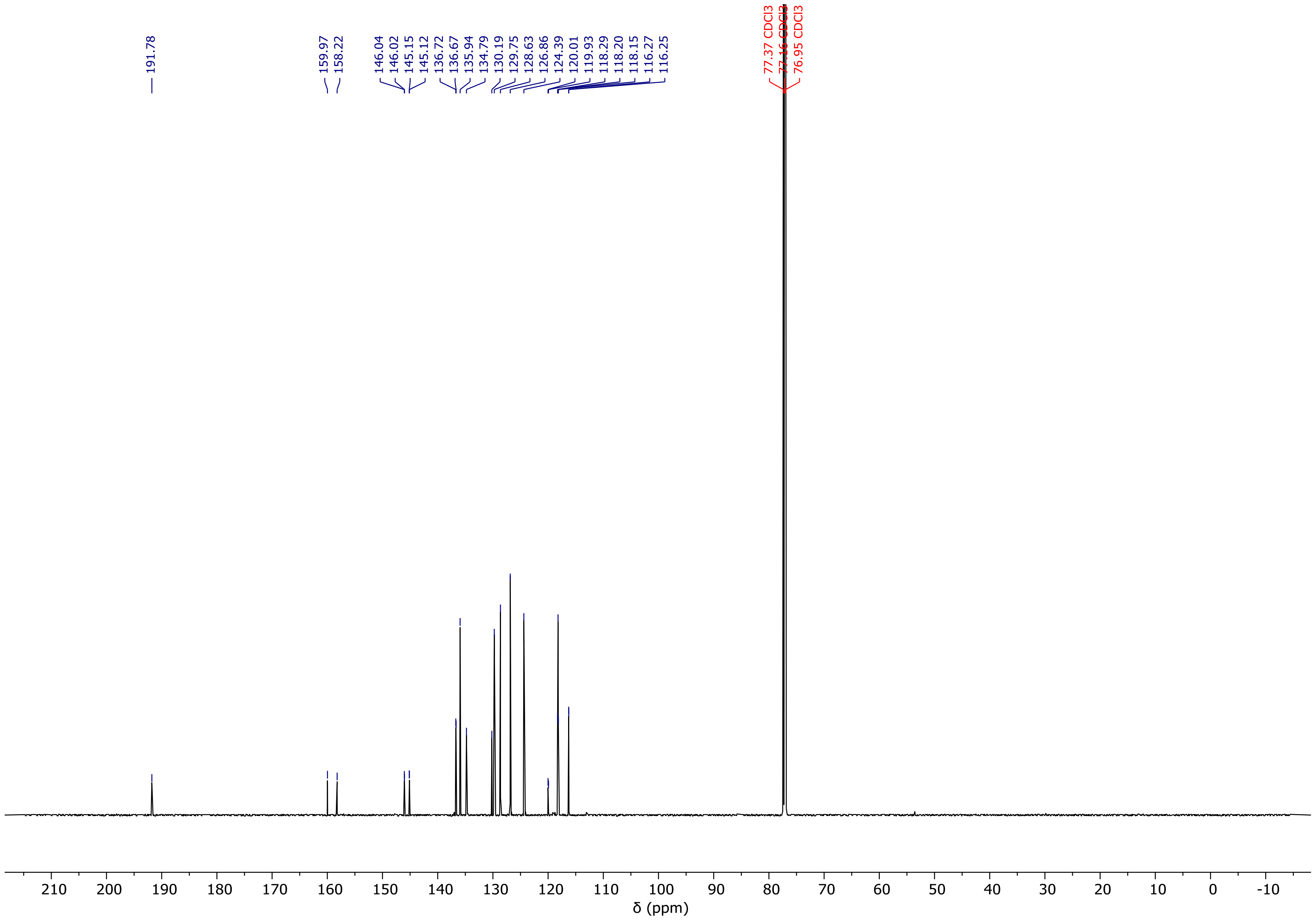

Figure S46. $^{13}C\{^1H\}$-NMR spectrum of 10-fluoro-11*H*-benzo[*a*]fluoren-11-one **7** (151 MHz, $CDCl_3$, 25 °C).

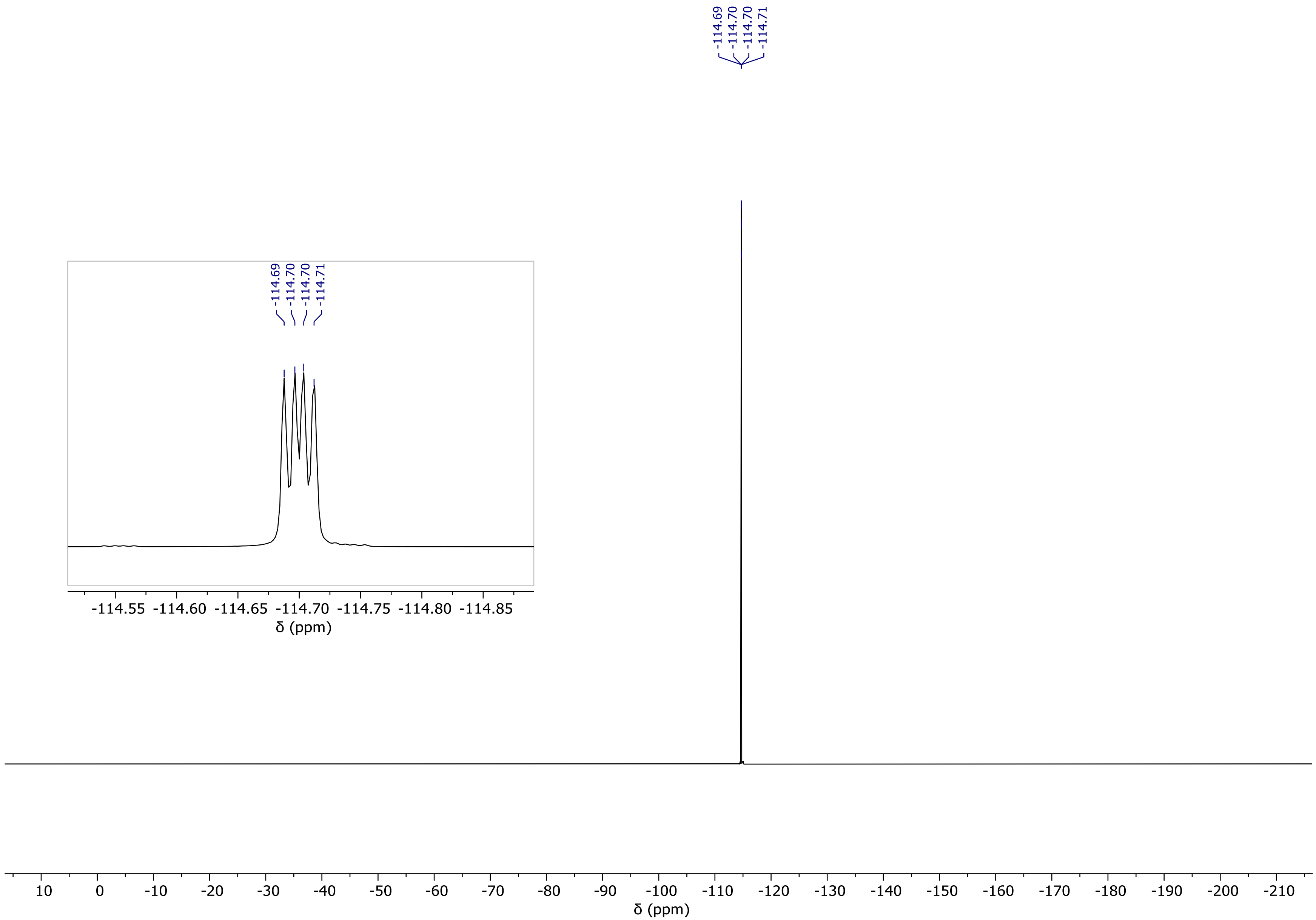

Figure S47. ^{19}F spectrum of 10-fluoro-11*H*-benzo[*a*]fluoren-11-one **7** (565 MHz, $CDCl_3$, 25 °C).

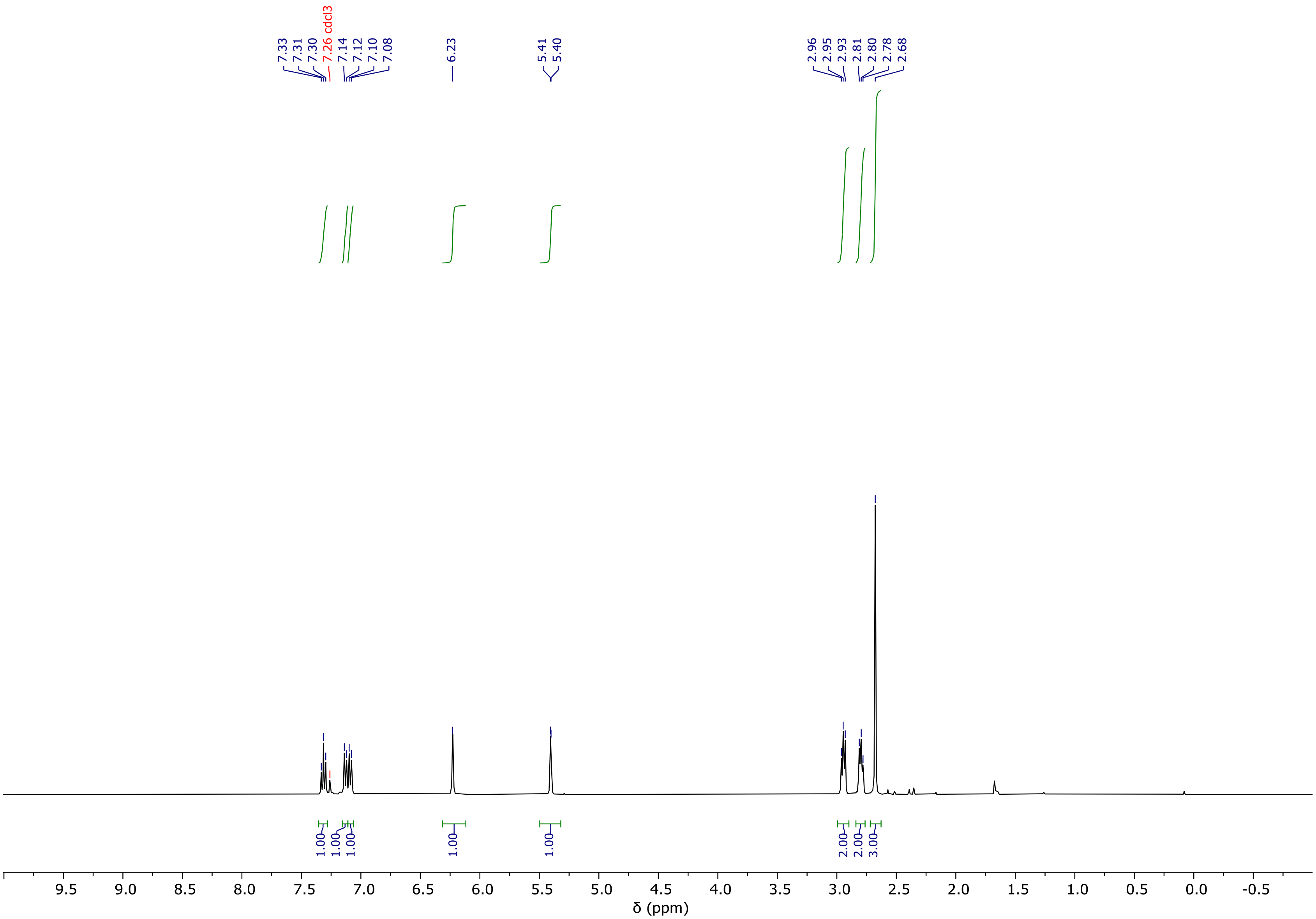

Figure S48. ^{1}H-NMR spectrum of 8-methyl-2-methylene-1-tetralone (**8**) (400 MHz, $CDCl_3$, 25 °C).

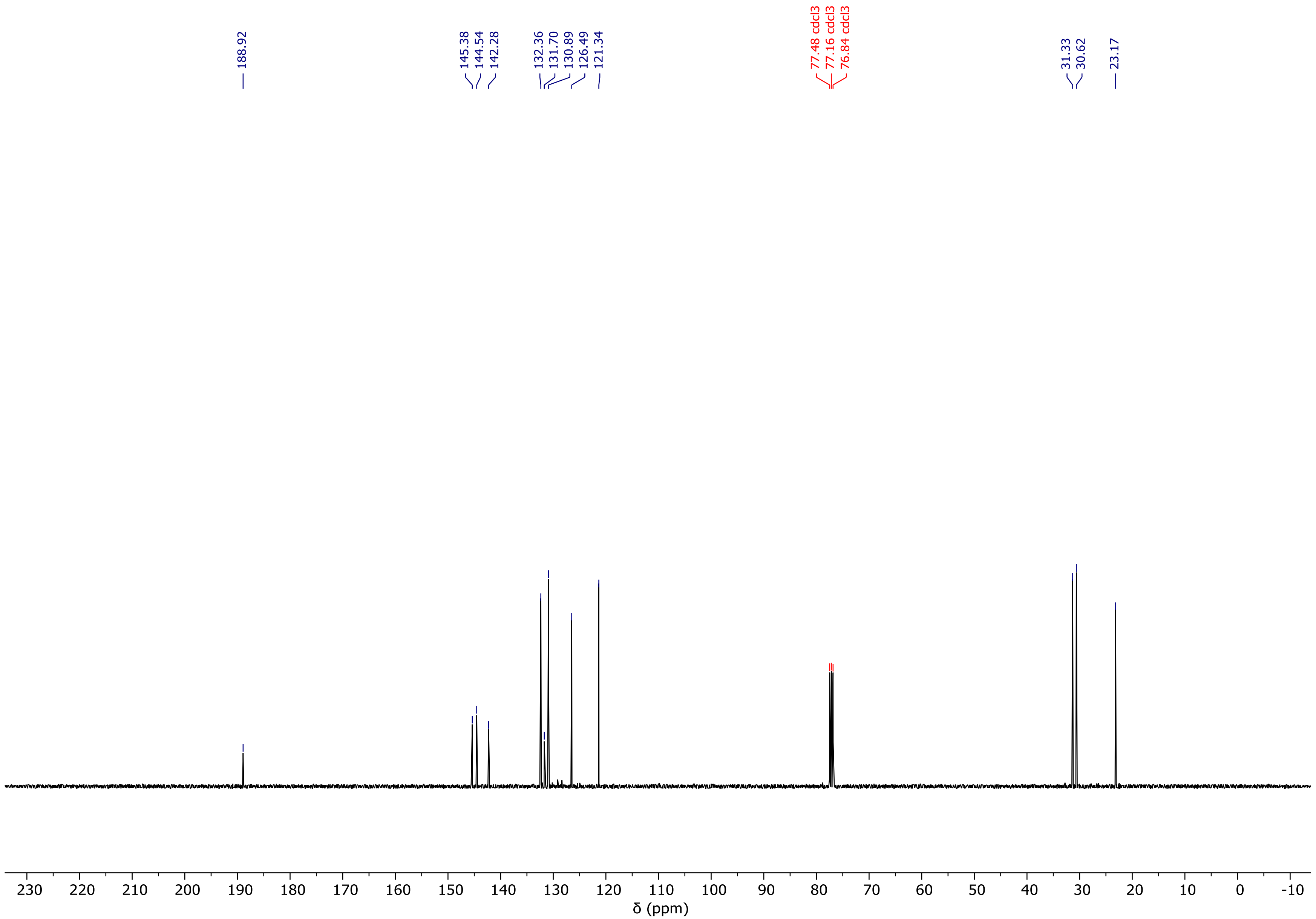

Figure S49. $^{13}C\{^{1}H\}$-NMR spectrum of 8-methyl-2-methylene-1-tetralone (**8**) (101 MHz, $CDCl_3$, 25 °C).

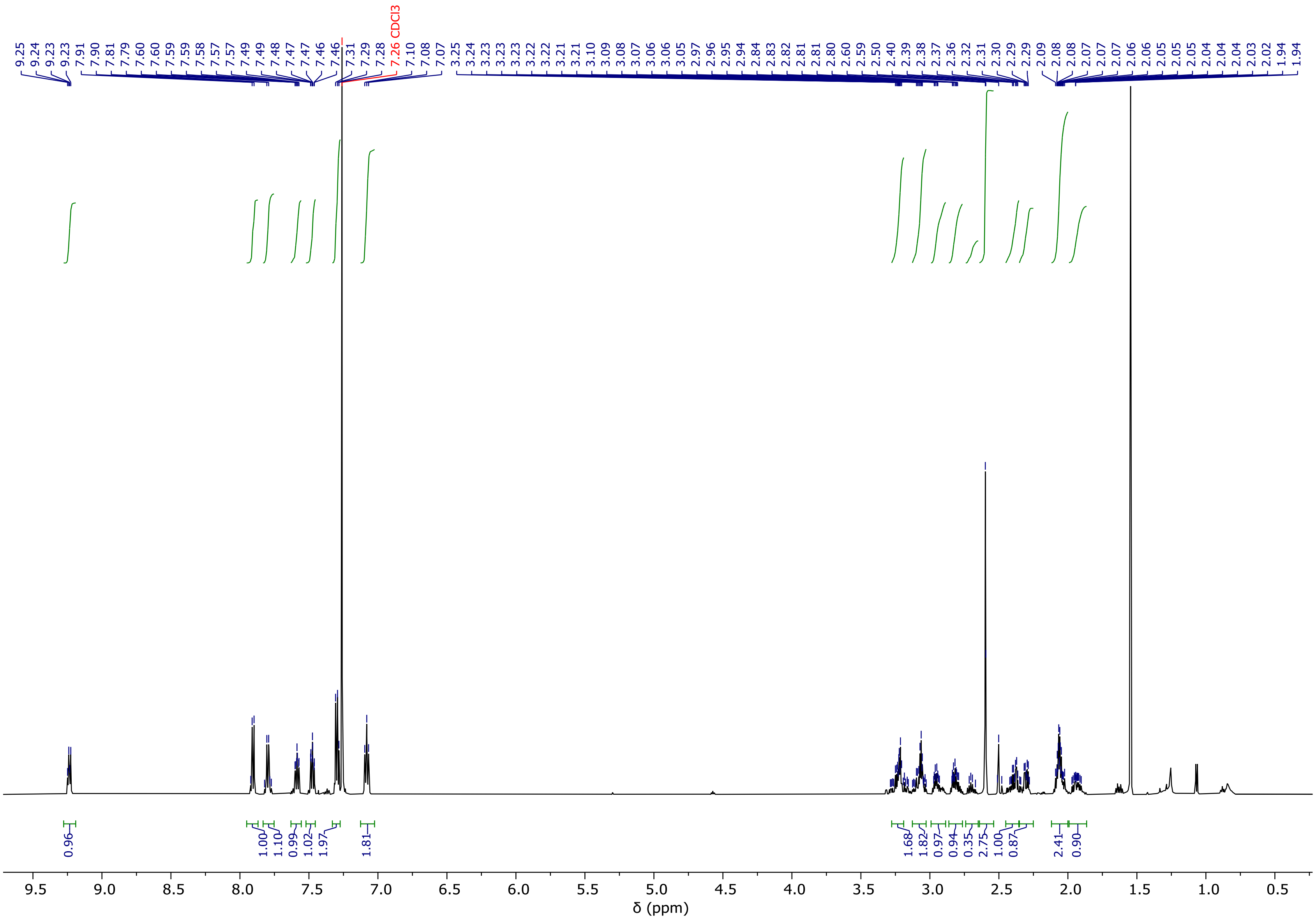

Figure S50. ^{1}H-NMR spectrum of 1,5-diketone **9** (600 MHz, $CDCl_3$, 25 °C).

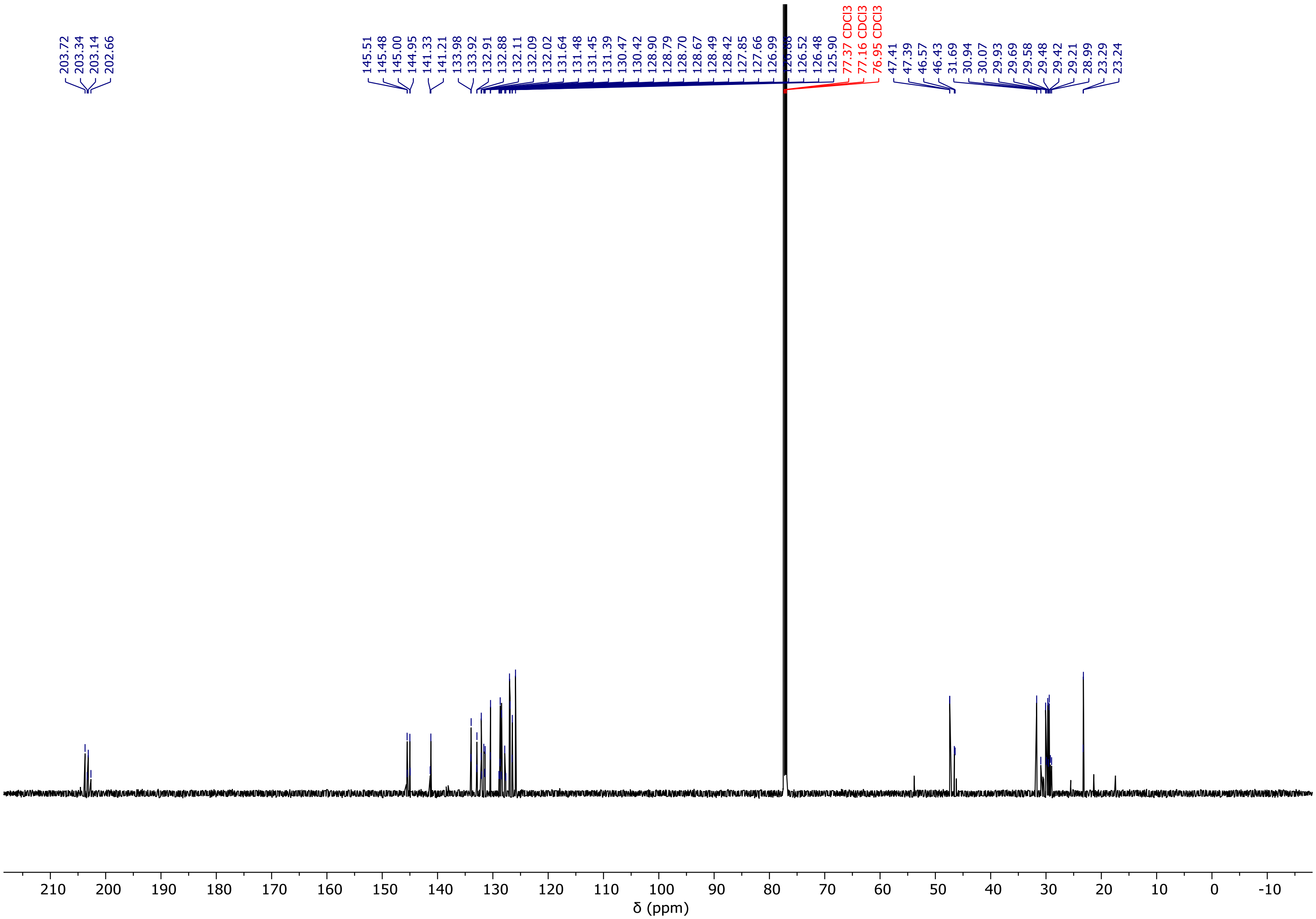

Figure S51. $^{13}C\{^{1}H\}$-NMR spectrum of 1,5-diketone **9** (151 MHz, $CDCl_3$, 25 °C).

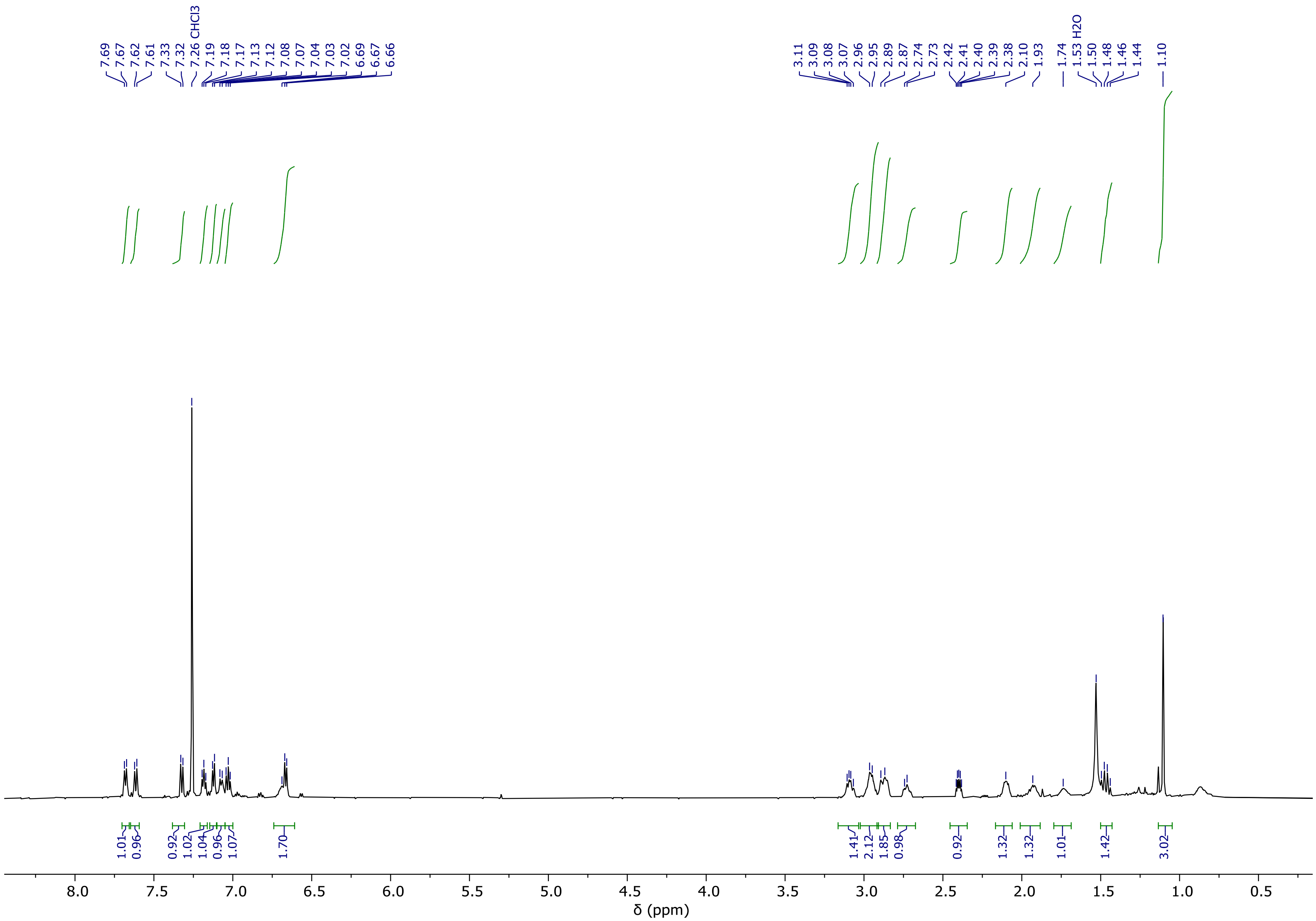

Figure S52. ^{1}H-NMR spectrum of hydrogenated methylated fluoreno[6]helicene **10** (600 MHz, $CDCl_3$, 25 °C).

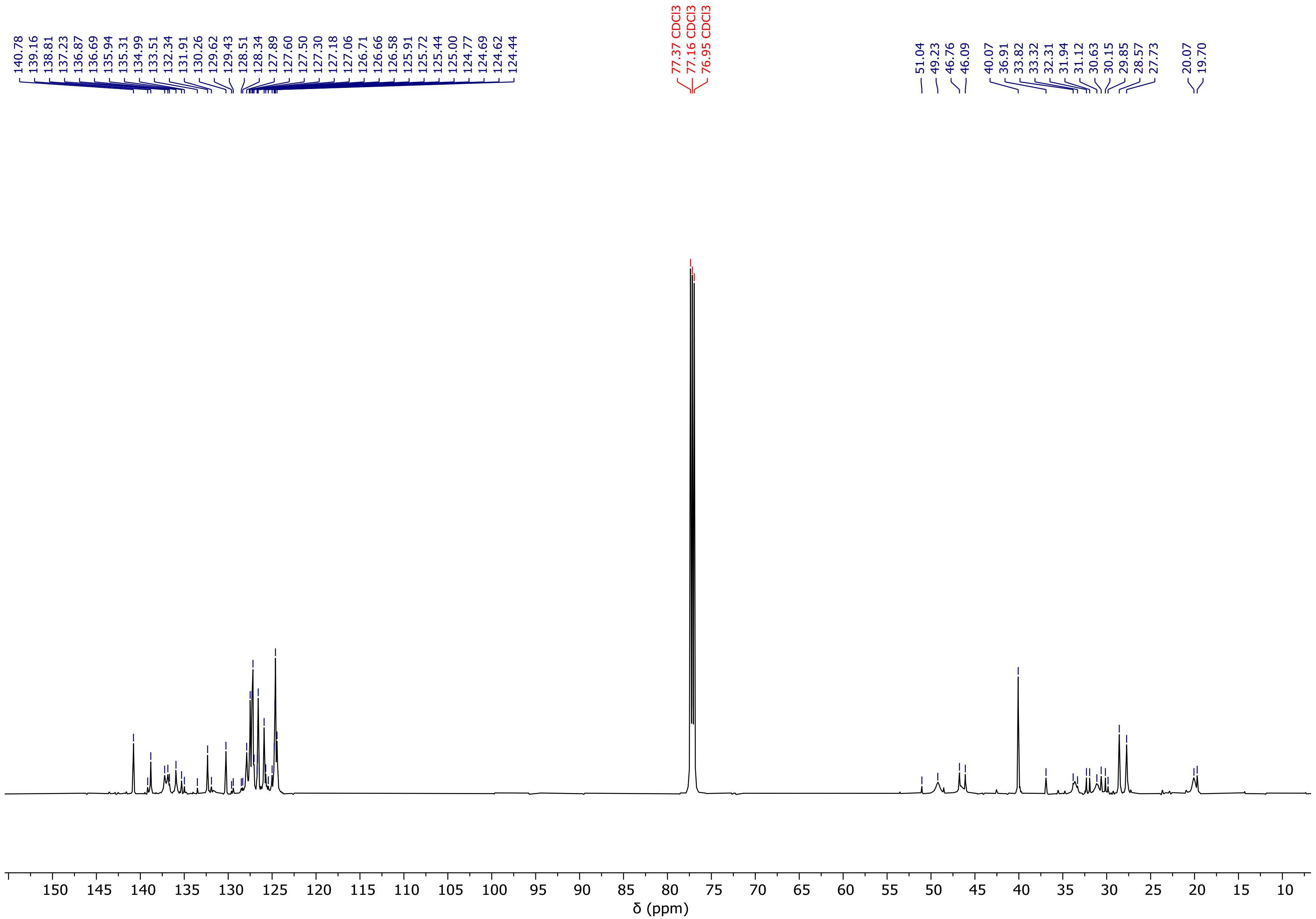

Figure S53. $^{13}C\{^{1}H\}$-NMR spectrum of hydrogenated methylated fluoreno[6]helicene **10** (151 MHz, $CDCl_3$, 25 °C).

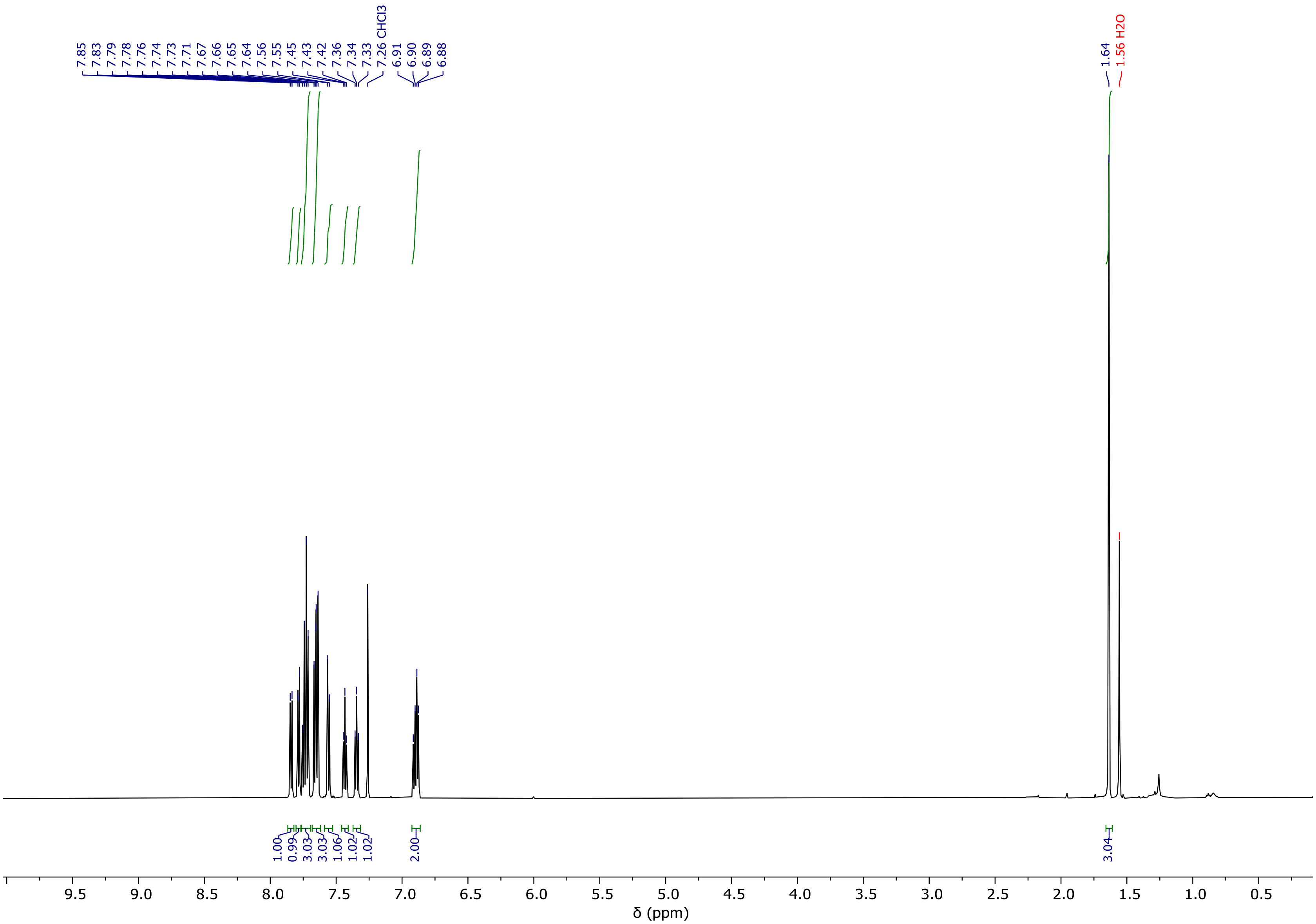

Figure S54. ^{1}H-NMR spectrum of methylated fluoreno[6]helicenone **11** (600 MHz, $CDCl_3$, 25 °C).

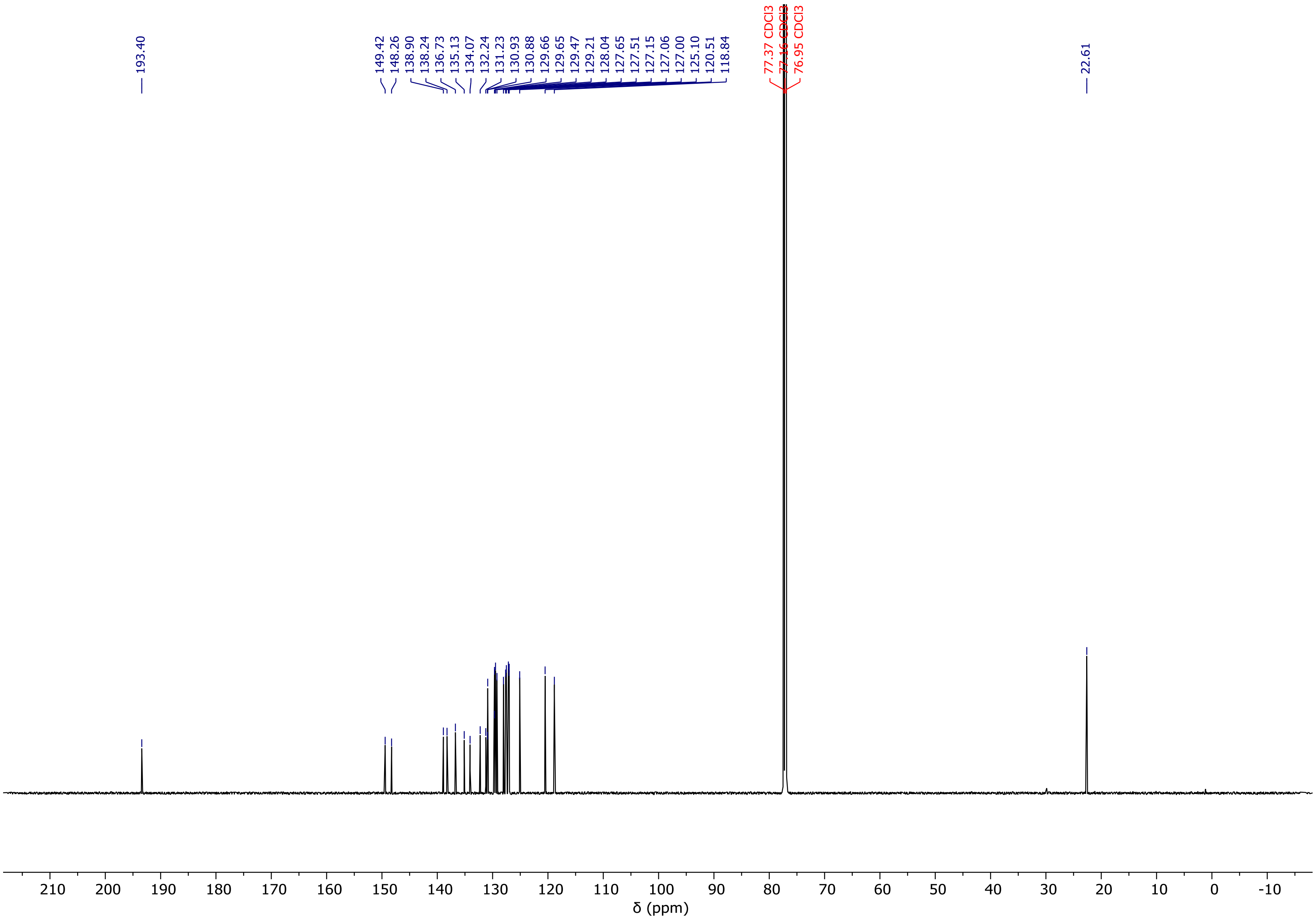

Figure S55. $^{13}C\{^{1}H\}$-NMR spectrum of methylated fluoreno[6]helicenone **11** (151 MHz, $CDCl_3$, 25 °C).

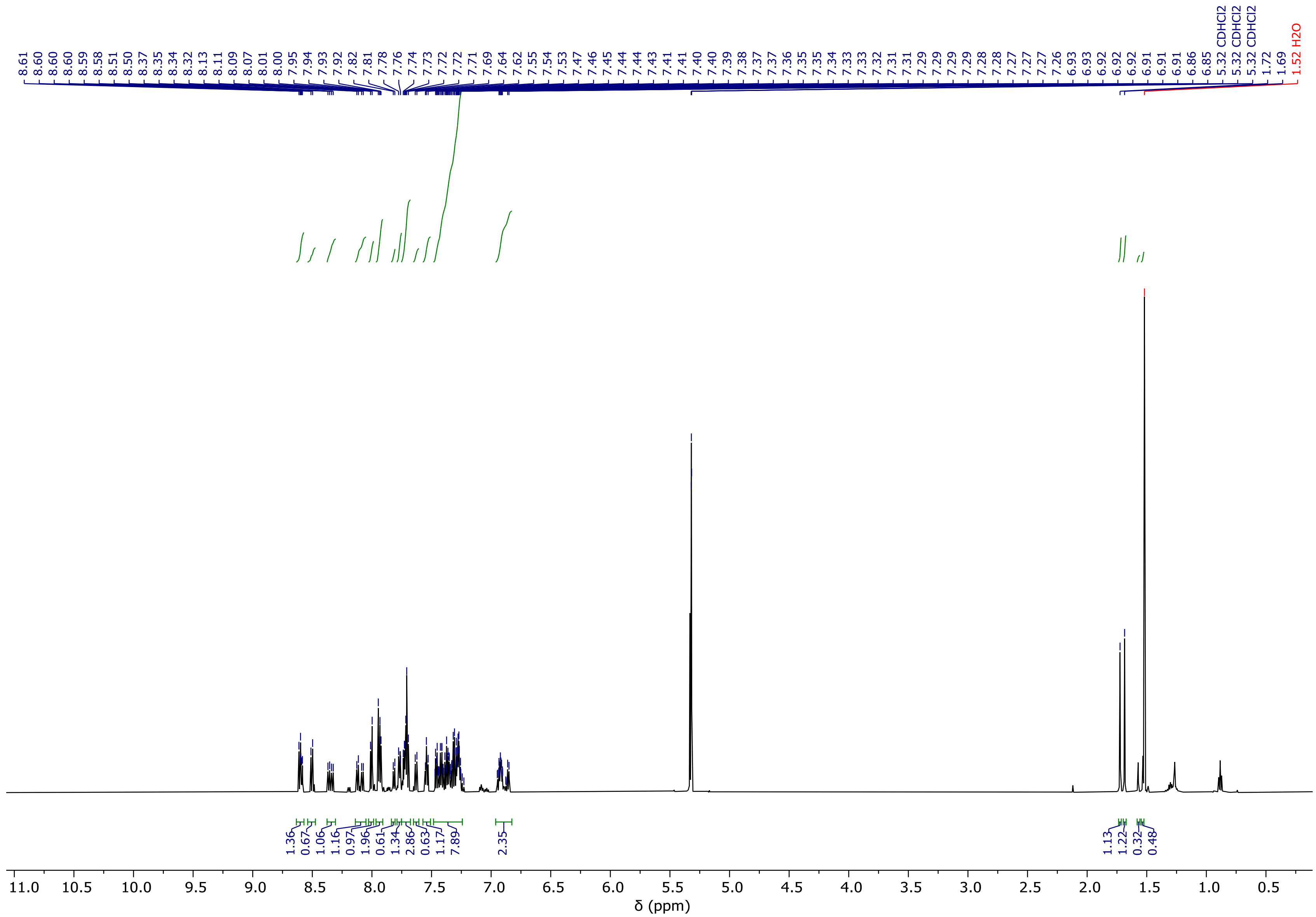

Figure S56. ^{1}H-NMR spectrum of **M2** as a mixture of stereoisomers (600 MHz, CD_2Cl_2, 25 °C).

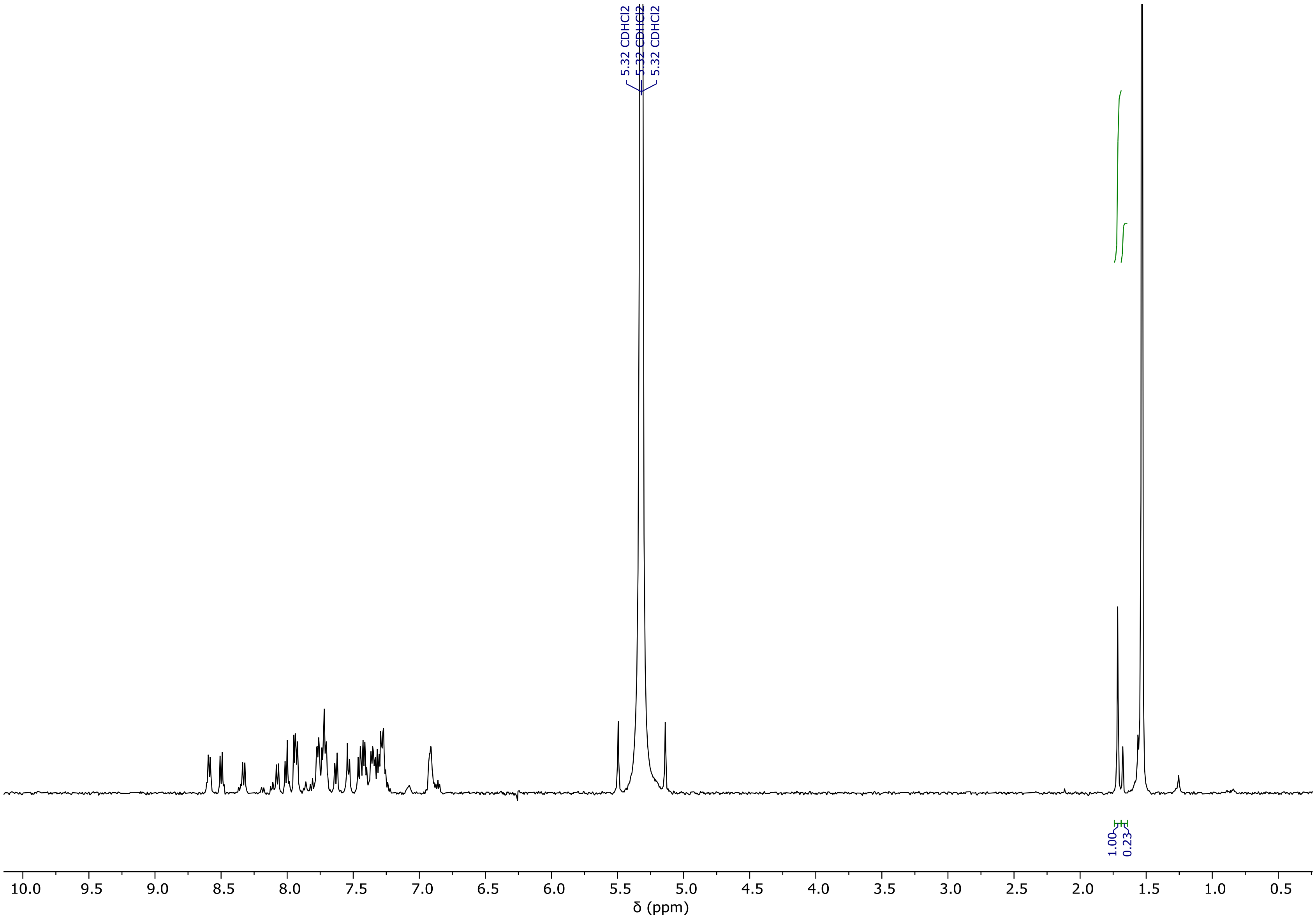

Figure S57. ^{1}H-NMR spectrum of **M2** enriched in one of its stereoisomers by precipitation in MeOH (500 MHz, CD_2Cl_2, 15 °C).

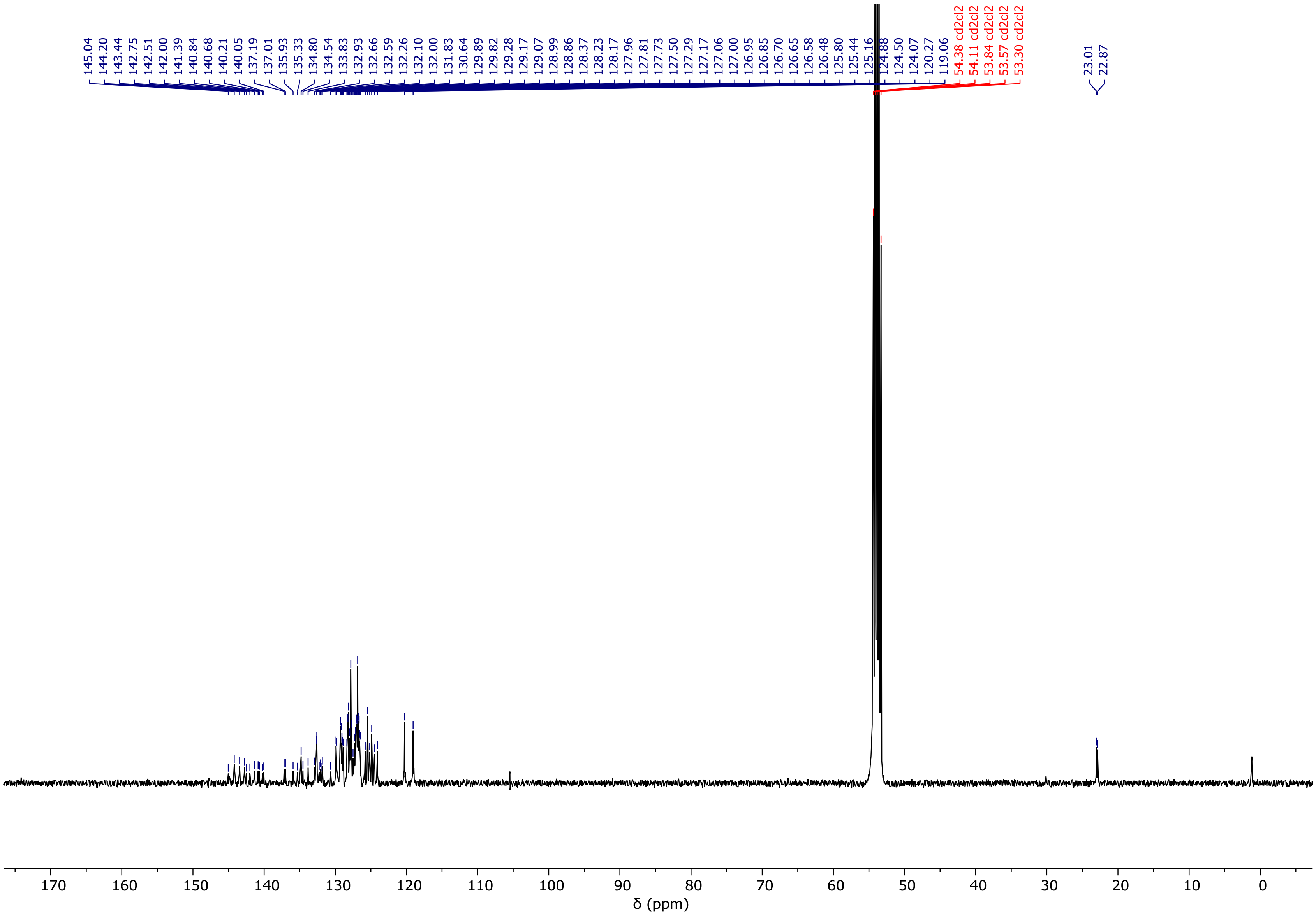

Figure S58. $^{13}C\{^1H\}$-NMR spectrum of **M2** as a mixture of stereoisomers (101 MHz, CD_2Cl_2, 25 °C).

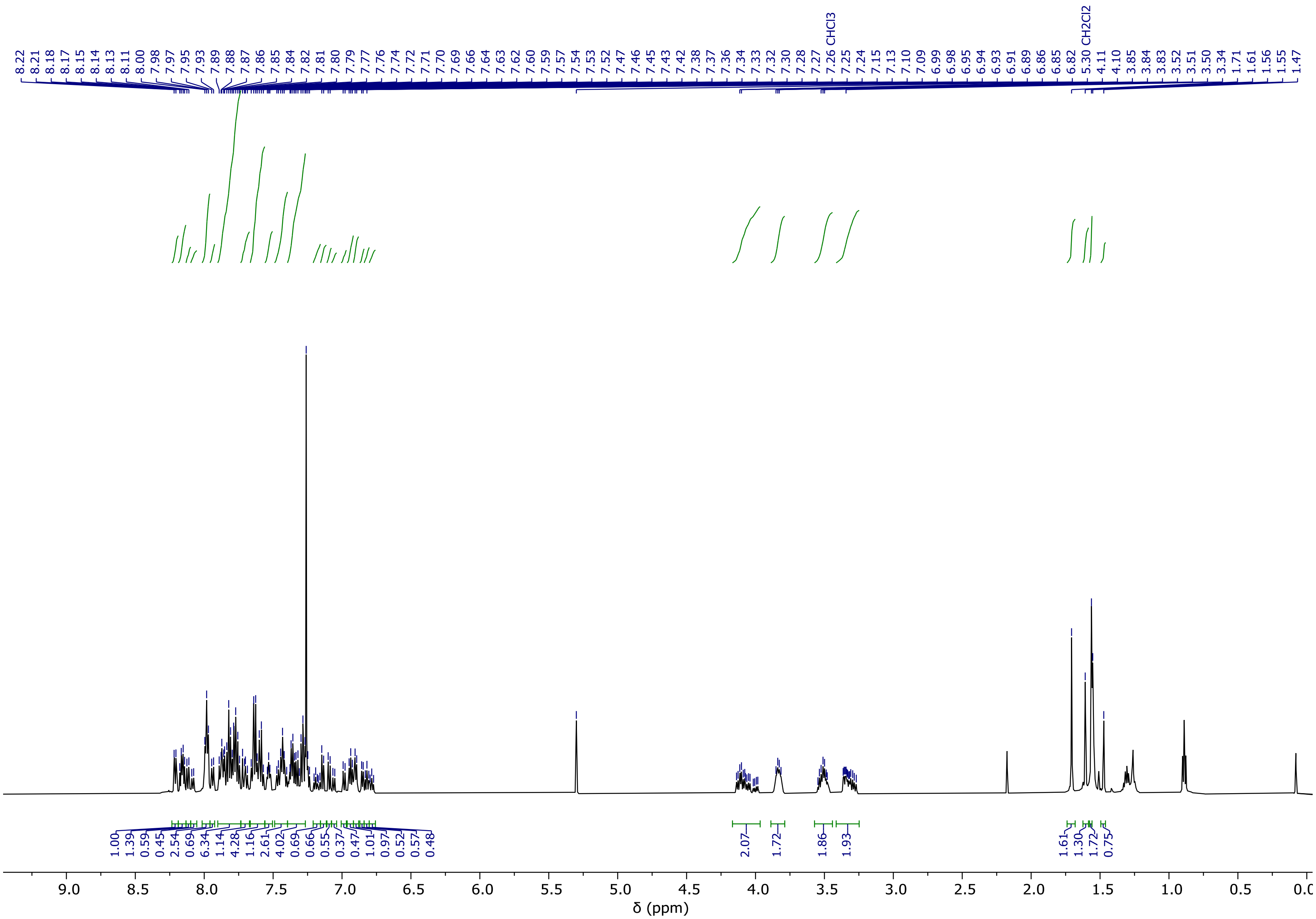

Figure S59. ^{1}H-NMR spectrum of **M3** as a mixture of stereoisomers (600 MHz, $CDCl_3$, 25 °C).

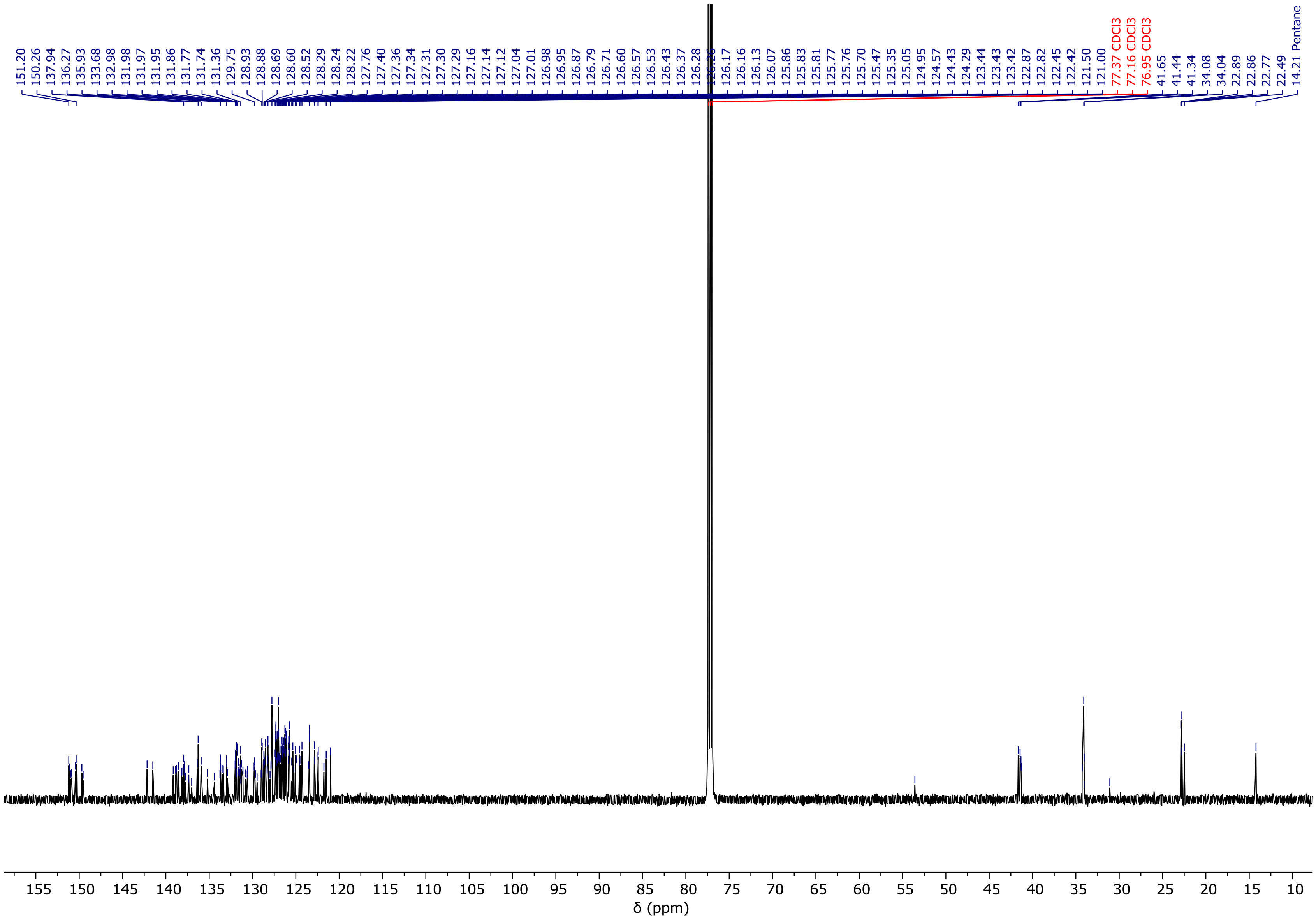

Figure S60. $^{13}C\{^{1}H\}$-NMR spectrum of **M3** as a mixture of stereoisomers (151 MHz, $CDCl_3$, 25 °C).

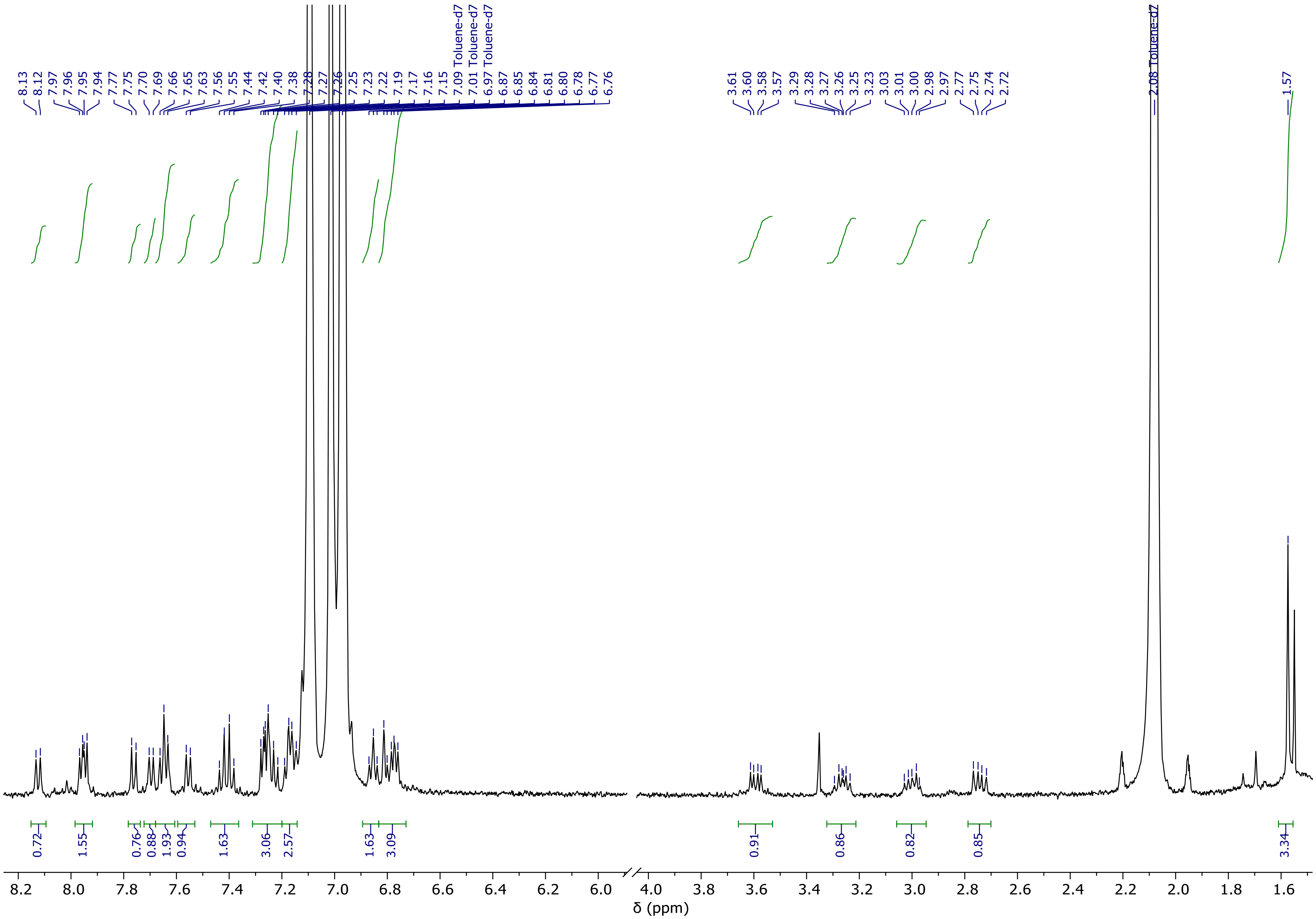

Figure S61. ^{1}H-NMR spectrum of sample enriched in a single metastable isomer of **M3** (500 MHz, Toluene-d8, 15 °C).

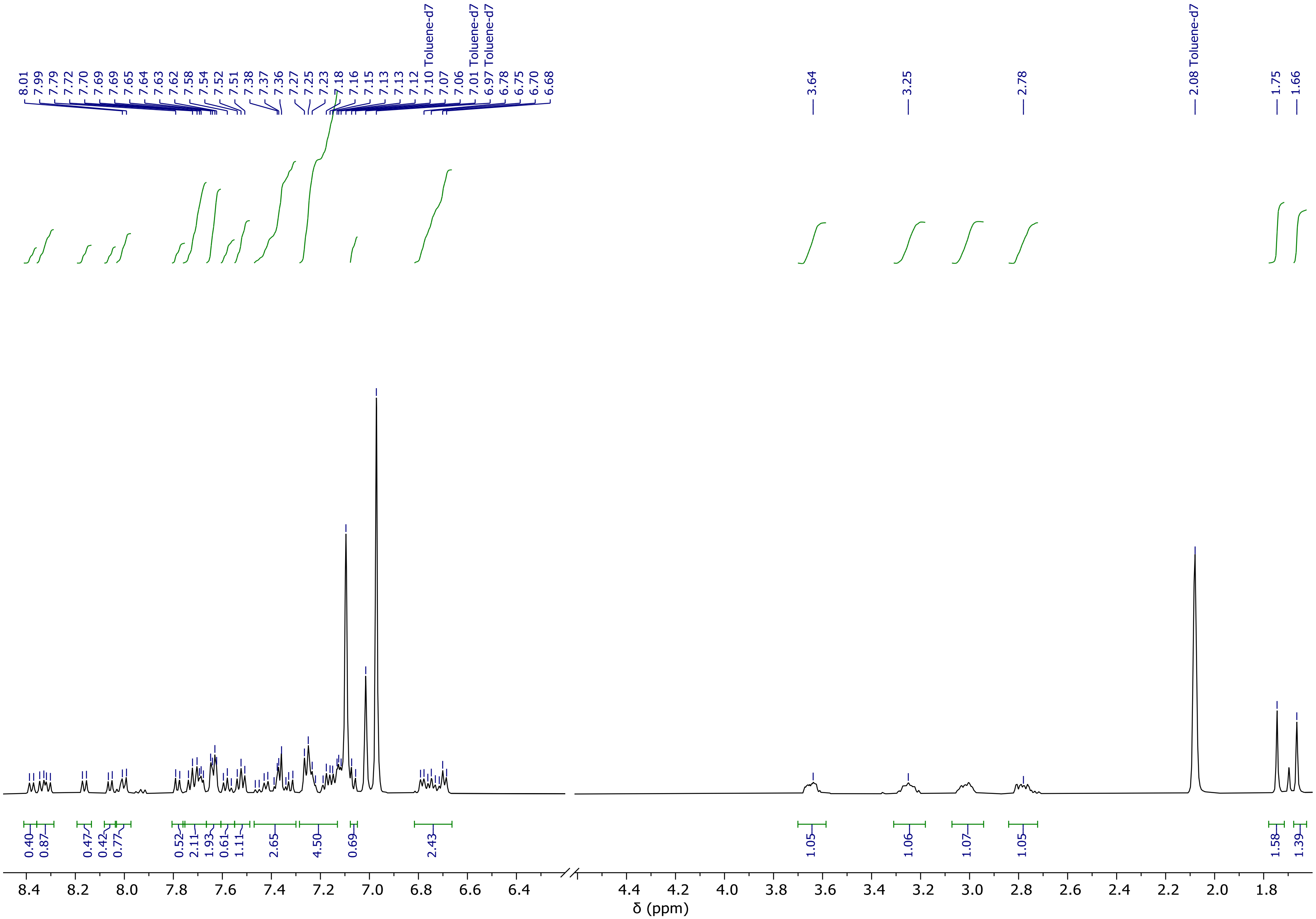

Figure S62. ^{1}H-NMR spectrum of sample enriched in the two stable isomers of M3 (500 MHz, Toluene-d8, 15 °C).

7. References

[1] J. Fang, L. Li, C. Yang, J. Chen, G.-J. Deng, H. Gong, *Org. Lett.* **2018**, *20*, 7308–7311.
[2] J.-L. Gras, *Org. Synth.* **1981**, *60*, 88.
[3] V. García-López, P.-T. Chiang, F. Chen, G. Ruan, A. A. Martí, A. B. Kolomeisky, G. Wang, J. M. Tour, *Nano Lett.* **2015**, *15*, 8229–8239.
[4] N. Assadi, S. Pogodin, S. Cohen, I. Agranat, *Struct. Chem.* **2013**, *24*, 1229–1240.
[5] F. Neese, F. Wennmohs, U. Becker, C. Riplinger, *J. Chem. Phys.* **2020**, *152*, 224108.
[6] S. Grimme, A. Hansen, S. Ehlert, J.-M. Mewes, *J. Chem. Phys.* **2021**, *154*, 064103.
[7] V. Barone, M. Cossi, *J. Phys. Chem. A* **1998**, *102*, 1995–2001.
[8] Y.-B. Zhao, B. Mariampillai, D. A. Candito, B. Laleu, M. Li, M. Lautens, *Angew. Chem. Int. Ed.* **2009**, *48*, 1849–1852.
[9] O. Trapp, *J. Chromatogr. B* **2008**, *875*, 42–47.
[10] M. Rickhaus, L. Jundt, M. Mayor, *CHIMIA* **2016**, *70*, 192–192.
